# Transcriptome and Secretome Analyses of Endophyte *Methylobacterium mesophilicum* and Pathogen *Xylella fastidiosa* Interacting Show Nutrient Competition

**DOI:** 10.3390/microorganisms11112755

**Published:** 2023-11-11

**Authors:** Manuella Nobrega Dourado, Paulo Marques Pierry, Oseias Rodrigues Feitosa-Junior, Guillermo Uceda-Campos, Deibs Barbosa, Paulo A. Zaini, Abhaya M. Dandekar, Aline Maria da Silva, Welington Luiz Araújo

**Affiliations:** 1 Microbiology Department, Biomedical Sciences Institute, University of Sao Paulo, Sao Paulo 05508-000, Brazil; 2 Agronomic Engineering College, University of Sorocaba, Sorocaba, Sao Paulo 18023-000, Brazil; 3 Biochemistry Department, Chemistry Institute, University of Sao Paulo, Sao Paulo 05508-000, Brazil; pmpierry@gmail.com (P.M.P.); oseias.rf.junior@gmail.com (O.R.F.-J.);; 4 Department of Plant Sciences, College of Agricultural and Environmental Sciences, University of California, Davis, CA 95616, USA; pazaini@ucdavis.edu (P.A.Z.);

**Keywords:** endophytic bacteria, citrus, phytopathogen–endophyte interaction, transcriptome, secretome

## Abstract

*Xylella fastidiosa* is the causal agent of several plant diseases affecting fruit and nut crops. *Methylobacterium mesophilicum* strain SR1.6/6 was isolated from *Citrus sinensis* and shown to promote plant growth by producing phytohormones, providing nutrients, inhibiting *X. fastidiosa*, and preventing Citrus Variegated Chlorosis. However, the molecular mechanisms involved in the interaction among these microbes are still unclear. The present work aimed to analyze physiological and molecular aspects of *M. mesophilicum* SR1.6/6 and *X. fastidiosa* 9a5c in co-culture. The transcriptome and secretome analyses indicated that *X. fastidiosa* down-regulates cell division and transport genes and up-regulates stress via induction of chaperones and pathogenicity-related genes including, the lipase-esterase LesA, a protease, as well as an oligopeptidase in response to *M. mesophilicum* competition. On the other hand, *M. mesophilicum* also down-regulated transport genes, except for iron uptake, which was up-regulated. Secretome analysis identified four proteins in *M. mesophilicum* exclusively produced in co-culture with *X. fastidiosa*, among these, three are related to phosphorous uptake. These results suggest that *M. mesophilicum* inhibits *X. fastidiosa* growth mainly due to nutrient competition for iron and phosphorous, thus promoting *X. fastidiosa* starvation, besides producing enzymes that degrade *X. fastidiosa* cell wall, mainly hydrolases. The understanding of these interactions provides a direction for control and management of the phytopathogen *X. fastidiosa*, and consequently, helps to improve citrus growth and productivity.

## 1. Introduction

*Xylella fastidiosa* (*Xf*) is a Gram-negative phytopathogenic bacterium (γ-Proteobacteria) found exclusively in the lumen of xylem vessels and is unable to colonize other plant tissues [1,2]. The main diseases caused by *Xf* are Citrus Variegated Chlorosis (CVC) [3], Pierce’s Disease in grapevines (PD) [4], and the Olive Quick Decline Syndrome (OQDS), which is an emerging disease in Europe [5,6]. Other diseases associated with *Xf* infection are denominated leaf scorch, which occurs in plum trees (PLS-plum), almond (ALS), coffee trees (CLS), peach (PLS-peach), oleander (OLS), mulberry, cherry trees, oak, elderberry, and hibiscus, among others [4,7,8]. This phytopathogen is transmitted by insect vectors from the *Cicadellidae* and *Cercopidae* families that feed on xylem sap [7,9].

Genomic and transcriptomic studies with *X. fastidiosa* have provided insights related to the biology of this phytopathogen, including the identification of genes potentially associated with virulence and pathogenicity [10,11,12,13,14,15,16]. These studies have been complemented with the description of proteomes and secretomes of *Xf* in different conditions [10,17,18,19,20]. Taken together these studies provided a detailed picture of *Xf* pathogenesis, including the effect of population density on the expression of virulence factors. A key mechanism in *Xf* pathogenesis is quorum sensing, which in *Xanthomonadaceae* is mediated by diffusible signaling factors (DSFs) [7,21]. DSFs can be detected in the bacterial surroundings both outside and associated with outer membrane vesicles (OMVs). OMVs also carry many proteins that can modify their microenvironments favoring bacterial survival, such as adhesins, lipases, proteases, and other hydrolases [19,22,23,24,25].

Proteomic analysis demonstrated that lipases/esterases are very abundant in the secretome of *Xf* strain Temecula1 [20]. The most abundant lipase, LesA (PD_1703/XFTem_01966) appears to act in the degradation of plant tissue, and its accumulation in leaf regions has been associated with the symptoms of Pierce’s disease of grapevines. A secretome study of *Xf* aiming to compare in vitro culture of the strains 9a5c (virulent in orange trees) and J1a12 (non-virulent) [19] revealed a distinct profile of extracellular proteins from both strains, totaling 71 different proteins, including the detection of LesA lipase-esterase (XF_0781/*XF*9a_00715) mainly in the virulent strain. One of the secretome proteins was the XfYgiT antitoxin (XF_2491/XF9a_02352), which in strain 9a5c is secreted inside OMVs [24] and is associated with biofilm formation, persistent cell generation, and decreased pathogenicity [26].

Inside the plant, *Xf* interacts with other microorganisms and reshapes microbial composition [27]. In citrus, it was reported that *Methylobacterium* species are dominant as endophytes within branches and interact with *Xf* [28,29,30]. The authors suggest that the presence of some endophytic *Methylobacterium* species in asymptomatic citrus tissues of plants with *Xf* could stimulate the production of compounds or elicitors that somehow increase plant resistance against *Xf* or decrease the phytopathogen growth [29,30]. In vitro and *in planta* experiments have shown that *Methylobacterium mesophilicum* (*Mm*) reduces *Xf* growth [29,31] which could be associated with the control of this bacterium in the host plant. Microarray analysis showed that *Xf* in the presence of *Mm* up-regulates genes related to stress and down-regulates genes related to bacterial growth [30]. Moreover, *Mm* is able to colonize not only different plant organs such as roots and leaves but also the lumen of xylem vessels, the same environment colonized by *Xf* [32], as well as the foregut of *Bucephalogonia xanthopis* [33] and *Homalodisca vitripennis* [34], both insect vectors associated to *Xf* transmission. All these data indicate that *Mm* and *Xf* could interact in different microenvironments during transmission and plant colonization.

*Methylobacterium* species have been isolated from more than 70 plant hosts [35] inducing plant growth promotion in citrus [36] and other plant species [37,38,39,40,41]. This effect on plant growth is due to the production of auxin and cytokinin phytohormones [41,42], induction of photosynthetic activity [43], and induction of systemic plant resistance by synthesizing pectinase and cellulase [37,44]. Members of this genus present a pink pigmentation due to carotenoid production [45] and can metabolize compounds with only one carbon such as methanol and methylamine [46] and are accordingly named Pink-Pigmented Facultative Methylotrophics (PPFM) bacteria.

In *Methylobacterium mesophilicum* (*Mm*), some key genes in bacterium-plant interactions were identified using quantitative PCR (qPCR) in rice and eucalyptus [47], and via transcriptomic analyses in soybean [48]. These studies showed that plant exudates up-regulate several genes involved in transport and stress, mainly with antioxidant functions. These results show how bacterial gene expression is regulated during plant-bacterium colonization, allowing the establishment of the interaction with the host plant and the associated microbiome.

Considering that the battle for ecological niches, including competition for habitat and resources, is a key challenge that bacteria face inside the host plant, different strategies to respond to competitors may have evolved in these microorganisms: direct responses (bactericide molecules) and/or indirect mechanisms (nutrient competition). Therefore, the main goal of the present work was to understand if the patterns of competitive and/or inhibitory phenotypes among *Mm* SR1.6/6 and *Xf* 9a5c populations in vitro could explain their interaction outcome inside the host plant. To do so, growth, transcriptome, and secretome were evaluated during the co-culture of these bacteria. This investigation provides valuable insights into the mechanism involved in the establishment of such a population inside the host plant and sheds light on *Xf* gene expression modulation in response to endophytic *Mm*, warranting more studies focused on the role of polymicrobial bacterial communities in the control/management of diseases caused by *Xf*.

## 2. Materials and Methods

### 2.1. Xf and Mm Cultivation Conditions

Experiments were performed with the endophyte *Mm* SR1.6/6 [28] and the phytopathogen *Xf* 9a5c [49], both isolated from *Citrus sinensis* (L.) Osbeck. These bacteria were grown in PW broth [50] supplemented with 0.5% glucose (PWG broth) at 28 °C under rotation of 170 rpm. The cultivation in solid medium was performed in PWG-1.5% agar. Cell stocks were maintained in PWG containing 50% of glycerol in a −80 °C freezer.

### 2.2. Bacterial Growth Evaluation

Bacterial growth was measured using OD_600nm_ in a NanoDrop 2000c spectrophotometer (ThermoFisher Scientific, Waltham, MA, USA). *Mm* SR1.6/6 and *Xf* 9a5c were cultured separately in 7.5 mL of PWG broth with an initial OD_600nm_ of 0.05. After achieving OD_600nm_ of 0.5, which was approximately 7 days for *Xf* and 2 days for *Mm*, 2.5 mL of each growth culture were aliquoted and *Xf* and *Mm* were mixed, totalizing a volume of 5 mL to each treatment: 1. Control: only *Xf*; 2. Control: only *Mm*; 3. Treatment: *Xf+Mm* co-culture. All cultures were then incubated at 28 °C under rotation of 170 rpm for 24 h, 48 h, and 72 h and OD_600nm_ was measured. Three biological replicates were performed.

### 2.3. Transcriptomic Analyses

#### 2.3.1. Experimental Design

Transcriptomes were evaluated for *Xf* and *Mm* monocultures and during co-culture. The two bacteria were cultivated separately in 75 mL of PWG, with an initial OD_600nm_ of 0.05. After achieving OD_600nm_ of 0.5, which was 7 days for *Xf* and 2 days for *Mm*, 25 mL of each culture were aliquoted and *Xf* and *Mm* were mixed, totaling 50 mL for controls (monoculture) and treatment (co-culture): 1. Control: only *Xf*; 2. Control: only *Mm*; 3. Treatment: *Xf+Mm* co-culture. All cultures were incubated at 28 °C and 170 rpm agitation for a period of 24 h. After this period, 40 mL of culture was centrifuged, and cell pellet was collected for RNA extraction followed by RNA-Seq. For OD_600nm_ measurements and DNA extraction for bacterial quantification by qPCR, culture aliquots were taken before mixing *Xf* and *Mm* cultures and after the co-culture period. All experiments were performed in biological triplicates, starting from independent cultures.

#### 2.3.2. qPCR Quantification of *Xf* and *Mm*

DNA was isolated using Wizard Genomic DNA Purification kit (Promega, Madison, WI, USA) according to the manufacturer’s recommendation. The qPCR amplification was performed in a StepOne Plus thermocycler (Applied Biosystems, Foster City, CA, USA) programmed to an initial denaturation of 95 °C for 3 min, followed by 40 cycles of 95 °C for 15 s and 60 °C for 30 s. The following primers were used: MMC1 (5′TACGTGGAGAGATTCACGGTC′3) and MMC2 (5′GTACAAGGCCCGGGAACGTAC′3) to quantify *Mm* SR1.6/6 [31]; and CVC-1 (5′AGATGAAAACAATCATCGAAA′3) and 272-2-int (5′GCCGCTTCGGAGAGCATTCCT′3) to quantify *Xf* 9a5c [51]. The amplification reaction had a final volume of 20 μL, with 2 μL of DNA (50 ng) and SYBR Master mix (Applied Biosystems, Foster City, CA, USA). Melting curves were analyzed in the PCR reaction to evaluate primer specificity using a temperature gradient from 72 °C to 96 °C, varying 0.5 °C every 15 s.

#### 2.3.3. Total RNA Isolation, cDNA Library Preparation, and Sequencing

Monocultures of *Xf* and *Mm* and co-culture of *Xf* and *Mm* collected after 24 h at 28 °C were centrifuged at 3220× *g* for 5 min at 4 °C. Supernatants were separated for proteomic analysis and cell pellets were used for RNA extraction. To disrupt bacterial cells, pellets were macerated with liquid nitrogen until they became a fine powder and subjected to total RNA isolation using TRIzol reagent (Invitrogen, Carlsbad, CA, USA) and Purelink RNA Mini Kit (Ambion, Foster City, CA, USA) according to instructions. The RNA samples were eluted in DEPC-treated water and stored at −80 °C freezer. The quantification and sample purity analyses were obtained in a NanoDrop 2000c spectrophotometer (ThermoFisher Scientific, Waltham, MA, USA). Integrity analyses were performed using 2100 BioAnalyzer and the RNA 6000 Nano kit (Agilent Technologies, Santa Clara, CA, USA). RIN (RNA Integrity Number) values above 7.0 were obtained and considered suitable for proceeding to the RNA-Seq analyses.

Total RNA samples were treated with DNase provided by the Illustra RNASpin Mini RNA isolation kit (GE Healthcare, Little Chalfont, UK), following instructions, although using double enzyme solution volume. Total RNA samples treated with DNase showed concentrations varying between 133 ng/μL to 275 ng/μL. Conventional PCR was performed with primers for a region of 16S rRNA gene [52]: S-D-Bact-0341-b-S-17 (5′CCTACGGGNGGCWGCAG3′) and S-D-Bact-0785-a-A-21 (5′GACTACHVGGGTATCTAATCC3′) and showed no residual genomic DNA (gDNA) in the RNA samples. RNA integrity analyses performed after DNase treatment to ensure the maintenance of high RIN values. Before initiating the library construction, RNA was quantified using Quant-iT RiboGreen RNA Assay kit (ThermoFisher Scientific, Waltham, MA, USA), and rRNA depletion was performed using Ribo-Zero Magnetic kit (Bacteria) (Illumina, San Diego, CA, USA). The depletion of rRNA molecules was evaluated through 2100 BioAnalyzer and the RNA 6000 Nano kit (Agilent Technologies, Santa Clara, CA, USA) and was considered satisfactory. cDNA libraries were constructed using TruSeq RNA sample preparation kit v2 (Illumina, San Diego, CA, USA). Libraries were analyzed through 2100 BioAnalyzer using a High Sensitivity DNA kit (Agilent Technologies, Santa Clara, CA, EUA) to determine the average fragment size. Libraries were quantified spectrophotometrically (Table S1) and using absolute qPCR in a 7500 Real-Time PCR System (Applied Biosystems, Foster City, CA, USA) with the Kapa Library Quantification kit for Illumina (Kapa Biosystems). All libraries were normalized to 4 nM, pooled to specific index combinations, denatured with NaOH 0.2 N, and incubated at 96 °C. Sequencing of cDNA libraries was performed in MiSeq equipment (Illumina, San Diego, CA, USA) with a final pool concentration of 8 pM (first run) and 10 pM (second and third run). All runs were performed with the MiSeq Reagent Kit v2 of 500 cycles with Paired-End 2 × 250 sequencing strategy. Cluster densities ranged from 676 to 882 K/mm^2^. The number and quality of reads per library was considered satisfactory (Table S2). The MiSeq equipment is located at the Center for Advanced Technologies in Genomics (CATG), Chemistry Institute, University of São Paulo, Brazil.

#### 2.3.4. Bioinformatic Analyses and Statistics

Read quality was assessed based on QScore using FASTQC software v0.11.6 [53]. Then, the reads were mapped to their respective reference genomes using the RNA-Seq analysis module of CLC Genomics Workbench 6.5 software (Qiagen, Germantown, USA). Reference genomes of *Xf* (9a5c) and *Mm* (SR1.6/6) were downloaded from the Genbank-NCBI using the assembly accession GCA_000006725 and GCA_000364445, respectively. Mapping was performed in two ways: generating normalized expression values of FPKM (Fragments Per Kilobase Per Million Mapped Fragments) [54] and generating gross counting values of how many reads were mapped to each gene. One criterion for counting mapped reads was the selection of those mapping only in one region of the genome, therefore duplicated regions in the genome were not considered to avoid error chances. FPKM values were used to calculate the Pearson correlation between biological replicates, while gross counting values to differential gene expression analysis between monocultures and co-culture transcriptomes. These analyses were performed using the R software package DESeq2 [55], considering genes with padj <0.1 and a log_2_FoldChange ≠ 0 as differentially expressed.

The sequences of differentially expressed genes (DEGs) encoding a protein were translated and used by Blast2GO software [56] to categorize them in their Gene Ontology terms (GO terms) [57]. Finally, Gene Ontology 2nd Level, a custom Python script available at https://oseias-r-junior.github.io/Gene_Ontology_2nd_Level (accessed on 15 March 2019), was used to retrieve the GO terms (at 2nd level) from queries provided by Blast2GO.

### 2.4. Secretomic Analyses

#### 2.4.1. Protein Extraction from *Xf*, *Mm*, and Co-Culture Supernatants

Supernatants of monoculture of *Xf* and *Mm* and co-culture of *Xf* and *Mm* from the transcriptomic experiments (Section 2.3.3) were collected, centrifuged at 3220× *g* for 30 min to remove the remaining bacterial cells and further concentrated 100-fold by ultrafiltration in Amicon Ultra 15 mL (Millipore) devices with Molecular Weight Cutoff (MWCO) of 3 kDa. Concentrated supernatants were sonicated (two pulses of 15 s with 30 s intervals under 4 °C) on Branson Sonifier 450 (Marshall Scientific, Hampton, NH, USA) and then performed a second centrifugation for 30 min at 12,000× *g* and 4 °C to remove remaining insoluble fraction. Total protein in the concentrated extracts was measured with the Bradford method.

The concentrated extracts obtained from supernatants of three biological replicates of *Xf* and *Mm* monocultures and *Xf* and *Mm* co-culture were pooled into single samples for each treatment, which in turn were mixed in the ratio 1:8:1 with the following solutions: 1 mL of the concentrated extract; 8 mL of ice-cold acetone (for HPLC, ≥99.9% purity) and 1 mL of trichloroacetic acid (100% TCA). Samples were maintained at −20 °C for 1 h until complete precipitation and then centrifuged at 18,000× *g* for 15 min at 4 °C. The supernatant was discarded, and the precipitate was washed with 1 mL of ice-cold acetone with complete resuspension and further centrifugation at 18,000 g for 15 min at 4 °C. Finally, after aspiration of the acetone, the precipitate was dried at room temperature and maintained at −20 °C until proteomic analysis. The total protein content of supernatant extracts was determined using Bradford reagent (Bio-Rad Laboratories Inc., Hercules, CA, USA), resulting in the following concentrations: 8.54 μg/μL for *Xf* monoculture, 16.11 μg/μL for *Mm* monoculture, and 9.03 μg/μL for the co-culture of *Xf* and *Mm*.

#### 2.4.2. Shotgun Proteomics

Proteomic analysis of extracted supernatants was performed as described previously [22,28], using a pool of three biological replicates from purified supernatants of *Xf* and *Mm* monocultures or co-cultures. The dried samples were reconstituted in PBS buffer and 300 μg of each sample were precipitated with 4 times the volume of ProteoExtract™ Protein Precipitation Kit (Calbiochem, San Diego, CA, USA) according to instructions. The samples were reconstituted in 100 μL of 6 M urea in 50 mM triethylammonium bicarbonate (TEAB) plus 5 mM dithiothreitol (DTT) and incubated at 37 °C for 30 min and shaking at 1000 rpm. Next, 15 mM iodoacetamide (IAA) was added, followed by incubation at room temperature for 30 min. The IAA was then neutralized with 30 mM DTT and incubated for 10 min. Lys-C/trypsin was added (1:25, enzyme: total protein) followed by incubation at 37 °C for 4 h. TEAB (550 μL of 50 mM) was added to dilute the urea and activate trypsin digestion overnight. The digested peptides were desalted with Aspire RP30 Desalting Tips (Thermo Scientific, Waltham, MA, USA), vacuum dried, and suspended in 45 μL of 50 mM TEAB. Peptides were quantified by Pierce quantitative fluorometric assay (Thermo Scientific) and 1 μg was analyzed on a QExactive mass spectrometer (Thermo Scientific) coupled with an Easy-LC source (Thermo Scientific) and a nanospray ionization source. The peptides were loaded onto a Trap (100 microns, C18 100 Å 5U) and desalted online prior to separation using a reversed-phase (75 microns, C18 200 Å 3U) column. The duration of the peptide separation gradient was 60 min using 0.1% formic acid and 100% acetonitrile (ACN) for solvents A and B, respectively. The data were acquired using a data-dependent MS/MS method, which had a full scan range of 300–1600 Da and a resolution of 70,000 Da. The resolution of the MS/MS method was 17,500 Da and the insulation width was 2 m/z with normalized collision energy. The nanospray source was operated using a spray voltage of 2.2 KV and a transfer capillary temperature heated to 250 °C.

The raw data were analyzed using X!Tandem and viewed using the Scaffold Proteome Software version 4.0 (Proteome Software Inc., Portland, OR, USA) [58]. Samples were searched against UniProt databases appended with the cRAP database, which recognizes common laboratory contaminants. Reverse decoy databases were also applied to the database before the X!Tandem searches. The annotated genomes of *Xf* 9a5c and *Mm* SR1.6/6 were used as references for identification of the proteins. The proteins identified were filtered (in the Scaffold Proteome Software) based on the following criteria: 1.0% FDR (False Discovery Rate) at protein level (following the prophet algorithm: http://proteinprophet.sourceforge.net/ (accessed on 11 September 2023)), minimum number of 2 peptides and 0.1% FDR at peptide level. Protein sequences identified were examined for the presence of a signal peptide [59] and the potential (SecP score) of being secreted by a non-classical protein secretion system [60]. Similar to the transcriptomic analysis, the tool Gene Ontology 2nd Level was used to retrieve the GO terms from queries provided by Blast2GO software. 

### 2.5. Integration of Transcriptomics and Proteomics Analyses

For integration of transcriptomics and proteomics analyses, FPKM values were converted to TPM (Transcripts Per Kilobase Million) values, as reported in the literature [61], and only nonzero TPM values were considered. Further, the means of the three replicates TPM values were calculated. In turn, proteomic data available as spectra counts were also normalized. The spectra counts were divided by respective protein size (NC = SC/PS×1000). Next, the normalized counts (NC) were divided by the secretome size (PPT = NC*_i_*/∑NC×1000) retrieving a final normalized number of called peptides per thousand (PPT). Secretome samples were from a pool of three biological replicates. Following this, a Venn diagram was obtained with the Venn Python library to assign intersection gene products detected in transcriptome and secretome [62]. Euclidean distances were calculated between *Xf* or *Mm* co-culture versus their respective monoculture common gene products. The Mantel test was used to calculate the correlation between two matrices [63] either transcriptome or secretome data. The Mantel test was performed using a MantelTest Phyton library which returns a correlation coefficient, *p*-value, and a standard score (z-score). Further, for functional analysis, genes with similar trends (i.e., up- or down-regulated) in both transcriptome and secretome were subjected to GO terms annotation. These GO terms were used to build a GO enrichment network using the Cytoscape software v3.10.1 [64].

## 3. Results

### 3.1. Growth of Mm and Xf in Co-Culture and Xf Biofilm Formation

**Figure 1:**
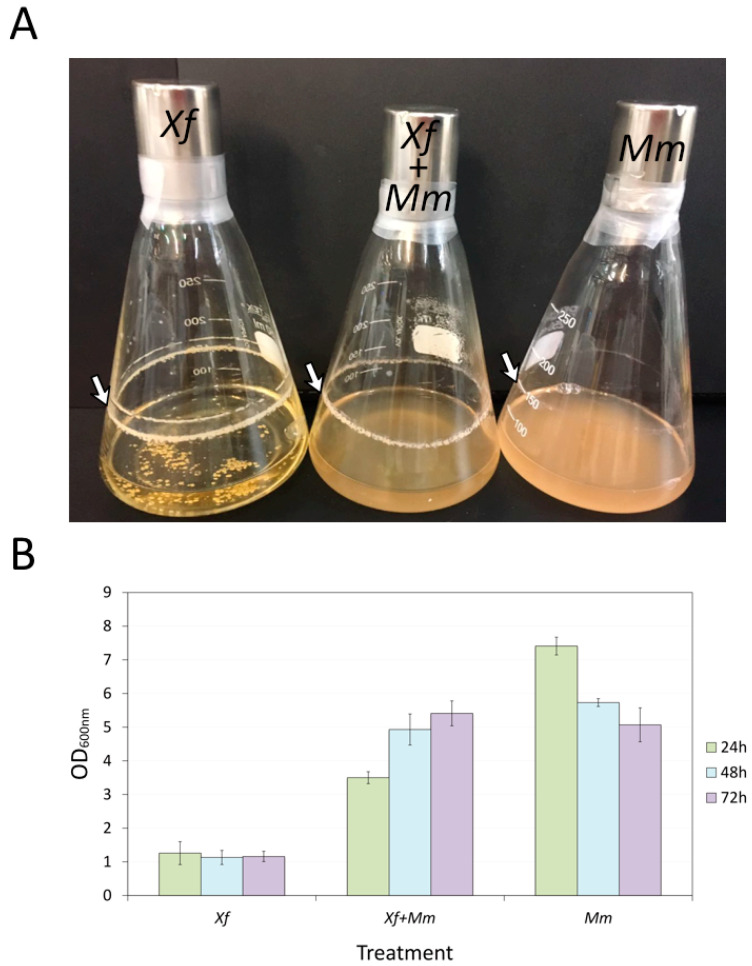
Phenotypic and growth evaluation of *Xf* and *Mm* monocultures and co-culture. (**A**) Phenotypic growth and biofilm formation (indicated by the white arrow) of *Xf*, *Xf*+*Mm*, and *Mm* after 72 h of culture. (**B**) Quantification of bacteria growth. Measurements of OD_600nm_ of each treatment before and after co-culture. Measurements were performed after 24 h, 48 h, and 72 h of culture. *Xf*: *X. fastidiosa* 9a5c; *Xf*+*Mm*: *X. fastidiosa* 9a5c and *M. mesophilicum* SR1.6/6; *Mm*: *M. mesophilicum* SR1.6/6.

An initial experiment was performed to evaluate endophyte and pathogen growth in monoculture and co-culture for 24 h, 48 h, and 72 h in PWG broth (Figure 1) to investigate the interaction between these bacteria isolated from citrus. Figure 1A shows biofilm formation in the three cultures *Xf*, *Mm*, and co-culture, with *Xf* forming a thicker biofilm. The results also show that *Xf* monoculture density was stable over the time evaluated, while that of the co-culture (*Xf+Mm*) increased, and *Mm* monoculture decreased after 24 h (Figure 1B).

### 3.2. Bacteria Quantification by qPCR

**Figure 2:**
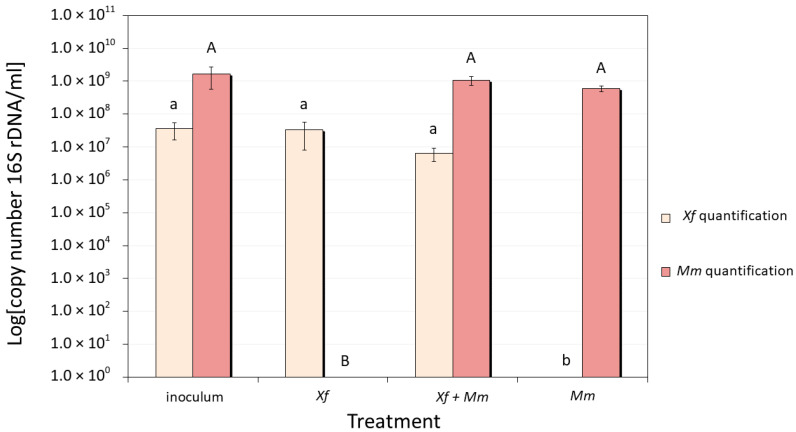
Quantification by qPCR of the number of copies of specific genes in aliquots of bacterial cells used in RNA-Seq analyses (after 24 h of co-culture). *M. mesophilicum* SR1.6/6 quantification using the pair of primers MMC1/MMC2 [31]; and *X. fastidiosa* 9a5c quantification using the pair of primers CVC-1/272-2-int [51]. *Xf*: *X. fastidiosa* 9a5c monoculture; *Xf*+*Mm: X. fastidiosa* 9a5c and *M. mesophilicum* SR1.6/6 co-culture; *Mm*: *M. mesophilicum* SR1.6/6 monoculture. Statistical analyses were performed using the Duncan test. Capital letters show statistical differences when comparing *Mm* quantification and lower-case letters when comparing *Xf* quantification, different letters indicate statistically significant different mean.

Aliquots of bacterial cultures for RNA-Seq analyses were also used to isolate DNA to quantify *Mm* and *Xf* gene copy numbers. The initial inoculum of *Xf* and *Mm* present similar growth verified by the OD_600nm_ measurement. After 24 h, in control cultures (*Xf* or *Mm* monocultures) and co-culture (*Xf* and *Mm*), quantification showed no significant alterations in the number of cells for both *Mm* and *Xf* when comparing with the inoculum (Figure 2), contributing to reduced transcriptomic analyses variation.

### 3.3. Transcriptomic Analyses 

A total of 4,400,994 reads of *Xf* in monoculture, 3,981,063 reads of *Mm* in monoculture, and 4,408,696 reads of *Xf+Mm* co-culture, all of them after 24 h incubation, were obtained from cDNA library sequencing. The reads were mapped on the reference *Xf* 9a5c and *Mm* SR1.6/6 genomes, which present 2,679,305 bp (2708 genes including plasmids) and 6,214,449 bp (5899 genes), respectively. The average percentage of uniquely mapped reads was high in both *Xf* (80.16%) and *Mm* (71.45%) monoculture, while in the co-cultures the percentage was lower (51.05% in *Xf* and 26.41% in *Mm*) (Table 1 and Table S3).

**Figure 3:**
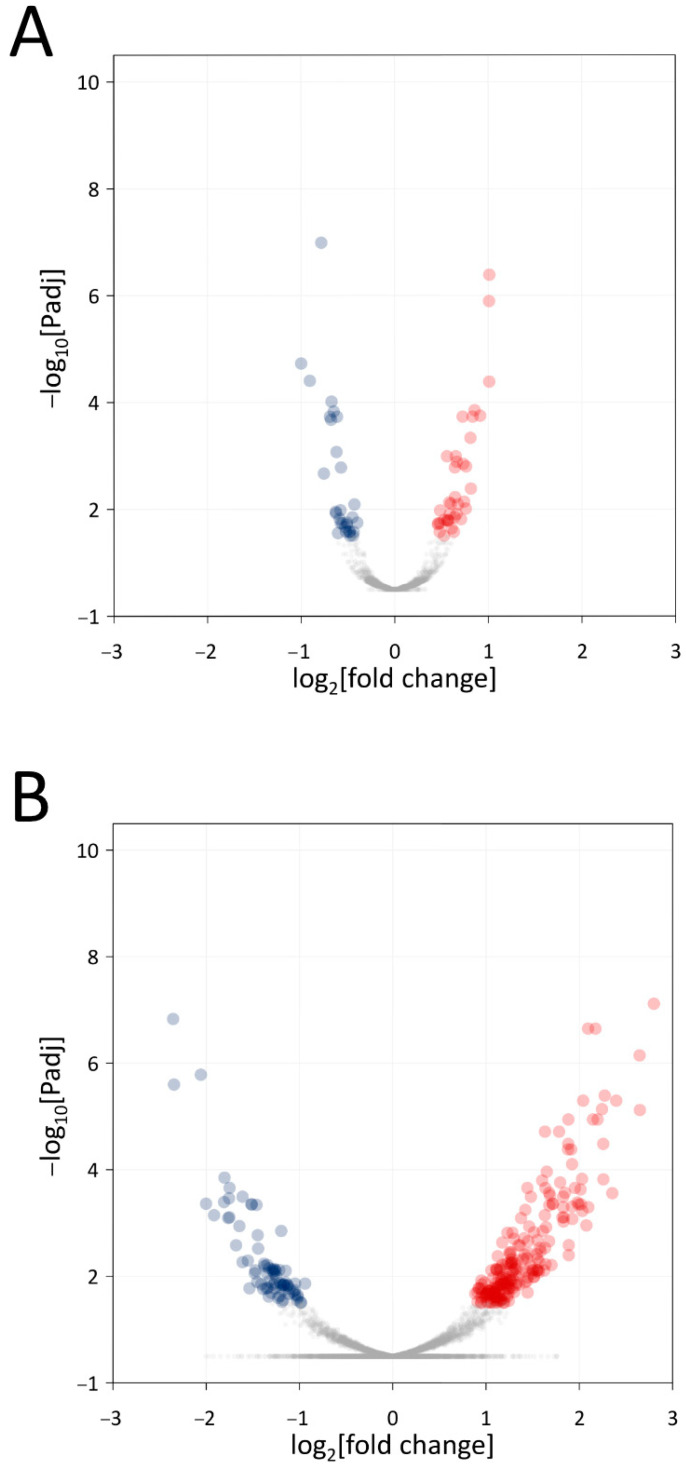
Volcano plots of differentially expressed genes. (**A**). Results of the comparison between the *Xf+Mm* co-culture with the control (*Xf* monoculture) (**B**). Results of the comparison between the *Xf+Mm* co-culture with the control (*Mm* monoculture). In the x-axis are the log_2_fold change values; in the y-axis are the −log_10_ adjusted *p*-values (padj). Points in red and blue represent genes with padj < 0.1, and thus, considered statistically differentially expressed; red dots represent genes up-regulated, while blue dots represent genes down-regulated. *Xf*: *X. fastidiosa* 9a5c; *Xf*+*Mm*: *X. fastidiosa* 9a5c and *M. mesophilicum* SR1.6/6; *Mm*: *M. mesophilicum* SR1.6/6.

**Figure 4:**
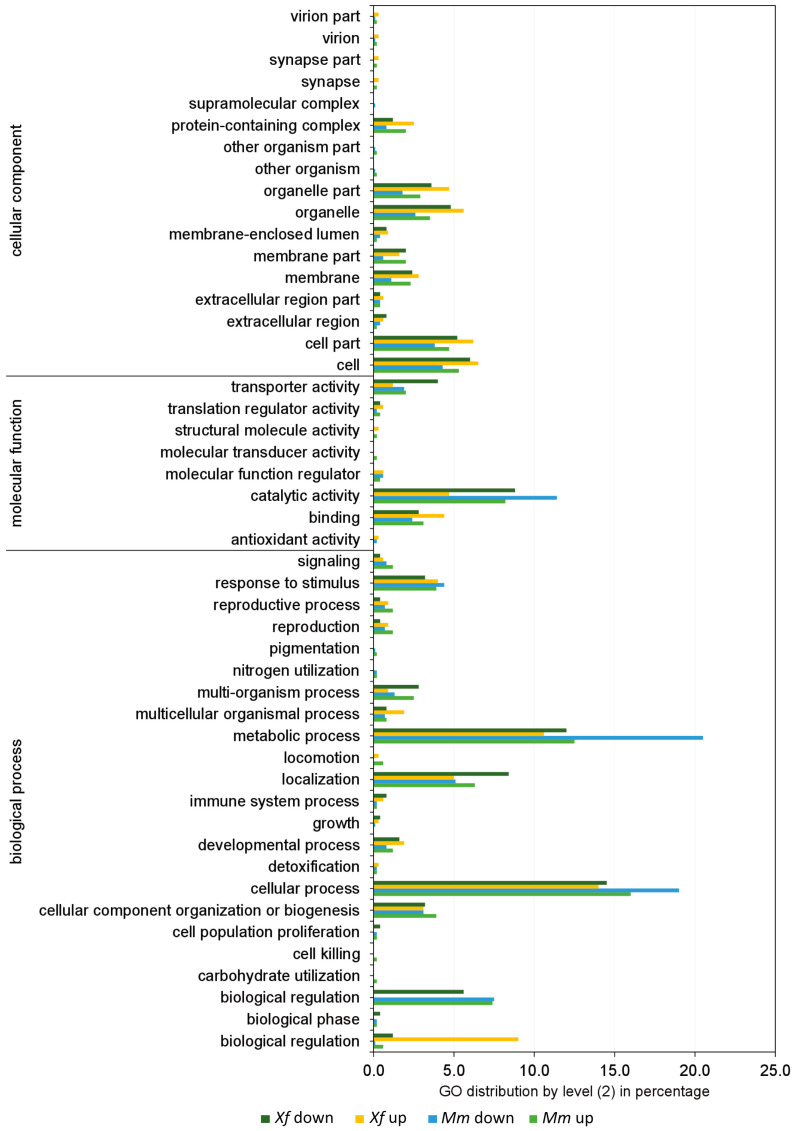
Frequency (%) of the categories of differentially expressed genes (up- and down-regulated) in co-culture generated by Blast2GO software. *Xf* down: genes down-regulated in *Xf* in the comparison between the co-culture (*Xf*+*Mm*) and the control (*Xf*); *Xf* up: genes up-regulated in *Xf* in the comparison between the co-culture (*Xf*+*Mm*) and the control (*Xf* monoculture). *Mm* down: genes down-regulated in *Mm* in the comparison between the co-culture (*Xf*+*Mm*) and the control (*Mm* monoculture); *Mm* up: genes up-regulated in *Mm* in the comparison between the co-culture (*Xf*+*Mm*) and the control (*Mm* monoculture). Amino acid sequences of differentially expressed genes were used for annotation by “biological process”, “molecular function” and “cellular component”. *Xf*: *X. fastidiosa* 9a5c; *Xf*+*Mm*: *X. fastidiosa* 9a5c and *M. mesophilicum* SR1.6/6; *Mm*: *M. mesophilicum* SR1.6/6.

We identified 68 differentially expressed genes (DEGs) in *Xf* and 288 DEGs in *Mm* monoculture in comparison with *Xf* and *Mm* co-culture (Figure 3). The complete lists of differentially expressed genes for *Xf* and *Mm* are shown in Supplementary Tables S4 and S5, respectively. The DEGs identified in monocultures of *Xf* and *Mm* and co-culture treatments were annotated according to the Gene Ontology (GO) (Figure 3 and Figure 4). We highlight the presence of DEGs (both up- and down-regulated) related to the categories “molecular function” (catalytic activity, transport activity, and binding), and “biological process” (cellular and metabolic process, biological regulation, and response to stimulus) in both *Xf* and *Mm* cultures (Figure 3 and Figure 4).

### 3.4. Transcriptome Analysis of Xf+Mm Co-Culture Compared to Xf Monoculture

The following sections describe genes related to selected functional categories that were differentially expressed by comparing *Xf* monoculture to *Xf+Mm* co-culture transcriptomes.

#### 3.4.1. Macromolecule Metabolism 

Genes related to RNA metabolism were up-regulated during co-culture. All genes of protein metabolism related to tRNA were down-regulated. All transcripts related to cell division were down-regulated, such as predicted integral membrane proteins containing uncharacterized repeats (XF_2349/XF9a_02226), cell division septal protein (XF_0800/XF9a_00732), and a MAF protein (XF_1124/XF9a_01036) (Table S4).

#### 3.4.2. Transport

Overall transport proteins (for sugars, amino acids and ions) were down-regulated: Mg^2+^ and Co^2+^ transporters (XF_0900/XF9a_00833), sulfate ABC transporters (XF_1346/XF9a_01231), permeases (XF_0139/XF9a_00121), oligopeptide transporter, OPT Family (XF_2261/XF9a_02149) and transporter, as well as the SSS (solute:sodium symporter) (XF_2251/XF9a_02141). However, a single transporter was up-regulated, the phosphate ABC transporter, phosphate-binding protein (XF_2141/XF9a_02027), which primarily imports nutrients during phosphate starvation (Table S4).

#### 3.4.3. Stress-Related Genes

Several *Xf* chaperone genes were up-regulated during co-culture: molecular chaperone, HSP90 family (XF_0978/XF9a_00903), chaperonin GroEL (XF_0615/XF9a_00557), cochaperonin GroES (HSP10) (XF_0616/XF9a_00558), chaperone protein DnaK (XF_2340/XF9a_02217), molecular chaperone GrpE (heat shock protein) (XF_2341/XF9a_02218) and Zn-dependent protease with chaperone function (XF_2625/XF9a_02481). Meanwhile, three *Xf* stress-related genes were down-regulated: ascorbate metabolism—2,3-diketo-L-gulonate reductase (XF_2449/XF9a_02311), glutathione metabolism—lactoylglutathione lyase (XF_1399/XF9a_01278) and stress-induced morphogen (activity unknown) (XF_2450/XF9a_02312) (Table S4).

#### 3.4.4. Pathogenicity

Genes related to membrane lysis were up-regulated in *Xf* during co-culture: esterase/lipase (XF_0357/XF9a_00323), protease (XF_0185/XF9a_00171) and Zn-dependent oligopeptidase (XF_1944/XF9a_01835). Instead, genes coding for iron storage proteins such as bacterioferritin (XF_0395/XF9a_00357) and copper-binding protein of YfiH family (XF_0940/XF9a_00870), as well as membrane and biofilm-related genes, such as an adhesin (XF_1529/XF9a_01401), a lipoprotein (XF_2185/XF9a_02068) and a lysozyme activity protein (XF_0907/XF9a_00840) were down-regulated in *Xf* during co-culture (Table S4).

### 3.5. Transcriptome Analysis of Xf+Mm Co-Culture Compared to Mm Monoculture

The following sections describe genes related to selected functional categories that were differentially expressed by comparing Mm monoculture to *Xf+Mm* co-culture transcriptomes.

#### 3.5.1. DNA, RNA, and Protein Metabolism

Overall, genes related to DNA replication and DNA repair were down-regulated. Several transcriptional regulators were down-regulated in *Mm* during co-culture: MucR family transcriptional regulator, related to symbiosis and exopolysaccharide production; MerR family DNA-binding transcriptional regulator (MMSR116_RS14485), related to metal sensing; Crp/Fnr family transcriptional regulator (MMSR116_RS00375) and bifunctional DNA-binding transcriptional regulator/O6-methylguanine-DNA methyltransferase Ada (MMSR116_RS30165), related to the activation of stress-related genes. 

A general transcriptional regulator (MMSR116_RS08620), an endonuclease (MMSR116_RS22505), the RNA polymerase sigma factor RpoH (MMSR116_RS11170), a methyltransferase domain-containing protein (MMSR116_RS23260), a DNA-binding response regulator (MMSR116_RS06015) and the nitrogen regulatory protein P-II (MMSR116_RS28615) were also down-regulated. Genes related to translation were up-regulated such as ribosomal proteins. Cell division genes were also up-regulated in *Mm* during co-culture (Table S5).

#### 3.5.2. Lipid, Sugar, Amino Acid and Nucleotide Metabolism

Other down-regulated genes include those of amino acid biosynthesis functions: the L-homocysteine biosynthesis gene Adenosylhomocysteinase (MMSR116_RS19390); serine biosynthesis genes, such as D-serine ammonia-lyase (MMSR116_RS11755), serine protease (MMSR116_RS01620), serine O-acetyltransferase (MMSR116_RS25615), and PrkA family serine protein kinase (MMSR116_RS24210); besides other amino acids related genes: a hypothetical protein (MMSR116_RS21385), a 3-keto-5-aminohexanoate cleavage protein (MMSR116_RS29585), an aspartate ammonia-lyase (MMSR116_RS09695), an aspartate-semialdehyde dehydrogenase (MMSR116_RS07215), a D-amino acid dehydrogenase (MMSR116_RS01745) and a methylmalonate-semialdehyde dehydrogenase (CoA acylating) (MMSR116_RS25555).

Purine and pyrimidine metabolism genes were also down-regulated: xanthine dehydrogenase family protein (MMSR116_RS22755), hypothetical protein (MMSR116_RS29495), NUDIX domain-containing protein (MMSR116_RS09635), NMT1/THI5-like domain-containing protein (MMSR116_RS15475) and polyphosphate kinase 2 (MMSR116_RS14700) (Table S5).

#### 3.5.3. Transport

Fifteen genes related to transport were down-regulated in *Mm* during co-culture. Among them are genes for ion transporters: mechanosensitive ion channel family protein (MMSR116_RS24965), oxalate/formate MFS antiporter (MMSR116_RS02620), STAS/SEC14 domain-containing protein (MMSR116_RS21710), citrate transporter (MMSR116_RS16110), SulP family inorganic anion transporter (MMSR116_RS01695) and a putative sulfate exporter family transporter (MMSR116_RS08495). Other transporters were found related to carbohydrate transport uptake, such as the PTS fructose transporter subunit IIA (MMSR116_RS15270); protein transport, such as a nuclear transport factor 2 family protein (MMSR116_RS16730); amino acid transport: ABC transporter substrate-binding protein (MMSR116_RS29515, MMSR116_RS29475), and a dicarboxylate/amino acid:cation symporter (MMSR116_RS20220); and general transporters, such as MFS transporter (MMSR116_RS22290, MMSR116_RS27205, MMSR116_RS21120, MMSR116_RS07435).

On the other hand, siderophore transport genes were up-regulated in *Mm*: TonB-dependent siderophore receptor (MMSR116_RS23315, MMSR116_RS21805), TonB-dependent receptor (MMSR116_RS13925), iron ABC transporter permease (MMSR116_RS08120), and the porins MMSR116_RS12465, MMSR116_RS12045, MMSR116_RS03325, and MMSR116_RS25895 (Table S5).

#### 3.5.4. Stress-Related Genes

Eighteen stress-related genes were down-regulated in *Mm* during co-culture. Most of them are related to glutathione, thioredoxin, and the metabolism of iron-sulfur clusters.

Chemotaxis-related genes were up-regulated in *Mm*: HAMP domain-containing protein (MMSR116_RS22055), chemotaxis phosphatase CheZ (MMSR116_RS26365), and a response regulator (MMSR116_RS26370). Cell adhesion genes were also up-regulated in *Mm*: the outer membrane protein assembly factors BamA (MMSR116_RS12315) and BamE (MMSR116_RS22310) (Table S5).

#### 3.5.5. Defense Mechanism-Related Genes

Enzymes related to cell wall and membrane cleavage were up-regulated in *Mm* during co-culture: cell wall hydrolase SleB (MMSR116_RS01255), glycoside hydrolase (MMSR116_RS03180), a hypothetical protein (MMSR116_RS14565) and lytic transglycosylase domain-containing protein (MMSR116_RS03845) (Table S5). 

### 3.6. Secretome Analyses 

#### 3.6.1. Total Secretome of *Xf* and *Mm*

A total of 112 unique proteins were detected across the secretome samples analyzed by LC-MS/MS and identified by two or more peptides. More *Xf* proteins were detected in the secretome samples compared to *Mm* (64 and 48 proteins, respectively). The secretome of *Mm* and *Xf* are detailed in Table 2 and Table 3, respectively. Fifty-five proteins were identified in the co-culture, of which 32 were from *Xf* and 23 were from *Mm*. None of the proteins detected for *Xf* in co-culture was exclusive to this treatment. On the other hand, four *Mm* proteins were detected only in the co-culture as reported below (Table 3).

#### 3.6.2. *Xf* Secretome

There were no exclusive proteins from *Xf* in its co-culture with *Mm*, meaning that all those detected in this treatment were also found in the *Xf* monoculture. However, many proteins detected in the *Xf* monoculture secretome were not detected in the co-culture secretome (Table 2). Among the proteins absent in co-culture, we highlight those linked to carbon metabolism, defense mechanisms, and translation. The most abundant proteins found in both *Xf* monoculture and co-culture were membrane proteins: Omp1X (XF_1803/XF9a_01687), membrane lipoprotein Lpp (XF_1547/XF9a_01416), and outer membrane protein mopB (XF_0343/XF9a_00315).

#### 3.6.3. *Mm* Secretome

**Figure 5:**
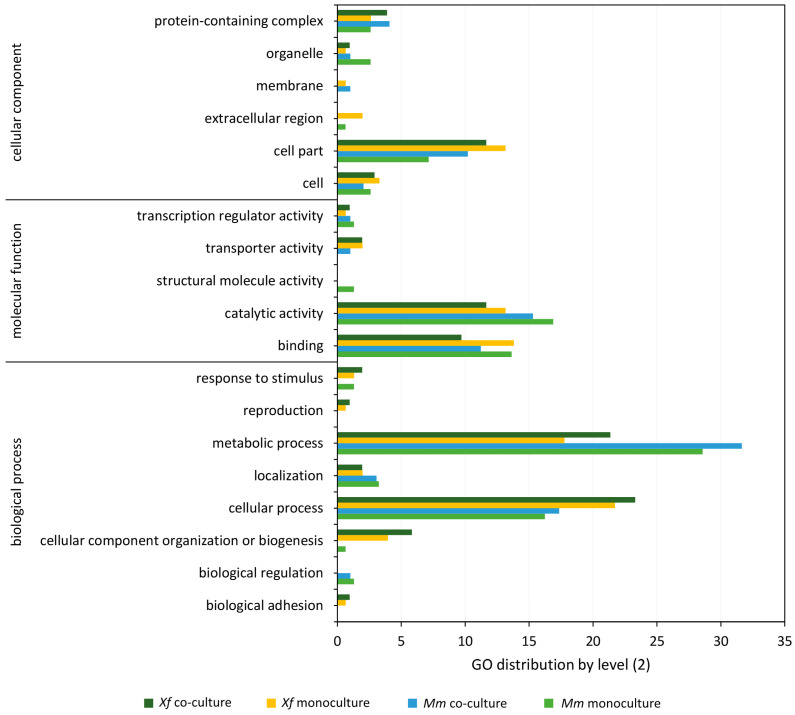
Gene Ontology classifications of secretome proteins. Proteins detected in the secretome analysis were annotated using Blast2GO (version 5.2.5) software in level 2 GO terms for Cellular Component, Molecular Function, and Biological Process ontologies. The percentage of annotated proteins with each indicated GO term level 2 is shown. The classification was applied to the secretomes of the monoculture of *X. fastidiosa*, *X. fastidiosa* on co-culture with *M. mesophilicum*, the monoculture of *M. mesophilicum* and *M. mesophilicum* on co-culture with *X. fastidiosa*.

In the *Mm* secretome, the following proteins were detected under monoculture and co-culture: porins, oxidative stress proteins (thioredoxin), and chaperones (GroES and DnaK). Flagellin, a motility-related protein, is the most abundant protein for *Mm*. There are four exclusive proteins in the co-culture for *Mm*: the inorganic pyrophosphatase Ppa (MMSR116_RS21075), the adenosylhomocysteinase AhcY (MMSR116_RS19390), the phosphate-binding protein PstS (MMSR116_RS19735) and a porin (MMSR116_18145) (Table 3 and Figure 5). On the other hand, in *Mm* monoculture, there were 25 exclusive proteins. MMSR116_RS18845, flagellin, is related to cell motility and is also a microbe-associated molecular pattern (MAMP). Several uncharacterized proteins and the majority of metabolism proteins were also found.

#### 3.6.4. Secretome Predictions and Functional Analyses 

The prediction tools SignalP and SecretomeP analysis revealed 26 out of a total of 55 proteins (~58%) present in the co-culture secretome to have known secretion prediction. Additionally, 20 cytoplasmic or other non-predicted secreted proteins were detected in the *Mm* secretome, as compared to 16 in the *Xf* secretome in the co-culture. For *Mm* and *Xf* monoculture secretomes, respectively, 37 (67%) and 48 (75%) detected proteins have secretion prediction.

Significant differences were observed between the secretomes of *Xf* and *Mm* in the co-culture, as compared to their corresponding monocultures. In the co-culture, the number of detected proteins for *Xf* and *Mm* decreased by around half, resulting in 32 and 23 proteins, respectively, compared to their monocultures, which had 64 (*Xf*) and 44 (*Mm*) proteins. Furthermore, while the ranking of the top 20 most abundant proteins for *Xf* and *Mm* monocultures changed in terms of presence or absence in the co-culture, the majority of these top 20 proteins remained consistent for both *Xf* and *Mm*.

Further, we annotated the proteins detected at the secretome of *Xf* and *Mm* according to GO, consistent with our previous transcriptomic analyses (Figure 5). Between 1 to 4% of the proteins from the full secretome (monoculture or co-culture, *Xf* or *Mm*) submitted returned no GO terms (including hypothetical proteins). 

For *Xf* secretome, 51 out of 64 abundant proteins detected (80%) in the monoculture, and 24 out of 32 abundant proteins detected (75%) in the co-culture were assigned to the GO level 2 categories (Figure 5) using the tool Gene Ontology 2nd Level (https://oseias-r-junior.github.io/Gene_Ontology_2nd_Level/). GO annotation of *Mm* proteins assigned 28 out of 44 abundant proteins detected (64%) in the monoculture and 20 out of 23 abundant proteins detected (87%) in the co-culture with the GO 2nd level categories (Figure 5). Most of these gene ontology terms for both *Xf* and *Mm* were distributed into more specific GO-level subcategories. GO categories distribution follows a similar pattern as in RNA-Seq analyses, with most proteins grouped into cellular process and metabolic process (BP) and catalytic activity (MF).

GO categories “binding” and “catalytic activity” are higher for *Xf* in the co-culture compared to monoculture, but lower for Mm in the same comparison. “binding” and “catalytic activity” are usually associated with the description of enzymes. “Response to stimulus” was only found on *Mm* and *Xf* monocultures and “biological adhesion” was present only on *Xf* monoculture. Moreover, “biological regulation” was not detected in *Xf* co-culture.

### 3.7. Integrating Transcriptome and Secretome Data

Usually omics integration is performed through the comparison between differentially expressed genes (DEGs) which are common among omics data [65]. In this work, although we found DEGs for *Xf* or *Mm* transcriptome analyses we did not access differentially expressed proteins for *Xf* or *Mm* secretome analysis. Thus, we adopted a strategy to search for correlation and major trends between the two techniques, similar to what have been done before [66]. 

**Figure 6:**
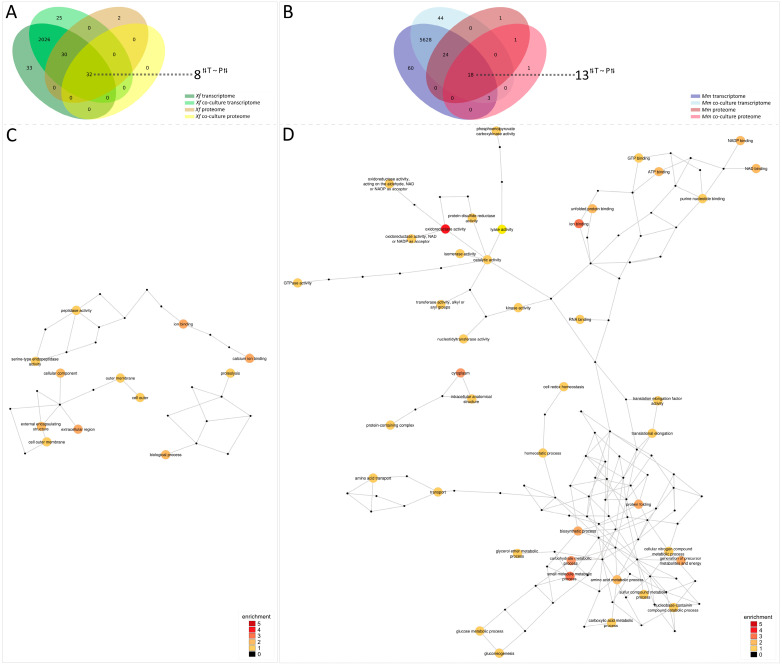
Integration of transcriptome and secretome analyses. Venn diagrams show the intersection between transcriptome and proteome as well as monoculture and co-culture from *Xf* (**A**) and *Mm* (**B**). Numbers highlighted outside the Venn diagrams indicate the intersection of genes with the same trend, illustrated by the divergent arrows, in the transcriptome (T) and secretome (S). *Xf*: *X. fastidiosa* 9a5c; *Xf*+*Mm*: *X. fastidiosa* 9a5c + *M. mesophilicum* SR1.6/6; *Mm*: *M. mesophilicum* SR1.6/6. GO terms networks of *Xf* (**C**) and *Mm* (**D**) interaction data. Networks representing gene ontology (GO) terms in molecular function, biological process, or cellular component categories enriched among the integration of transcriptome and secretome analyses, showing genes specifically affected by co-culture between *Mm* and *Xf*. Enriched GO terms were identified after a pipeline involving a Mantel correlation test and Blast2Go and visualized with Cytoscape software v3.10.1. The GO terms were connected based on their parent-child relationships. The colors of the circles indicate the GO terms filtered after the interactome pipeline.

We found 32 common genes between the two analyzed secretomes and the two analyzed transcriptomes of co-culture versus monoculture from *Xf*. Regarding the comparison of co-culture versus monoculture from *Mm*, 18 common genes were detected (Figure 6A,B). To establish an appropriate comparison with transcriptome data, the proteome data were normalized to PPT values similarly to TPM values, as described in the Materials and Methods section. Following this, a Mantel test was used to obtain a correlation between the secretome and transcriptome. The intersection genes detected in transcriptome and secretome (32) from *Xf* have a correlation shown by a *p*-value of 0.05742, which indicates a medium-to-high correlation. A much lower correlation was obtained in the intersection genes detected in transcriptome and secretome (18) from *Mm* where a *p*-value ≅ of 0.8038 was calculated based also on a Mantel test. By the removal of the outlier gene MMSR116_RS20190 (Table S7), we calculated a much smaller *p*-value of 0.08273, indicating a relative medium-to-high correlation between transcriptome and secretome also for *Mm*.

Following, the intersection genes from both *Xf* and *Mm* were further analyzed. The ratios between gene values in the transcriptome (co-culture:monoculture) were compared to the ratios of gene products values in the secretome (co-culture:monoculture), i.e., TPM and PPT, respectively. Gene transcripts that were up- or down-regulated in a given condition do not always result in up- or down-regulation at the protein level [65]. Therefore, we again limited our observations to major trends. Ratios in transcriptome and secretome that simultaneously were more than one or less than one were observed in 8 out of 32 genes for *Xf* and in 13 out of 18 genes for *Mm* (Tables S6 and S7). These genes with conserved trends for GO annotations were then used to build networks (Figure 6C,D and Figure S1). The GO annotations network for *Xf* showed particularly two different functionally enriched annotations: extracellular region, and calcium ion binding/ion binding. On the other hand, *Mm* had generated a huge GO network, which was particularly enriched in GO annotations associated with oxidoreductase activity, ion binding, and small molecule metabolic process.

## 4. Discussion

Gene expression profiles can reveal valuable information about the mechanisms related to interactions among bacterial species in a particular environmental niche. Several endophytic bacteria can occupy the same niche/habitat as the phytopathogen *Xf* in the lumen of xylem vessels [27,28,31,32]. Araujo et al. [28] isolated a high number of *Methylobacterium* spp. in asymptomatic *Xf*-infected citrus plants. Moreover, physiological tests were performed showing the growth inhibition of the phytopathogen *Xf* in the presence of *M. mesophilicum*, *M. extorquens*, and *Curtobacterium* sp. in vitro [29] and in the presence of *Mm in planta* [31]. Induction of stress-related genes and the repression of growth genes in *Xf* were detected in co-culture with *Mm* [30]. However, the DEG of *Mm* as well as new *Xf*-expressed genes were not assessed in that initial study. The genome sequences of both species are available, improving transcriptomic and proteomic analyses. Recently, a transcriptomic study of *Mm* SR1.6/6 interacting with soybean was performed [48], which has analyzed planktonic bacterial cells under the influence of soybean exudates in comparison with control treatments, showing that several stress genes are modulated by plant colonization. Here we investigated the transcriptomic response of both interacting species using RNA-seq and complemented this dataset with the secretome of both bacterial species. We hypothesized that the co-culture of *Mm* with *Xf* would generate nutrient deprivation for both bacteria, as previous works in the literature suggested environmental niche competition by *Mm* towards *Xf* [29,30].

**Figure 7:**
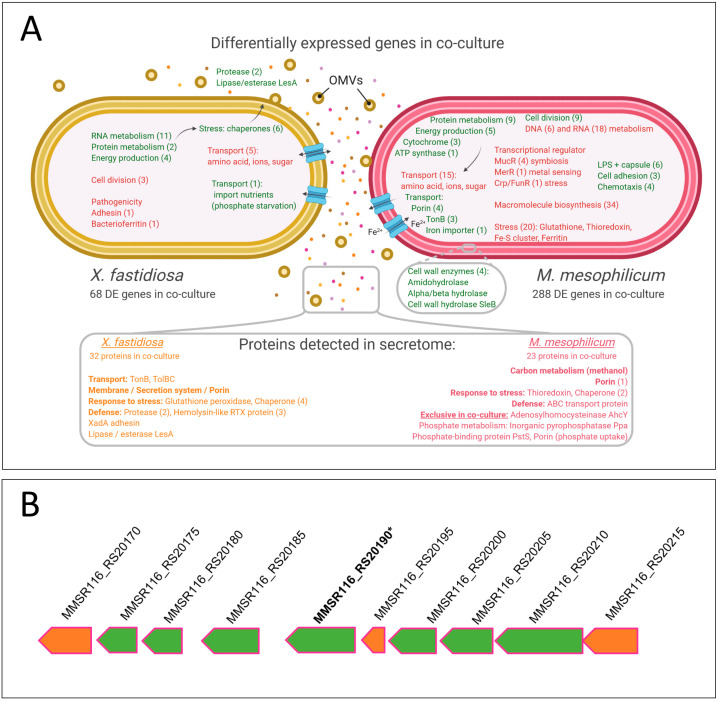
(**A**) Schematic representation of differentially expressed genes and proteins detected in the secretome of *Xf* and *Mm* during co-culture. Green represents genes up-regulated during co-culture and red those that are down-regulated during co-culture. The number of differentially expressed genes in the functional categories is indicated in brackets. The bottom box shows the schematic representation of the secretome. Secretome analysis identified four proteins in *M. mesophilicum* exclusively produced in co-culture with *X. fastidiosa*, among these, three are related to phosphorous uptake. Orange represents *Xf* proteins and pink represents *Mm* proteins detected during co-culture. The number of proteins identified in the functional categories is indicated in brackets. OMVs—Outer membrane vesicles. *Xf*: *X. fastidiosa* 9a5c; *Xf*+*Mm*: *X. fastidiosa* 9a5c + *M. mesophilicum* SR1.6/6; *Mm*: *M. mesophilicum* SR1.6/6. (**B**) *Mm* ABC transporter operon. Green arrows represent up-regulated genes in *Xf+Mm* co-culture according to transcriptome analysis, orange arrows represent genes with similar expression in all tested treatments. (*) indicates an up-regulated gene in co-culture which encoded protein was detected in the secretome of *Mm* monoculture.

Transcriptomic and growth analyses showed that *Xf* genes associated with cell division were down-regulated in co-culture, while genes related to energy generation were up-regulated. Similar results were obtained in previous work using microarrays of *Xf*-*Mm* [30]. Overall, the present work shows that genes related to metabolism (RNA, protein, and energy production) were up-regulated (Figure 7A).

On the other hand, *Xf* showed up-regulation of pathogenesis-related genes, such as oligopeptidases, proteases, and lipase/esterase (LesA). As *Xf* is under stress and starvation due to competition against *Mm*, cell wall degrading enzymes are expressed possibly to provide additional nutrient sources. Furthermore, those enzymes were shown to be secreted via OMVs, which were previously shown to be produced in stress conditions to improve bacterial spread inside the host [20,25]. Additionally, mechanisms to secrete *Xf* proteases and lipases by different systems under diverse situations to target membrane cells of the competitor *Mm* have been hypothesized in recent articles [67,68,69].

Six different chaperone genes were also up-regulated in response to *Mm* presence. An increase in stress response was also found in previous microarray analyses [30]. 

Interestingly, several *Xf* transport genes were down-regulated during co-culture suggesting reduction of sugar and nutrient intake. However, a single phosphate transport gene (XF_2141/XF9a_02027- phosphate ABC transporter) was up-regulated (Figure 7B). Upregulation of phosphate transporter has been shown to be related to import nutrients when facing phosphate starvation in *M. tuberculosis* and *M. bovis* [70]. This expression profile might represent nutrient starvation by *Xf* following interaction with *Mm*.

Genes related to cell division were up-regulated in this condition. This may occur due to an increase in *Mm* metabolism but does not necessarily result in an increase in cell number. Moreover, similar to *Xf*, genes related to metabolism and energy production were also induced in *Mm*.

*Mm* also down-regulated several transport genes upon interaction with *Xf*, particularly those related to ions, carbohydrate, and protein uptake. However, several other transport genes were up-regulated, including ABC transporter, TonB, as well as porins involved mainly in iron and phosphorus uptake. Additionally, despite the constant agitation in broth culture, four cell adhesion genes were up-regulated in *Mm*, which may be related to glass adhesion. LPS and capsule genes were also up-regulated, which may play roles in protection and adherence [71]. Four chemotaxis and four hydrolase genes related to bacterial interaction were also up-regulated. These suggest that *Mm* might be both taking nutrients from *Xf* (mainly Fe and P) and simultaneously producing enzymes to cleave the *Xf* cell wall. Twenty *Mm* stress-related genes were down-regulated, including genes related to oxidative stress, mainly glutathione, thioredoxin, and iron detoxification and storage. Antitoxin genes related to host response were also down-regulated, indicating that *Mm* may not sense the presence of *Xf* as a stressful condition. 

Analysis of the secretome data reveals that the response of phytopathogen *Xf* in co-culture shows some overlap when compared to the endophyte *Mm* in co-culture. Proteins with putative functional similarities have been identified, hinting at potential functional convergences across *Xf* and *Mm*. Notably, both organisms appear to possess porin proteins PorinO/OprP (Table 2) and Porin (Table 3) that likely serve as outer membrane channels for the translocation of molecules. Additionally, the presence of chaperone proteins, such as Chaperonin GroEL (Table 2), and Chaperonin GroES, and Chaperone protein DnaK (Table 3), suggests shared roles in assisting protein folding and maintenance. Furthermore, the occurrence of proteins labeled as “uncharacterized” in both datasets emphasizes the need for extensive characterization efforts to elucidate the precise functions of those proteins. Also, several *Xf* proteins associated with nutrient transport, including membrane proteins (OmpA, Omp1X) and porins (OprP, CirA), along with adhesins (XadA3), lipases (Lpp and LesA), and pathogenicity-related proteins (RTX), were identified, consistent with their presence in previous monoculture studies [14,17,20]. The presence of proteins related to pathogenicity and antibiotic resistance in *Xf* implies its continuous metabolic activity in the investigated treatments, allowing it to persist within its ecological niche even in the presence of potential competitors. These findings align with the RNA-Seq data, confirming that the pathogen is compatible with environments characterized by limited nutrient availability and competition with other microorganisms. The pathogen’s capacity to endure such conditions appears to involve the production of these defense effectors, even at the expense of its survival [7,72,73].

For instance, the RTX toxicity proteins, which have been reported in previous *Xf* proteomics work conducted in our group [25], have been shown to be relatively more abundant in the co-cultured *Xf* secretome (Table 2). RTX toxicity proteins belong to a class with diverse functions described in Gram-negative bacteria. Proteins of the RTX type may have bacteriocin activity, protease activity, or lipase activity, although most of them do not have a known function [73,74].

While the secretome analyses presented in this study are not directly comparable, the protein spectra identified in the co-culture secretome for both *Xf* and *Mm* strongly indicate that these proteins may be considerably more abundant than in the respective monoculture treatments. From the detected proteins in the *Xf* and *Mm* secretome, several indicate competition for nutrients and resources. Four exclusive proteins of *Mm* were detected in co-culture: Inorganic pyrophosphatase Ppa, Phosphate-binding protein PstS, Adenosylhomocysteinase AhcY, and Porin (MMSR116_18145). Inorganic pyrophosphatase Ppa and Phosphate-binding protein PstS proteins are involved with phosphate metabolism and linked to lipid metabolism; the protein Adenosylhomocysteinase AhcY participates in the synthesis of the nucleoside adenosine and homocysteine biosynthesis. Two of these proteins, Phosphate-binding protein PstS (MMSR116_RS19505) and Porin (MMSR116_18145) are membrane proteins that facilitate phosphate entry. Porin protein, also present in transcriptomes, can be involved in phosphate acquisition, nutrient uptake, bacteriocin, and phage receptors.

Apparently, *Xf* synchronizes the expression of certain proteins (e.g., OmpA and porin CirA) to allow nutrient intake from the environment and increase proteins related to catalytic reactions (Glutathione peroxidase), whereas *Mm* focuses exclusively on expressing proteins to allow nutrient intake (porins). In the GO annotations, notably, “metabolic process” emerges as the predominant one across all treatment conditions, suggesting the vital involvement of these proteins in diverse metabolic pathways. Furthermore, the “cellular process” also exhibits significant enrichment in the secretome, underscoring the importance of cellular-level activities. Intriguingly, specific GO annotations appear to be unique to each bacterial strain. In the case of *Xf*, annotations like “biological adhesion”, “protein-containing complex”, “cell part,” “cell”, “transporter activity”, “catalytic activity”, and “reproduction” are exclusively associated with its secretome (either mono or co-culture), shedding light on *Xf* specialized protein secretion in response to the treatments. Conversely, “biological regulation” is an exclusive GO annotation for *Mm*, implying its distinct roles in regulating biological processes within the co-culture environment. These findings collectively enhance our understanding of the functional roles and adaptations of *Xf* and *Mm* in various treatment conditions, contributing to the broader knowledge of their secretome characteristics and their implications in biological interactions.

It is well-established that during the later stages of bacterial growth, cell death can lead to the release of intracellular components, resulting in medium contamination with membrane components and cytosolic proteins, as evidenced in numerous previous studies [75,76,77,78,79]. However, it is important to note that bacterial cell lysis was not specifically evaluated in our experiments. Still, the presence of typical intracellular proteins in the secretome, such as elongation factors (EF-Tu), chaperones (DnaK, GroEL, and GroES), and cold-shock protein CspA, is not a novel finding. GroEL and EF-Tu are among the most abundant proteins within bacterial cells, and their occurrence in secreted proteomes could be attributed to substantial cell lysis. Remarkably, both of these proteins are recognized as moonlighting proteins, capable of performing multiple physiologically relevant biochemical or biophysical functions, which are not the result of gene fusions [79,80,81,82]. EF-Tu, for instance, has been found in the cell walls, membranes, and secretomes of several bacteria [83,84]. GroEL is believed to have an extracellular function in pathogenic processes [85,86]. 

Moreover, outer membrane vesicles (OMVs) were previously reported in *Xf* being associated with pathogenesis, such as by trafficking degradative enzymes against competing bacteria [87] and quorum sensing diffusible signaling factors [25], or by acting as an extracellular antiadhesive, blocking surface attachment and modulating plant colonization [23]. A total of 8 outer membrane proteins were detected in *Xf* monoculture, among those the XadA protein, an adhesin, which is reported to be associated with OMVs. Four outer membrane proteins were also detected in *Xf* co-culture, which included XadA. Another protein reported to be secreted in OMVs is the lipase/esterase LesA [20], but also secreted through the Type 2 Secretion System [88], which was found to be up-regulated in the *Xf* transcriptome and present in both mono and co-culture treatment secretomes. LesA is reported as a key virulence factor, we speculate that it can also potentially act on other competing bacteria, such as *Mm*. Remains to be investigated if OMVs can be used as a defense mechanism of *Xf* against *Mm* or other bacteria-sharing niches.

Moreover, our approach to analyzing common differentially expressed genes/proteins in the transcriptome and secretome analyses reveals relevant trends between up- or down-regulation from transcript to protein expression level. The GO annotation networks highlighted these trends as the investigated genes were elucidated through functional information. 

## 5. Conclusions

The present study is the first report of endophyte-phytopathogen interaction using a global approach combining physiological assays with RNA-Seq and secretome analyses. Integrating all obtained results suggests *Mm* can suppress the phytopathogen *Xf* mainly by nutrient competition within xylem vessels, and not only by an active killing mechanism.

## Figures and Tables

**Table 1:** Average number of mapped reads in *Xf* and *Mm* using CLC Genomics Workbench software. *Xf: X. fastidiosa*; *Mm*: *M. mesophilicum*.

Treatment	Number of Reads	Mapped Reads in Pairs	% Mapped Reads	Unique Fragments
*Xf* monoculture	4,400,994	3,523,819	80.16	1,759,574
Co-culture mapped in *Xf* genome	4,410,865	2,284,762	51.05	1,140,964
Co-culture mapped in *Mm* genome	4,410,865	1,158,502	26.41	578,282
*Mm* monoculture	3,981,063	2,892,542	71.45	1,444,234

**Table 2:** Proteins identified in the *Xf* secretome in monoculture (MC) or in co-culture (CC) with *Mm*. MC: Ranking of proteins in order of abundance of spectra in the monoculture, where the most abundant is number 1. CC: Ranking of proteins in order of abundance of spectra in the co-culture.

MC	CC	CDS Number IMG *Xf* *	CDS Number NCBI *Xf* *	Description and Symbol **	Predicted Molecular Mass (kDa)	Sequence Coverage (%)	SignalP	SecP Score
1	3	XF9a_01687	XF_1803	Omp1X	21	26	20|21	
2	1	XF9a_01416	XF_1547	Membrane lipoprotein Lpp	16	73	17|18	
3	2	XF9a_00315	XF_0343	Outer membrane protein mopB	42	28		0.94
4	11	XF9a_00326	XF_0363	Outer membrane protein OmpA	26	33	27|28	
5	4	XF9a_00948	XF_1026	Serine protease PspB	95	18		0.95
6	7	XF9a_01869	XF_1981	Adhesin XadA3	118	23		0.95
7	12	XF9a_00900	XF_0975	PorinO OprP	44	35	21|22	
8	6	XF9a_00602	XF_0668	Hemolysin toxin protein RTX	128	22		0.74
9	13	XF9a_00831	XF_0898	Membrane lipoprotein	13	53		
10	9	XF9a_02226	XF_2349	Autotransporter beta-domain	81	26		0.85
11	5	XF9a_01123	XF_1219	Protein of Unknown Function	9	23		
12	10	XF9a_02129	XF_2237	TonB-dependent receptor	103	21	31|32	
13	-	XF9a_01121	XF_1217	Protein of Unknown Function	9	17		
14	8	XF9a_00936	XF_1011	Hemolysin toxin protein RTX	173	5		0.57
15	14	XF9a_00892	XF_0964	Membrane lipoprotein	19	24		0.56
16	15	XF9a_01736	XF_1851	Serine protease	105	10		0.95
17	-	XF9a_01786	XF_1896	Outer membrane protein OmpA	20	10	27|28	
18	21	XF9a_00582	XF_0644	Peptidylprolyl isomerase Fkbp	25	18	19|20	
19	19	XF9a_00514	XF_0565	Lipid-binding SYLF	32	13	22|23	
20	16	XF9a_02272	XF_2407	Hemolysin toxin protein RTX	219	5		0.69
21	18	XF9a_02555	XF_2713	Porin CirA	96	15	24|25	
22	29	XF9a_02447	XF_2586	Outer membrane export factor TolC	49	23	21|22	
23	17	XF9a_00557	XF_0615	Chaperonin GroEL	58	16		
24	20	XF9a_01776	XF_1887	Cysteine/serine peptidase PS-46	79	16	22|23	
25	26	XF9a_01787	XF_1897	protein TolB	48	17		0.78
26	-	XF9a_02412	XF_2548	Succinyl-CoA ligase SucD	30	21		0.64
27	30	XF9a_00323	XF_0357	Lipase/Esterase LesA	42	15		0.90
28	-	XF9a_01186	XF_1297	Gluconolactonase	37	5	25|26	
29	23	XF9a_01725	XF_1840	Protein of Unknown Function	25	7		0.90
30	-	XF9a_01712	XF_1827	Organic hydroperoxide reductase OsmC/OhrA	15	20		
31	-	XF9a_00558	XF_0616	Chaperonin GroES	10	39		
32	-	XF9a_01475	XF_1604	Glutathione peroxidase	21	16	27|28	
33	-	XF9a_00607	XF_0672	Acyl carrier protein AcpP	9	24		
34	22	XF9a_00073	XF_0082	Fimbrial chaperone protein PapD	29	15	30|31	
35	-	XF9a_00777	XF_0855	Lipoprotein NlpD	26	8	26|27	
36	-	XF9a_00319	XF_0353	Translation initiation inhibitor	14	18		
37	25	XF9a_02217	XF_2340	Chaperone protein DnaK	68	15		
38	-	XF9a_01697	XF_1811	Outer membrane protein Slp	18	17		0.91
39	-	XF9a_01043	XF_1133	Tryptophan repressor binding protein WrbA	20	10		0.92
40	24	XF9a_01514	XF_1649	Protein of Unknown Function	30	11		
41	-	XF9a_00742	XF_0816	Zn-dependent peptidase	108	4		
42	-	XF9a_00954	XF_1036	Porin	111	9	33|34	
43	27	XF9a_00221	XF_0239	Polyribonucleotide nucleotidyltransferase Pnp	76	7		
44	28	XF9a_00502	XF_0550	TonB-dependent receptor	114	4	34|35	
45	-	XF9a_02478	XF_2622	Cold shock protein, CspA	9	21		0.89
46	-	XF9a_02496	XF_2640	Elongation factor Tu tufA	43	16		
47	-	XF9a_01153	XF_1253	Acetyl esterase/lipase	35	6		0.59
48	-	XF9a_00069	XF_0078	Fimbrial protein MrkD	37	6	37|38	
49	-	XF9a_02168	XF_2283	Beta-lactamase-like	34	8	23|24	
50	-	XF9a_01476	XF_1605	Fkbp-type peptidyl-prolyl cis-trans isomerase FkpA	32	7		0.74
51	-	XF9a_00181	XF_0196	DUF2059	20	20	26|27	
52	-	XF9a_00120	XF_0138	Aminopeptidase PepA	52	12		
53	-	XF9a_00760	XF_0838	Chaperone SurA	51	5	25|26	
54	32	XF9a_00744	XF_0820	Zn-dependent amino- or carboxypeptidase	58	9		0.52
55	-	XF9a_01399	XF_1527	Type II protein GspD/PulD	81	3		0.95
56	-	XF9a_02411	XF_2547	Succinate--CoA ligase subunit beta SucC	41	7		
57	-	XF9a_01116	XF_1211	Malate dehydrogenase Mdh	35	8		
58	-	XF9a_01785	XF_1895	Tol-pal System protein YbgF	30	8	23|24	
59	-	XF9a_00964	XF_1046	Outer membrane protein BamA	88	6	26|27	
60	-	XF9a_01449	XF_1577	DUF2184	37	6		0.53
61	31	XF9a_02619	XF_2773	Hemagglutinin HxfA	361	1		0.96
62	-	XF9a_00008	XF_0007	Protein of Unknown Function	44	6		0.75
63	-	XF9a_00264	XF_0290	Aconitase	98	3		0.78
64	-	XF9a_00072	XF_0081	Outer membrane FimD	98	2		0.91

* Number of the locus tag of *Xf* gene. ** Name assigned to gene and retrieved from UNIPROT and IMG/ER platform.

**Table 3:** Proteins identified in the secretome of *M. mesophilicum* in monoculture (IC) or in co-culture (CC) with *X. fastidiosa.* MC: Ranking of proteins in order of abundance of spectra in the monoculture, where the most abundant is number 1. CC: Ranking of proteins in order of abundance of spectra in the co-culture.

MC	CC	CDS Number Mb *	CDS Number “Old NCBI Locustag” Mb *	Description and Symbol **	Predicted Molecular Mass kDa	Sequence Coverage (%)	SignalP	SecP Score
1	-	MMSR116_RS18845	MMSR116_19085	Flagellin	41	16		0.97
2	2	MMSR116_RS23965	MMSR116_24295	Cytochrome c class I	13	15	22|23	
3	-	MMSR116_RS28515	MMSR116_28855	Uncharacterized protein	12	10	24|25	
4	3	MMSR116_RS16505	MMSR116_16705	Formaldehyde-activating enzyme	18	8		0.82
5	10	MMSR116_RS16515	MMSR116_16715	Chaperonin GroES	11	21		
6	-	MMSR116_RS22410	MMSR116_22715	Uncharacterized protein	17	19	25|26	
7	-	MMSR116_RS23130	MMSR116_23445	Peptidyl-prolyl cis-trans isomerase	19	19	22|23	
8	9	MMSR116_RS12040	MMSR116_12205	Thioredoxin Trx	12	20		0.73
9	5	MMSR116_RS00465	MMSR116_00470	Porin	29	19	20|21	
10	-	MMSR116_RS24205	MMSR116_24535	Uncharacterized protein	15	14	23|24	
11	8	MMSR116_RS12140	MMSR116_12305	Uncharacterized protein	10	12	23|24	
12	22	MMSR116_RS25265	MMSR116_25605	Elongation factor Tuf1	43	6		
13	-	MMSR116_RS13860	MMSR116_14035	Signal peptide protein	19	20	21|22	
14	6	MMSR116_RS20185	MMSR116_20450	Extracellular solute-binding protein	33	23	27|28	
15	12	MMSR116_RS27490	MMSR116_27830	Multiple sugar-binding periplasmic receptor ChvE	38	15	27|28	
16	7	MMSR116_RS07030	MMSR116_07145	Glyceraldehyde-3-phosphate dehydrogenase	36	13		
17	4	MMSR116_RS11970	MMSR116_12135	Cysteine synthase A	34	14		
18	-	MMSR116_RS17175	MMSR116_17365	Peptidase PepSY	20	21	21|22	
19	13	MMSR116_RS09940	MMSR116_10105	Chaperone protein DnaK	69	10		
20	-	MMSR116_RS16510	MMSR116_16710	Chaperone protein GroEL	58	20		
21	1	MMSR116_RS07550	MMSR116_07665	Extracellular ligand-binding receptor	39	26	20|21	
22	14	MMSR116_RS09540	MMSR116_09680	NAD-binding 6-phosphogluconate dehydrogenase	30	14		
23	-	MMSR116_RS26210	MMSR116_26565	Catalase-related peroxidase	35	8	19|20	
24	-	MMSR116_RS09370	MMSR116_09510	Superoxide dismutase	23	9		0.51
25	-	MMSR116_RS25335	MMSR116_25675	Transcription elongation factor GreA	16	13		
26	-	MMSR116_RS18790	MMSR116_19035	Uncharacterized protein	8	28	23|24	
27	-	MMSR116_RS03745	MMSR116_03785	Uncharacterized protein	23	7	23|24	
28	-	MMSR116_RS24405	MMSR116_24735	Transcriptional regulator, MucR family	18	10		0.96
29	-	MMSR116_RS12930	MMSR116_13100	Uncharacterized protein	22	9	27|28	
30	-	MMSR116_RS09955	MMSR116_10120	Redoxin	17	11		
31	-	MMSR116_RS23810	MMSR116_24135	Citrate synthase	48	3		
32	-	MMSR116_RS14870	MMSR116_15050	Uncharacterized protein	17	16	36|37	
33	-	MMSR116_RS01755	MMSR116_01770	Alanine racemase domain-containing protein	40	12		
34	15	MMSR116_RS14980	MMSR116_15160	Phosphoenolpyruvate carboxykinase PckA	59	6		
35	18	MMSR116_RS20370	MMSR116_20635	Malate dehydrogenase Mdh	34	7		
36	-	MMSR116_RS20190	MMSR116_20455	branched-chain amino acid ABC transporter substrate-binding protein	43	9	25|26	
37	20	MMSR116_RS00865	MMSR116_00880	Methanol/ethanol family PQQ-dependent dehydrogenase	69	3	24|25	
38	19	MMSR116_RS20480	MMSR116_20745	Polyribonucleotide nucleotidyltransferase Pnp	80	3		
39	23	MMSR116_RS29630	MMSR116_29970	Ketol-acid reductoisomerase IlvC	37	5		
40	-	MMSR116_RS11180	MMSR116_11330	Adenylosuccinate synthetase PurA	48	6		
41	-	MMSR116_RS21010	MMSR116_21290	Methylenetetrahydrofolate dehydrogenase	29	8		0.81
42	-	MMSR116_RS05635	MMSR116_05745	30S ribosomal protein S1 RpsA	63	3		
43	-	MMSR116_RS03750	MMSR116_03790	Aconitate hydratase	97	3		0.60
-	11	MMSR116_RS21075 ***	MMSR116_21360	Inorganic pyrophosphatase Ppa	20	8		
44	-	MMSR116_RS13495	MMSR116_13665	Pyruvate, phosphate dikinase	97	2		
-	16	MMSR116_RS19390 ***	MMSR116_19640	Adenosylhomocysteinase AhcY	51	5		
-	17	MMSR116_RS19735 ***	MMSR116_19990	Phosphate-binding protein PstS	37	6	23|24	
-	22	****	MMSR116_18145 ***	Porin	59	3		0.89

* Number of the locus tag of the bacterium *M. mesophilicum*. ** Name assigned to the gene and retrieved from the UNIPROT platform. *** Proteins only detected in the co-culture. **** There is no equivalent locustag on this genome annotation.

## Data Availability

Transcriptome sequencing (RNA-seq) data reported in this work have been deposited in the NCBI SRA database under BioSample accessions SAMN37339366, SAMN37339367, SAMN37339368, SAMN37339369, SAMN37339370, SAMN37339371, SAMN37339372, SAMN37339373 and SAMN37339374.

## Supplementary Materials

The following supporting information can be downloaded at: https://www.mdpi.com/article/10.3390/microorganisms11112755/s1, Table S1: Quantification of cDNA libraries using Nanodrop (absorbance values). Treatments: *Xf*: *X. fastidiosa* 9a5c; *Xf*+*Mm*: *X. fastidiosa* 9a5c + *M. mesophilicum* SR1.6/6; *Mm*: *M. mesophilicum* SR1.6/6; Table S2: Amount of reads per run and treatment. Treatments: *Xf*: *X. fastidiosa* 9a5c; *Xf*+*Mm*: *X. fastidiosa* 9a5c + *M. mesophilicum* SR1.6/6; *Mm*: *M. mesophilicum* SR1.6/6; Table S3: Number of mapped reads in *Xf* and *Mm* using CLC Genomics Workbench software. *Xf*: *X. fastidiosa*; *Mm*: *M. mesophilicum*; Table S4: Differentially expressed genes in the phytopathogen *Xf* only and co-culture; Table S5: Differentially expressed genes in the endophyte *Mm* only and during co-culture; Table S6: Trends in genes detected in transcriptome and secretome of *Xf*; Table S7: Trends in genes detected in transcriptome and secretome of *Mm*; Figure S1: Detailed integration of transcriptome and secretome analyses.
