# Supplementary material for: Transcriptome and Secretome Analyses of Endophyte Methylobacterium mesophilicum and Pathogen Xylella fastidiosa Interacting Show Nutrient Competition
Source: Microorganisms. 2023 Nov 11;11(11):2755. doi: 10.3390/microorganisms11112755 (PMC10673610; doi:10.3390/microorganisms11112755)
Supplement: Supplementary file 1 [file microorganisms-11-02755-s001.zip › microorganisms-2633460-supplementary.pdf]

Suplementary material

Table S1. Quantification of cDNA libraries using Nanodrop (absorbance values). Treatments: *Xf*: *X. fastidiosa* 9a5c*; Xf+Mm*: *X. fastidiosa* 9a5c + *M. mesophilicum* SR1.6/6; *Mm*: *M. mesophilicum* SR1.6/6.

	Replicate 1			Replicate 2			Replicate 3		
**Treatment**	**ng/µl**	**A260 /A280 (nm)**	**A260/ A230 (nm)**	**ng/µl**	**A260/ A280 (nm)**	**A260/ A230 (nm)**	**ng/µl**	**A260/ A280 (nm)**	**A260/ A230 (nm)**
Xf	64.7	1.89	2.23	79.1	1.88	2.29	71.4	1.86	2.19
Xf+Mm	35.1	1.89	2.20	46.5	1.87	2.20	50.9	1.87	2.11
Mm	22.6	1.90	1.98	42.7	1.85	1.96	29.6	1.89	2.13

Table S2. Amount of reads per run and per treatment. Treatments: *Xf*: *X. fastidiosa* 9a5c*; Xf+Mm*: *X. fastidiosa* 9a5c + *M. mesophilicum* SR1.6/6; *Mm*: *M. mesophilicum* SR1.6/6.

Treatment (replicate)	Index	Runs	Reads percentage	Number of reads per treatment	Total reads of the run (PF-pass filter)	Total reads >Q30* (%)
Xf (1)	AGTTCC	1	14.96	3,469,612	24,782,942	64.85
Xf+Mm (1)	CCGTCC		14.97	3,708,938		
Mm (1)	GTCCGC		13.57	3,345,697		
Xf (2)	CTTGTA	2	17.38	5,387,824	31,143,494	78.69
Xf+Mm (2)	ATGTCA		16.00	4,982,959		
Mm (2)	GTGAAA		14.67	4,546,950		
Xf (3)	AGTCAA	3	15.12	4,080,772	26,989,232	80.97
Xf+Mm (3)	ATGTCA		16.80	4,534,191		
Mm (3)	GTCCGC		14.86	4,010,600		

*Q30 score means an error probability of 1-1000 of a nucleotide assignment.

Table S3. Number of mapped reads in *Xf* and *Mm* using CLC Genomics Workbench software. *Xf: X. fastidiosa*; Mm: *M. mesophilicum.*

Treatment	Biological replicate	Number of reads	Mapped reads in pairs	% mapped reads	Unique fragments
***X. fastidiosa* monoculture**					

Xf	1	3,706,786	2,898,062	78.18	1,449,031
Xf pXF51			59,234	1.60	29,617
Xf	2	5,413,426	4,259,328	78.68	2,129,664
Xf pXF51			94,616	1.75	47,308
Xf	3	4,082,770	3,414,068	83.62	1,700,027
Xf pXF51			110,856	2.44	55,330
Average		4,400,994	3,523,819	80.16	1,759,574
**Co-culture mapped in *Xf* genome**					
Xf+Mm in *Xf*	1	3,708,938	1,514,052	40.82	757,026
Xf+Mm in *Xf* pXF51			56,182	1.51	28,091
Xf+Mm in *Xf*	2	4,987,168	2,695,846	54.06	1,347,923
Xf+Mm in *Xf* pXF51			103,718	2.08	51,859
Xf+Mm in *Xf*	3	4,536,488	2,644,388	58.29	1 317,944
Xf+Mm in *Xf* pXF51			89,750	2.20	29,417
Average		4,410,865	2,284,762	51.05	1,140,964
**Co-culture mapped in *Mm* genome**					
Xf+Mm in *Mm*	1	3,708,938	1,053,066	28.39	526,533
Xf+Mm in *Mm*	2	4,987,168	1,287,294	25.81	643,647
Xf+Mm in *Mm*	3	4,536,488	1,135,146	25.02	564,667
Average		4,410,865	1,158,502	26.41	578,282
***M. mesophilicum* monoculture**					
Mm	1	3,362,754	1,853,878	55.13	926,939
Mm	2	4,568,912	3,575,600	78.26	1,787,800
Mm	3	4,011,524	3,248,148	80.97	1,617,962
Average		3,981,063	2,892,542	71.45	1,444,234

Table S4. Differentially expressed genes in the endophyte *Xf* only and during co-culture.

Class	Genic Category		SeqName	Gene product name	log2FoldChange	Description	Length	#Hits	#GO	GO Names list	Enzyme Codes list	InterPro IDs
Macromolecule metabolism	RNA metabolism	ribosomal protein	XF9a_01063	ribosomal protein L2, bacterial/organellar	-0.429978502	RL2_XYLFA ame: Full=50S ribosomal L2	275	5	5	F:structural constituent of ribosome; C:large ribosomal subunit; F:transferase activity; F:rRNA binding; P:translation		IPR022669 (SMART); IPR002171 (SMART); IPR022669 (PFAM); IPR005880 (TIGRFAM); IPR014726 (G3DSA:4.10.950.GENE3D); IPR022666 (PFAM); IPR002171 (PIRSF); G3DSA:2.40.50.140 (GENE3D); IPR014722 (G3DSA:2.30.30.GENE3D); PTHR13691:SF5 (PANTHER); IPR002171 (PANTHER); IPR005880 (HAMAP); IPR012340 (SUPERFAMILY); IPR008991 (SUPERFAMILY)
	RNA metabolism	ribosomal protein	XF9a_02489	Ribosomal protein L10	-0.572760956	RL10_XYLFA ame: Full=50S ribosomal L10	175	5	5	F:structural constituent of ribosome; P:ribosome biogenesis; C:ribosome; F:large ribosomal subunit rRNA binding; P:translation		G3DSA:3.30.70.1730 (GENE3D); IPR001790 (PFAM); PTHR11560 (PANTHER); PTHR11560:SF8 (PANTHER); IPR022973 (HAMAP); SSF160369 (SUPERFAMILY)
	RNA metabolism	ribosomal protein	XF9a_02485	ribosomal protein S12, bacterial/organelle	-0.650397594	RS12_XYLFT ame: Full=30S ribosomal S12	132	5	5	F:structural constituent of ribosome; C:small ribosomal subunit; F:tRNA binding; F:rRNA binding; P:translation		IPR006032 (PRINTS); IPR006032 (PIRSF); IPR005679 (TIGRFAM); IPR006032 (PFAM); G3DSA:2.40.50.140 (GENE3D); PTHR11652:SF1 (PANTHER); IPR006032 (PANTHER); IPR005679 (HAMAP); IPR012340 (SUPERFAMILY)
	RNA metabolism	ribosomal protein	XF9a_02441	ribosomal protein S2, bacterial type	-0.55705189	RS2_XYLFT ame: Full=30S ribosomal S2	266	5	3	F:structural constituent of ribosome; C:small ribosomal subunit; P:translation		IPR001865 (PRINTS); G3DSA:3.40.50.10490 (GENE3D); IPR005706 (TIGRFAM); G3DSA:1.10.287.610 (GENE3D); IPR001865 (PFAM); IPR005706 (PANTHER); PTHR12534:SF0 (PANTHER); IPR005706 (HAMAP); IPR023591 (SUPERFAMILY)
	RNA metabolism	ribosomal protein	XF9a_01083	ribosomal protein S4, bacterial/organelle type	-0.588341505	RS4_XYLFA ame: Full=30S ribosomal S4	208	5	5	P:positive regulation of translational fidelity; F:structural constituent of ribosome; C:small ribosomal subunit; F:rRNA binding; P:translation		IPR002942 (SMART); IPR001912 (SMART); G3DSA:1.10.1050.10 (GENE3D); IPR002942 (PFAM); IPR036986 (G3DSA:3.10.290.GENE3D); IPR005709 (TIGRFAM); IPR001912 (PFAM); IPR022801 (PANTHER); IPR005709 (PTHR11831:PANTHER); IPR005709 (HAMAP); IPR002942 (PROSITE_PROFILES); SSF55174 (SUPERFAMILY)
	RNA metabolism	Elogation factor	XF9a_02440	translation elongation factor Ts	-0.518813753	EFTS_XYLFA ame: Full=Elongation factor Ts Short=EF-Ts	292	5	3	F:translation elongation factor activity; C:cytoplasm; P:translational elongation		IPR036402 (G3DSA:3.30.479.GENE3D); G3DSA:1.10.286.20 (GENE3D); IPR014039 (PFAM); G3DSA:1.10.8.10 (GENE3D);

												IPR001816 (TIGRFAM); IPR036402 (G3DSA:3.30.479.GENE3D); IPR001816 (PANTHER); IPR001816 (HAMAP); IPR009060 (SUPERFAMILY); IPR036402 (SUPERFAMILY); IPR036402 (SUPERFAMILY)
	RNA metabolism	Elogation factor	XF9a_02483	translation elongation factor EF-G	-0.398759307	EFG_XYLFA ame: Full=Elongation factor G Short=EF-G	705	5	5	F:GTP binding; F:translation elongation factor activity; C:cytoplasm; F:GTPase activity; P:translational elongation		IPR000795 (PRINTS); IPR000640 (SMART); IPR005517 (SMART); IPR005517 (PFAM); G3DSA:2.40.30.10 (GENE3D); G3DSA:3.30.70.240 (GENE3D); IPR005225 (TIGRFAM); IPR004161 (PFAM); IPR014721 (G3DSA:3.30.230.GENE3D); G3DSA:3.40.50.300 (GENE3D); PF14492 (PFAM); IPR000795 (PFAM); IPR000640 (PFAM); IPR004540 (TIGRFAM); G3DSA:3.30.70.870 (GENE3D); PTHR43261:SF1 (PANTHER); PTHR43261 (PANTHER); IPR000795 (PROSITE_PROFILES); IPR004540 (HAMAP); IPR027417 (SUPERFAMILY); IPR035647 (SUPERFAMILY); IPR035647 (SUPERFAMILY); IPR009000 (SUPERFAMILY); IPR020568 (SUPERFAMILY)
	RNA metabolism	Elogation factor	XF9a_02333	translation elongation factor P	-0.577403731	EFP_XYLFT ame: Full=Elongation factor P Short=EF-P	188	5	3	F:translation elongation factor activity; C:cytoplasm; P:translational elongation		IPR015365 (SMART); IPR001059 (SMART); IPR020599 (PIRSF); IPR013185 (PFAM); IPR011768 (TIGRFAM); IPR001059 (PFAM); G3DSA:2.40.50.140 (GENE3D); IPR014722 (G3DSA:2.30.30.GENE3D); IPR015365 (PFAM); G3DSA:2.40.50.140 (GENE3D); IPR020599 (PANTHER); IPR011768 (HAMAP); IPR008991 (SUPERFAMILY); IPR012340 (SUPERFAMILY); IPR012340 (SUPERFAMILY)
	RNA metabolism	Elogation factor	XF9a_02099	elongation factor P-like protein YeiP	0.854980632	EFPL_XYLFA ame: Full=Elongation factor P	188	5	3	F:translation elongation factor activity; C:cytoplasm; P:translational elongation		IPR015365 (SMART); IPR001059 (SMART); G3DSA:2.40.50.140 (GENE3D); IPR015365 (PFAM); IPR013185 (PFAM); IPR014722 (G3DSA:2.30.30.GENE3D); IPR020599 (PIRSF); IPR011897 (TIGRFAM); IPR001059 (PFAM); G3DSA:2.40.50.140 (GENE3D); PTHR30053:SF5 (PANTHER); IPR020599 (PANTHER); IPR011897 (HAMAP); IPR008991 (SUPERFAMILY); IPR012340 (SUPERFAMILY); IPR012340 (SUPERFAMILY)

	RNA metabolism		XF9a_02511	Predicted N6-adenine-specific DNA methylase (EC:2.1.1.173)	0.526446529	RLMKL_XYLFA ame: Full=Ribosomal RNA large subunit methyltransferase K L Includes: ame: Full=23S rRNA m2G2445 methyltransferase ame: Full=rRNA (guanine-N(2)-)-methyltransferase Includes: ame: Full=23S rRNA m7G2069 methyltransferase ame: Full=rRNA (guanine-N(7)-)-methyltransferase	748	5	5	F:RNA binding; C:cytoplasm; F:rRNA (guanine-N7-)-methyltransferase activity; F:23S rRNA (guanine(2445)-N(2))-methyltransferase activity; P:rRNA (guanine-N7)-methylation	EC:2.1.1.173	IPR004114 (SMART); IPR017244 (PIRSF); G3DSA:3.30.750.80 (GENE3D); G3DSA:3.30.2130.30 (GENE3D); IPR004114 (PFAM); G3DSA:3.40.50.150 (GENE3D); G3DSA:3.40.50.150 (GENE3D); IPR000241 (PFAM); IPR019614 (PFAM); PTHR18895 (PANTHER); PTHR18895:SF62 (PANTHER); IPR004114 (PROSITE_PROFILES); IPR017244 (HAMAP); IPR029063 (SUPERFAMILY); IPR029063 (SUPERFAMILY)
	RNA metabolism	transcription factor binding	XF9a_02233	Response regulator containing CheY-like receiver, AAA-type ATPase, and DNA-binding domains	0.633263148	Y2439_ANADF ame: Full=Response regulator receiver Anae109_2439	236	5	3	P:regulation of cellular process; F:binding; P:cellular process		IPR001789 (SMART); IPR008207 (PFAM); IPR036641 (G3DSA:1.20.120.GENE3D); G3DSA:3.40.50.2300 (GENE3D); IPR001789 (PFAM); PTHR26402 (PANTHER); IPR001789 (PROSITE_PROFILES); IPR008207 (PROSITE_PROFILES); IPR011006 (SUPERFAMILY); IPR036641 (SUPERFAMILY)
	RNA metabolism		XF9a_02299	integration host factor, beta subunit	-0.625635812	IHFB_XYLFA ame: Full=Integration host factor subunit beta Short=IHF-beta	104	5	7	P:regulation of translation; F:DNA binding; P:transcription, DNA-templated; P:DNA recombination; C:cytosol; P:regulation of transcription, DNA-templated; C:chromosome		IPR000119 (PRINTS); IPR000119 (SMART); IPR000119 (PFAM); IPR010992 (G3DSA:4.10.520.GENE3D); IPR005685 (TIGRFAM); IPR000119 (PANTHER); PTHR33175:SF5 (PANTHER); IPR005685 (HAMAP); IPR010992 (SUPERFAMILY)
	RNA metabolism		XF9a_01462	Predicted nucleic acid-binding protein, contains PIN domain	-0.629265663	Y4JK_RHISN ame: Full= ribonuclease Y4jK Short=RNase Y4jK ame: Full= plasmid stability Y4jK ame: Full=Toxin Y4jK	142	5	5	P:nucleic acid phosphodiester bond hydrolysis; F:protein binding; F:metal ion binding; P:migration in host; F:nuclease activity		IPR002716 (PFAM); G3DSA:3.40.50.1010 (GENE3D); PTHR33653:SF1 (PANTHER); PTHR33653 (PANTHER); IPR022907 (HAMAP); IPR029060 (SUPERFAMILY)
	RNA metabolism	post transcriptional regulation	XF9a_01626	hypothetical protein	-0.605930558	TDRD7_MOUSE ame: Full=Tudor domain-containing 7 ame: Full=PCTAIRE2-binding ame: Full=Tudor repeat associator with PCTAIRE-2 Short=Trap	208	2	17	P:germ cell development; C:ribonucleoprotein granule; C:chromatoid body; P:lens morphogenesis in camera-type eye; P:posttranscriptional regulation of gene expression; F:protein binding; F:RNA binding; C:intracellular ribonucleoprotein complex; C:cytoplasm; C:mitochondrial matrix; F:protein N-terminus binding; C:P granule; P:cell differentiation; F:mRNA binding; P:lens fiber cell differentiation		no IPS match
	RNA metabolism	miscRNA	XF9a_01899	6S / SsrS RNA (miscRNA) (XF9a_gi57014152.1: 2679305bp)	1.0111716		186					no IPS match
	RNA metabolism	miscRNA	XF9a_00950	Bacterial signal recognition particle RNA (miscRNA) (XF9a_gi57014152.1: 2679305bp)	0.809822873		100					no IPS match

	RNA metabolism	miscRNA	XF9a_00973	Bacterial RNase P class A (miscRNA) (XF9a_gi57014152.1: 2679305bp)	2.329311093		350					no IPS match
	Protein metabolism	tRNA	XF9a_00214	tRNA_Met_CAT (tRNA) (XF9a_gi57014152.1: 2679305bp)	-0.755430102		77					no IPS match
	Protein metabolism	tRNA	XF9a_02429	asparaginyl-tRNA synthetase (EC:6.1.1.22)	-0.481090758	SYN_XYLFA ame: Full=Asparagine--tRNA ligase ame: Full=Asparaginyl-tRNA synthetase Short=	466	5	5	F:nucleic acid binding; F:ATP binding; C:cytoplasm; F:asparagine-tRNA ligase activity; P:asparaginyl-tRNA aminoacylation	EC:6.1.1.22	IPR002312 (PRINTS); G3DSA:3.30.930.10 (GENE3D); G3DSA:2.40.50.140 (GENE3D); IPR004364 (PFAM); IPR004522 (TIGRFAM); IPR004365 (PFAM); PTHR22594 (PANTHER); PTHR22594:SF44 (PANTHER); IPR006195 (PROSITE_PROFILES); IPR004522 (HAMAP); SSF55681 (SUPERFAMILY); IPR012340 (SUPERFAMILY)
	Protein metabolism	tRNA	XF9a_00387	tryptophanyl-tRNA synthetase (EC:6.1.1.2)	0.677162266	SYW_XYLFA ame: Full=Tryptophan--tRNA ligase ame: Full=Tryptophanyl-tRNA synthetase Short=	430	5	4	F:ATP binding; C:cytoplasm; P:tryptophanyl-tRNA aminoacylation; F:tryptophan-tRNA ligase activity	EC:6.1.1.2	IPR002306 (PRINTS); G3DSA:1.10.240.10 (GENE3D); IPR014729 (G3DSA:3.40.50.GENE3D); IPR002306 (TIGRFAM); IPR002305 (PFAM); PTHR43766 (PANTHER); IPR024109 (HAMAP); IPR036913 (SUPERFAMILY); SSF52374 (SUPERFAMILY)
	Protein metabolism	tRNA	XF9a_02509	glutamyl-tRNA reductase (EC:1.2.1.70)	0.528218218	HEM1_XYLFA ame: Full=Glutamyl-tRNA reductase Short=	418	5	4	F:NADP binding; F:glutamyl-tRNA reductase activity; P:oxidation-reduction process; P:protoporphyrinogen IX biosynthetic process	EC:1.2.1.70	IPR015895 (PFAM); IPR000343 (TIGRFAM); IPR006151 (PFAM); IPR000343 (PIRSF); G3DSA:3.40.50.720 (GENE3D); IPR015896 (PFAM); IPR036343 (G3DSA:3.30.460.GENE3D); PTHR43013 (PANTHER); IPR000343 (HAMAP); IPR036453 (SUPERFAMILY); IPR036343 (SUPERFAMILY); IPR036291 (SUPERFAMILY)
				presente no biofilme - trabalho de proteoma								
Intermediary metabolism	Energy Production	electron transport	XF9a_01056	ATP synthase, F0 subunit c (EC:3.6.3.14)	-0.476177408	ATPL_VEREI ame: Full=ATP synthase subunit c ame: Full=ATP synthase F(0) sector subunit c ame: Full=F-type ATPase subunit c Short=F-ATPase subunit c ame: Full=Lipid-binding	101	5	6	P:ATP hydrolysis coupled proton transport; C:plasma membrane proton-transporting ATP synthase complex, coupling factor F(o); C:integral component of membrane; F:lipid binding; P:ATP synthesis coupled proton transport; F:proton-transporting ATP synthase activity, rotational mechanism		IPR000454 (PRINTS); IPR005953 (TIGRFAM); IPR038662 (G3DSA:1.20.20.GENE3D); IPR002379 (PFAM); IPR000454 (PANTHER); PTHR10031:SF0 (PANTHER); IPR000454 (HAMAP); IPR035921 (SUPERFAMILY)
		Sulfer Metabolism / ATP byossintesis	XF9a_01373	sulfate adenylyltransferase, small subunit (EC:2.7.7.4) 60:60	-0.906128907	CYSD_XYLFA ame: Full=Sulfate adenylyltransferase subunit 2 ame: Full=ATP-sulfurylase	304	5	5		EC:2.7.7.4	IPR011784 (PIRSF); IPR014729 (G3DSA:3.40.50.GENE3D); IPR002500 (PFAM); IPR011784 (TIGRFAM); PTHR43196 (PANTHER); IPR011784

						small subunit ame: Full=Sulfate adenylate transferase Short=SAT						(HAMAP); SSF52402 (SUPERFAMILY)
		Sulfer Metabolism	XF9a_01374	sulfate adenylyltransferase, large subunit (EC:2.7.1.25,EC:2.7.7.4)	-0.444791682	CYSNC_XYLFA ame: Full=Bifunctional enzyme Includes: ame: Full=Sulfate adenylyltransferase subunit 1 ame: Full=ATP-sulfurylase large subunit ame: Full=Sulfate adenylate transferase Short=SAT Includes: ame: Full=Adenylyl-sulfate kinase ame: Full=APS kinase ame: Full=ATP adenosine-5 -phosphosulfate 3 -phosphotransferase	660	5	8	F:GTP binding; F:ATP binding; P:hydrogen sulfide biosynthetic process; F:GTPase activity; F:adenylylsulfate kinase activity; F:sulfate adenylyltransferase (ATP) activity; P:phosphorylation; P:sulfate assimilation	EC:2.7.7.4; EC:2.7.1.25	IPR000795 (PRINTS); G3DSA:3.40.50.300 (GENE3D); IPR011779 (TIGRFAM); PF01583 (PFAM); G3DSA:2.40.30.10 (GENE3D); IPR002891 (TIGRFAM); IPR000795 (PFAM); G3DSA:2.40.30.10 (GENE3D); G3DSA:3.40.50.300 (GENE3D); PTHR11055:SF27 (PANTHER); PTHR11055 (PANTHER); IPR011779 (HAMAP); IPR002891 (HAMAP); IPR000795 (PROSITE_PROFILES); IPR027417 (SUPERFAMILY); IPR009001 (SUPERFAMILY); IPR009000 (SUPERFAMILY); IPR027417 (SUPERFAMILY)
		Faz NADPH	XF9a_01709	ketol-acid reductoisomerase (EC:1.1.1.86)	-0.483212318	ILVC_XYLFA ame: Full=Ketol-acid reductoisomerase (NADP(+)) Short=KARI ame: Full=Acetohydroxy-acid isomeroreductase Short=AHIR ame: Full=Alpha-keto-beta-hydroxylacyl reductoisomerase ame: Full=Ketol-acid reductoisomerase type 1 ame: Full=Ketol-acid reductoisomerase type I	335	5	4	P:isoleucine biosynthetic process; P:valine biosynthetic process; P:oxidation-reduction process; F:ketol-acid reductoisomerase activity	EC:1.1.1.86	IPR013116 (PFAM); IPR014359 (PIRSF); G3DSA:1.10.3730.40 (GENE3D); G3DSA:3.40.50.720 (GENE3D); IPR000506 (PFAM); IPR013023 (TIGRFAM); PTHR21371:SF1 (PANTHER); IPR013023 (PANTHER); IPR000506 (PROSITE_PROFILES); IPR013116 (PROSITE_PROFILES); IPR013023 (HAMAP); IPR036291 (SUPERFAMILY); IPR008927 (SUPERFAMILY)
		Faz NADPH	XF9a_02062	nicotinate (nicotinamide) nucleotide adenylyltransferase (EC:2.7.7.18)	0.608755034	NADD_XYLFA ame: Full=Probable nicotinate-nucleotide adenylyltransferase ame: Full=Deamido-NAD(+) diphosphorylase ame: Full=Deamido-NAD(+) pyrophosphorylase ame: Full=Nicotinate mononucleotide adenylyltransferase Short= adenylyltransferase	222	5	3	F:ATP binding; F:nicotinate-nucleotide adenylyltransferase activity; P:NAD biosynthetic process	EC:2.7.7.18	IPR004821 (TIGRFAM); IPR004821 (PFAM); IPR005248 (TIGRFAM); IPR014729 (G3DSA:3.40.50.GENE3D); PTHR21342 (PANTHER); IPR005248 (HAMAP); SSF52374 (SUPERFAMILY)
		electron transport	XF9a_00841	ubiquinol-cytochrome c reductase, iron-sulfur subunit (EC:1.10.2.2)	1.012664941	UCRI_ALLVD ame: Full=Ubiquinol-cytochrome c reductase iron-sulfur subunit ame: Full=Rieske iron-sulfur Short=RISP	210	5	7	F:oxidoreductase activity; C:intracellular membrane-bounded organelle; P:metabolic process; C:cytoplasmic part; C:membrane part; F:binding; P:single-organism process		IPR005805 (PRINTS); IPR036922 (G3DSA:2.102.10.GENE3D); IPR006317 (TIGRFAM); IPR019470 (PFAM); IPR017941 (PFAM); PTHR10134:SF1 (PANTHER); IPR014349 (PANTHER); IPR017941 (PROSITE_PROFILES); IPR036922 (SUPERFAMILY)

		citric acid cycle	XF9a_01739	hydro-lyases, Fe-S type, tartrate/fumarate subfamily, alpha region/hydro-lyases, Fe-S type, tartrate/fumarate subfamily, beta region (EC:4.2.1.2)	0.48574495	FUMB_ECOLI ame: Full=Fumarate hydratase class anaerobic ame: Full=D-tartrate dehydratase ame: Full=Fumarase B	530	5	3	F:hydro-lyase activity; F:binding; P:cellular process		IPR011167 (PIRSF); IPR004646 (TIGRFAM); IPR004647 (PFAM); IPR004646 (PFAM); IPR036660 (G3DSA:3.20.130.GENE3D); IPR004647 (TIGRFAM); PTHR30389 (PANTHER); IPR036660 (SUPERFAMILY)
Cell division			XF9a_02226	Predicted integral membrane proteins containing uncharacterized repeats	0.558717254	---NA---	732					IPR005546 (SMART); G3DSA:2.40.128.130 (GENE3D); IPR005546 (PROSITE_PROFILES); IPR036709 (SUPERFAMILY)
			XF9a_00732	Cell division septal protein	1.009145116	FTSQ_XANCP ame: Full=Cell division	278	5	4	P:cell division; C:integral component of membrane; C:plasma membrane; P:cell cycle		G3DSA:3.10.20.310 (GENE3D); IPR013685 (PFAM); IPR005548 (PFAM); G3DSA:3.40.50.11690 (GENE3D); IPR026579 (PANTHER); IPR026579 (HAMAP); IPR034746 (PROSITE_PROFILES)
			XF9a_01036	MAF protein	0.763840866	Y1124_XYLFA ame: Full=Maf XF_1124	199	5	1	C:cytoplasm		IPR003697 (TIGRFAM); IPR029001 (G3DSA:3.90.950.GENE3D); IPR003697 (PFAM); IPR003697 (PIRSF); IPR003697 (PANTHER); PTHR43213:SF7 (PANTHER); IPR003697 (HAMAP); IPR029001 (SUPERFAMILY)
Transport	The accumulation of this protein is enhanced under phosphate starvation	import nutrientes (phosphate starvation)	XF9a_02027	phosphate ABC transporter, phosphate-binding protein	-0.50833056	PSTS_XYLFA ame: Full=Phosphate-binding Short=PBP Flags: Precursor	364	5	6	C:periplasmic space; F:phosphate ion binding; P:cellular response to phosphate starvation; F:ATPase-coupled phosphate ion transmembrane transporter activity; C:ATP-binding cassette (ABC) transporter complex; P:phosphate ion transmembrane transport	EC:3.6.3.27	IPR005673 (PIRSF); G3DSA:3.40.190.10 (GENE3D); IPR005673 (TIGRFAM); IPR024370 (PFAM); G3DSA:3.40.190.10 (GENE3D); PTHR42996 (PANTHER); PTHR42996:SF2 (PANTHER); PS51257 (PROSITE_PROFILES); SSF53850 (SUPERFAMILY)
		transport ions	XF9a_00833	Mg2+ and Co2+ transporters	0.662012976	CORA_THEMA ame: Full=Cobalt magnesium transport	337	5	8	F:magnesium ion binding; P:cobalt ion transport; C:integral component of plasma membrane; F:cobalt ion transmembrane transporter activity; P:protein homooligomerization; F:magnesium ion transmembrane transporter activity; P:magnesium ion transmembrane transport; F:cobalt ion binding		G3DSA:1.20.58.340 (GENE3D); G3DSA:1.20.58.340 (GENE3D); G3DSA:3.30.460.20 (GENE3D); IPR002523 (PFAM); PTHR21535 (PANTHER); PTHR21535:SF33 (PANTHER); SSF143865 (SUPERFAMILY); SSF144083 (SUPERFAMILY)
		transport sulphate, thiosulphate,	XF9a_01231	sulfate ABC transporter, permease protein CysW	0.480031167	CYSW_SYNE7 ame: Full=Sulfate transport system permease	319	5	5	P:sulfate transport; P:sulfate assimilation; C:plasma membrane;		IPR000515 (PFAM); IPR011866 (TIGRFAM); IPR035906 (G3DSA:1.10.3720.GENE3D);

		selenate, and selenite.								C:plastid; F:sulfate transmembrane transporter activity		IPR005667 (TIGRFAM); IPR011866 (PTHR30406:PANTHER); IPR005667 (PANTHER); IPR000515 (PROSITE_PROFILES); IPR035906 (SUPERFAMILY)
			XF9a_00121	Predicted permeases	0.588757018	LPTF_HAEIN ame: Full=Lipopolysaccharide export system permease	361	4	17	P:transmembrane transport; P:deoxyribonucleoside monophosphate biosynthetic process; P:transport; F:thymidine kinase activity; C:plasma membrane; F:nucleotide binding; F:ATP binding; F:metal ion binding; C:membrane; C:integral component of membrane; P:DNA biosynthetic process; P:phosphorylation; F:transferase activity; F:kinase activity; F:lipopolysaccharide-transporting ATPase activity; C:ATP-binding cassette (ABC) transporter complex; P:lipopolysaccharide transport		IPR030922 (TIGRFAM); IPR005495 (PFAM); IPR005495 (PANTHER); PTHR33529:SF6 (PANTHER)
		small peptides, secondary amino acids bound to metals, and glutathione	XF9a_02149	putative oligopeptide transporter, OPT family	0.466887786	Y561_HAEIN ame: Full= oligopeptide transporter HI_0561	653	4	1	P:transport		IPR004814 (TIGRFAM); IPR004813 (PFAM); IPR004813 (TIGRFAM); PTHR31645 (PANTHER); PTHR31645:SF0 (PANTHER)
		transport sugars, amino acids, organo cations	XF9a_02141	transporter, SSS family	0.601176148	ACTP_PECCP ame: Full=Cation acetate symporter ame: Full=Acetate permease ame: Full=Acetate transporter	577	5	6	F:tellurite uptake transmembrane transporter activity; F:glycolate transmembrane transporter activity; C:integral component of plasma membrane; P:plasma membrane acetate transport; F:acetate transmembrane transporter activity; P:tellurite transport		IPR001734 (TIGRFAM); IPR038377 (G3DSA:1.20.1730.GENE3D); IPR001734 (PFAM); PTHR43291 (PANTHER); PTHR43291:SF2 (PANTHER); IPR001734 (PROSITE_PROFILES)
Response to stress		ascorbate metabolism	XF9a_02311	Uncharacterized protein conserved in bacteria	0.461290917	DLGD_HAEIG ame: Full=2,3-diketo-L-gulonate reductase Short=2,3-DKG reductase ame: Full=3-dehydro-L-gulonate 2-dehydrogenase	533	2	7	F:oxidoreductase activity, acting on the CH-OH group of donors, NAD or NADP as acceptor; C:cytoplasm; F:oxidoreductase activity; P:metabolic process; P:oxidation-reduction process; F:NAD+ binding; F:3-dehydro-L-gulonate 2-dehydrogenase activity		IPR016883 (PIRSF); G3DSA:1.50.10.140 (GENE3D); IPR019282 (PFAM); PS51257 (PROSITE_PROFILES)
			XF9a_02312	Stress-induced morphogen (activity unknown)	0.814400062	SUFE1_ARATH ame: Full= chloroplastic mitochondrial ame: Full=Chloroplastic Short= ufE ame: Full= EMBRYO DEFECTIVE 1374 ame: Full= SULFUR E Short= ame: Full= SULFUR E 1 Short= 1 Flags: Precursor	96	5	7	F:protein binding; P:transcription, DNA-templated; C:mitochondrion; C:cytosol; F:enzyme activator activity; P:iron-sulfur cluster assembly		G3DSA:3.30.300.90 (GENE3D); IPR002634 (PIRSF); IPR002634 (PFAM); IPR002634 (PANTHER); PTHR12735:SF23 (PANTHER); IPR036065 (SUPERFAMILY)
	virulence factor		XF9a_01278	lactoylglutathione lyase (EC:4.4.1.5)	0.733008607	LGUL_PSEPU ame: Full=Lactoylglutathione lyase ame: Full=Aldoketomutase ame: Full=Glyoxalase I	163	5	9	P:osteoclast differentiation; P:carbohydrate metabolic process; F:zinc ion binding; C:extracellular exosome; P:glutathione metabolic	EC:4.4.1.5	IPR029068 (G3DSA:3.10.180.GENE3D); IPR004361 (TIGRFAM); IPR004360 (PFAM); PTHR10374:SF16 (PANTHER);

						Short=Glx I ame: Full=Ketone-aldehyde mutase ame: Full=Methylglyoxalase ame: Full=S-D-lactoylglutathione methylglyoxal lyase				process; P:methylglyoxal catabolic process to D-lactate via S-lactoyl-glutathione; P:negative regulation of apoptotic process; F:lactoylglutathione lyase activity; P:regulation of transcription from RNA polymerase II promoter		PTHR10374 (PANTHER); IPR037523 (PROSITE_PROFILES); IPR029068 (SUPERFAMILY)
	chaperone		XF9a_00903	Molecular chaperone, HSP90 family	-0.438068132	HTPG_XYLFA ame: Full=Chaperone ame: Full=Heat shock ame: Full=High temperature G	637	5	5	F:ATP binding; C:cytoplasm; F:unfolded protein binding; P:protein folding; P:response to stress		IPR020575 (PRINTS); IPR003594 (SMART); G3DSA:3.40.50.11260 (GENE3D); G3DSA:3.30.230.80 (GENE3D); IPR003594 (PFAM); IPR037196 (G3DSA:1.20.120.GENE3D); IPR001404 (PIRSF); IPR001404 (PFAM); IPR036890 (G3DSA:3.30.565.GENE3D); IPR001404 (PANTHER); PTHR11528:SF54 (PANTHER); IPR001404 (HAMAP); IPR037196 (SUPERFAMILY); IPR036890 (SUPERFAMILY); IPR020568 (SUPERFAMILY)
	chaperone		XF9a_00557	chaperonin GroL	-0.783203974	CH60_XYLFT ame: Full=60 kDa chaperonin ame: Full= ame: Full= Cpn60	547	5	4	F:ATP binding; C:cytoplasm; F:unfolded protein binding; P:protein refolding		IPR001844 (PRINTS); IPR027413 (G3DSA:1.10.560.GENE3D); IPR002423 (PFAM); IPR027409 (G3DSA:3.50.7.GENE3D); IPR027410 (G3DSA:3.30.260.GENE3D); IPR001844 (TIGRFAM); PTHR11353:SF169 (PANTHER); PTHR11353 (PANTHER); IPR001844 (HAMAP); IPR027413 (SUPERFAMILY); IPR027409 (SUPERFAMILY); IPR027410 (SUPERFAMILY)
	chaperone		XF9a_00558	Co-chaperonin GroES (HSP10)	-0.679042378	CH10_XYLFT ame: Full=10 kDa chaperonin ame: Full= ame: Full= Cpn10	95	5	7	F:ATP binding; F:metal ion binding; F:chaperone binding; P:chaperone mediated protein folding requiring cofactor; F:unfolded protein binding; P:response to unfolded protein; C:GroEL-GroES complex		IPR020818 (PRINTS); IPR020818 (SMART); IPR037124 (G3DSA:2.30.33.GENE3D); IPR020818 (PFAM); PTHR10772:SF24 (PANTHER); IPR020818 (PANTHER); IPR020818 (HAMAP); IPR011032 (SUPERFAMILY)
	chaperone		XF9a_02217	chaperone protein DnaK	-0.691999608	DNAK_XYLFA ame: Full=Chaperone ame: Full=HSP70 ame: Full=Heat shock 70 kDa ame: Full=Heat shock 70	638	5	3	F:ATP binding; F:unfolded protein binding; P:protein folding		IPR013126 (PRINTS); IPR029047 (G3DSA:2.60.34.GENE3D); G3DSA:3.90.640.10 (GENE3D); IPR029048 (G3DSA:1.20.1270.GENE3D); IPR013126 (PFAM); IPR012725 (TIGRFAM); G3DSA:3.30.420.40 (GENE3D); G3DSA:3.30.420.40 (GENE3D); IPR013126 (PANTHER); PTHR19375:SF333 (PANTHER); IPR012725 (HAMAP); SSF53067 (SUPERFAMILY); IPR029047

												(SUPERFAMILY); SSF53067 (SUPERFAMILY)
	chaperone		XF9a_02218	Molecular chaperone GrpE (heat shock protein)	-0.674577014	GRPE_XYLFA ame: Full= ame: Full=HSP-70 cofactor	172	5	6	F:protein homodimerization activity; C:cytoplasm; F:chaperone binding; P:regulation of catalytic activity; P:protein folding; F:adenyl-nucleotide exchange factor activity		IPR000740 (PRINTS); IPR000740 (PFAM); IPR009012 (G3DSA:2.30.22.GENE3D); IPR013805 (G3DSA:3.90.20.GENE3D); IPR000740 (PANTHER); IPR000740 (HAMAP); IPR009012 (SUPERFAMILY); IPR013805 (SUPERFAMILY)
	chaperone		XF9a_02481	Zn-dependent protease with chaperone function (EC:3.4.24.-)	-0.57418559	HTPX_XYLFA ame: Full=Protease ame: Full=Heat shock	289	5	5	F:zinc ion binding; P:proteolysis; C:integral component of membrane; F:metalloendopeptidase activity; C:plasma membrane	EC:3.4.24	G3DSA:3.30.2010.10 (GENE3D); IPR001915 (PFAM); PTHR43221 (PANTHER); PTHR43221:SF1 (PANTHER); IPR022919 (HAMAP)
Pathogenicity, virulence, and adaptation toxin production and detoxification		biofilm formation/infecção host	XF9a_01401	Autotransporter adhesin	0.726259226	SADA_SALTY ame: Full=Autotransporter adhesin ame: Full=Salmonella adhesin A Flags: Precursor	2059	4	8	C:membrane; C:integral component of membrane; P:cell adhesion; C:cell outer membrane; C:extracellular region; P:pathogenesis; C:outer membrane; C:cell surface		G3DSA:3.30.1300.30 (GENE3D); IPR008640 (PFAM); IPR008635 (PFAM); G3DSA:3.30.390.90 (GENE3D); IPR011049 (G3DSA:2.150.10.GENE3D); G3DSA:2.20.25.490 (GENE3D); IPR011049 (G3DSA:2.150.10.GENE3D); IPR011049 (G3DSA:2.150.10.GENE3D); IPR005594 (PFAM); G3DSA:1.20.5.170 (GENE3D); PTHR35843 (PANTHER); SSF101967 (SUPERFAMILY); SSF101967 (SUPERFAMILY); SSF101967 (SUPERFAMILY); SSF101967 (SUPERFAMILY); SSF101967 (SUPERFAMILY); SSF101967 (SUPERFAMILY); SSF101967 (SUPERFAMILY); SSF101967 (SUPERFAMILY); SSF101967 (SUPERFAMILY); SSF101967 (SUPERFAMILY); SSF101967 (SUPERFAMILY); SSF54523 (SUPERFAMILY); SSF101967 (SUPERFAMILY); SSF101967 (SUPERFAMILY); SSF101967 (SUPERFAMILY); SSF101967 (SUPERFAMILY)
		peptidoglycan binding	XF9a_02068	rare lipoprotein A	0.568546895	RLPA_VIBCH ame: Full=Endolytic peptidoglycan transglycosylase Flags: Precursor	415	5	5	F:peptidoglycan binding; P:biological_process; C:membrane; F:molecular_function; C:plasma membrane		IPR007730 (PFAM); IPR036908 (G3DSA:2.40.40.GENE3D); IPR009009 (PFAM); IPR012997 (TIGRFAM); PTHR34183 (PANTHER); PTHR34183:SF1 (PANTHER); IPR034718 (HAMAP); IPR036680 (SUPERFAMILY); IPR036908 (SUPERFAMILY)

		lysozyme activity	XF9a_00840	Soluble lytic murein transglycosylase and related regulatory proteins (some contain LysM/invasin domains)	0.582319236	YJBJ_BACSU ame: Full= murein lytic transglycosylase	297	5	6	P:entry into host via enzymatic degradation of host anatomical structure; P:catabolism by organism of cell wall peptidoglycan in other organism; F:lyase activity; C:virion membrane; P:cellular process; F:lysozyme activity	EC:3.2.1.17	G3DSA:1.10.530.10 (GENE3D); IPR008258 (PFAM); PTHR45529 (PANTHER); IPR023346 (SUPERFAMILY)
	bacterial pathogeneis	lipase activity	XF9a_00323	Esterase/lipase	-0.530879896	LIPA_PSEA5 ame: Full=Lipase A Short= Flags: Precursor	422	1	5	P:lipid catabolic process; P:lipid metabolic process; F:triglyceride lipase activity; C:extracellular region; F:hydrolase activity		IPR029058 (G3DSA:3.40.50.GENE3D); IPR005152 (PIRSF); IPR005152 (PFAM); IPR029058 (SUPERFAMILY)
		protease	XF9a_00171	Membrane protease subunits, stomatin/prohibitin homologs	-0.509672157	QMCA_ECOLI ame: Full=	318	5	21	P:positive regulation of cardiolipin metabolic process; P:positive regulation of mitochondrial DNA replication; C:T cell receptor complex; F:receptor binding; F:cardiolipin binding; C:mitochondrial inner membrane; P:mitochondrial calcium ion transport; P:mitochondrion organization; P:mitochondrial ATP synthesis coupled proton transport; C:immunological synapse; P:cellular calcium ion homeostasis; C:mitochondrial intermembrane space; P:positive regulation of mitochondrial membrane potential; F:GTPase binding; C:membrane raft; C:actin cytoskeleton; C:integral component of membrane; C:COP9 signalosome; C:extrinsic component of plasma membrane; P:T cell receptor signaling pathway; P:protein oligomerization		IPR001972 (PRINTS); IPR001107 (SMART); G3DSA:3.30.479.30 (GENE3D); IPR001107 (PFAM); IPR032435 (PFAM); PTHR43327 (PANTHER); PTHR43327:SF15 (PANTHER); IPR036013 (SUPERFAMILY)
		Peptidase	XF9a_01835	Zn-dependent oligopeptidases (EC:3.4.15.5)	-0.61550075	DCP_ECOLI ame: Full=Dipeptidyl carboxypeptidase ame: Full=Peptidyl-dipeptidase Dcp	723	5	9	F:carboxypeptidase activity; P:cellular macromolecule metabolic process; P:peptide metabolic process; P:proteolysis; C:cytosol; C:outer membrane-bounded periplasmic space; C:intracellular organelle part; F:metalloendopeptidase activity	EC:3.4.24	IPR001567 (PFAM); G3DSA:1.10.1370.40 (GENE3D); IPR024077 (G3DSA:1.10.1370.GENE3D); PTHR43660 (PANTHER); SSF55486 (SUPERFAMILY)
	Nutrient assimilation	Iron storage proteins	XF9a_00357	bacterioferritin	0.564105152	BFR_ECOL6 ame: Full=Bacterioferritin Short=BFR ame: Full=Cytochrome b-1 ame: Full=Cytochrome b-557	154	5	7	F:identical protein binding; F:iron ion binding; C:cytosol; P:intracellular sequestering of iron ion; F:ferroxidase activity; C:plasma membrane; F:heme binding	EC:1.16.3.1	IPR002024 (PRINTS); IPR002024 (TIGRFAM); IPR002024 (PIRSF); IPR008331 (PFAM); IPR012347 (G3DSA:1.20.1260.GENE3D); PTHR30295:SF0 (PANTHER); PTHR30295 (PANTHER); IPR009040 (PROSITE_PROFILES); IPR009078 (SUPERFAMILY)
		Copper biding	XF9a_00870	uncharacterized protein, YfiH family	0.64463894	POLOX_XYLFA ame: Full=Polyphenol oxidase ame: Full=Multicopper oxidase XF_0940	260	5	2	F:copper ion binding; C:cytosol		IPR003730 (PFAM); IPR038371 (G3DSA:3.60.140.GENE3D); IPR003730 (TIGRFAM); PTHR30616:SF2 (PANTHER); IPR003730 (PANTHER); IPR011324 (SUPERFAMILY)

			XF9a_00872	Uncharacterized protein conserved in bacteria	0.914102568	Y1259_ARATH ame: Full=DCC family chloroplastic Flags: Precursor	150	2	1	C:plastoglobule		IPR007263 (PFAM); PTHR33639:SF2 (PANTHER); PTHR33639 (PANTHER)
			XF9a_00125	Predicted acyltransferases	0.641265353	---NA---	380					IPR002656 (PFAM); PTHR23028:SF53 (PANTHER); PTHR23028 (PANTHER)
			XF9a_01725	Uncharacterized conserved protein	-0.621647161	Y473_VIBC3 ame: Full=UPF0312 VC0395_0473 VC395_A0785 Flags: Precursor	237	5	4	P:biological_process; C:periplasmic space; C:cellular_component; F:molecular_function		IPR007372 (SMART); IPR007372 (PFAM); IPR036761 (G3DSA:2.40.128.GENE3D); PTHR34406 (PANTHER); IPR036761 (SUPERFAMILY)
			XF9a_01038	TIGR02099 family protein	0.486464537	YHDP_ECOLIRecName: Full=Uncharacterized protein YhdP; Flags: Precursor	1291	1				IPR032712 (PFAM); IPR011836 (TIGRFAM); IPR025263 (PFAM); IPR011836 (PANTHER)
			XF9a_01165	hypothetical protein	-0.451038693	---NA---	293					SSF82171 (SUPERFAMILY)
			XF9a_01240	hypothetical protein	0.760319939	---NA---	159					no IPS match
			XF9a_01241	Uncharacterized protein required for cytochrome oxidase assembly	0.653412489	---NA---	387					IPR003780 (PFAM); PTHR35457 (PANTHER)
			XF9a_01460	hypothetical protein	-0.997445841	---NA---	82					no IPS match
			XF9a_01903	hypothetical protein	0.642951999	---NA---	182					no IPS match
			XF9a_01997	hypothetical protein	0.662464265	---NA---	80					no IPS match
			XF9a_01998	hypothetical protein	0.833060274	---NA---	83					no IPS match
			XF9a_02098	hypothetical protein	0.579292064	---NA---	149					no IPS match
			XF9a_02150	hypothetical protein	0.743111509	---NA---	61					no IPS match
Total: 69			XF9a_00562	hypothetical protein	0.708776861	---NA---	96					no IPS match

Table S5. Differentially expressed genes in the phytopathogen *Mm* only and co-culture.

Class	Genic Category	SeqName	Ncbi Locus tag	Gene product name	Description Blast2go	log2FoldChange	Length	#Hits	e-Value	sim mean	#GO	GO Names list		Enzyme Codes list	InterPro IDs
Macromolecule metabolism	DNA metabolism	DNA replication	MMSR116_RS14125	IS1182 family transposase	RFCS_PICTO ame: Full=Replication factor C small subunit Short=RFC small subunit ame: Full=Clamp loader small subunit	1.952894924	478	1	8.65E-3	56%	6	F:nucleotide binding; F:DNA binding; F:ATP binding; F:DNA clamp loader activity; P:DNA replication; C:DNA replication factor C complex			no IPS match
	DNA metabolism	DNA replication	MMSR116_RS04575	DNA methylase family protein	MTB1_BRUME ame: Full=Modification methylase Short= ame: Full=Adenine-specific methyltransferase ame:	-1.460170741	444	5	9.78E-16	49%	10	F:nucleic acid binding; F:DNA binding; P:DNA methylation; F:N-methyltransferase activity; P:DNA replication; F:methyltransferase activity; F:transferase activity; P:DNA			IPR002187 (PRINTS); IPR002187 (SMART);

					Full=DNA methyltransferase ame: Full= ortholog							methylation on adenine; F:site-specific DNA-methyltransferase (adenine-specific) activity; P:methylation			IPR015867 (G3DSA:3.30.70. GENE3D); IPR002187 (PIRSF); IPR002187 (PFAM); PTHR30115:SF17 (PANTHER); IPR002187 (PANTHER); IPR002187 (PROSITE_PROFILES); IPR011322 (SUPERFAMILY)
	DNA metabolism	DNA replication	MMSR116_RS06775	ribonucleoside-diphosphate reductase, adenosylcobalamin-dependent	NRDJ_BRADU ame: Full=Vitamin B12-dependent ribonucleotide reductase ame: Full=Ribonucleoside-diphosphate reductase	-0.93963467	1243	5	0.00E+00	72.8%	9	F:nucleotide binding; F:ribonucleoside-diphosphate reductase activity, thioredoxin disulfide as acceptor; F:cobalamin binding; P:DNA replication; P:deoxyribonucleotide biosynthetic process; P:DNA biosynthetic process; P:oxidation-reduction process; C:ribonucleoside-diphosphate reductase complex; F:cobalt ion binding			IPR003846 (PFAM); PTHR32057 (PANTHER); IPR003846 (HAMAP)
	DNA metabolism	DNA replication	MMSR116_RS05125	DUF4112 domain-containing protein	DPB2_KLULA ame: Full=DNA polymerase epsilon subunit B ame: Full=DNA polymerase II subunit 2	1.573558555	167	1	9.46E-2	48%	15	F:nucleotidyltransferase activity; P:DNA replication; P:DNA-dependent DNA replication; F:double-stranded DNA binding; P:DNA-dependent DNA replication maintenance of fidelity; P:mitotic DNA replication initiation; P:error-prone translesion synthesis; C:epsilon DNA polymerase complex; F:DNA binding; C:nucleus; F:DNA-directed DNA polymerase activity; C:nuclear chromatin; C:cytoplasm; C:cytosol; F:transferase activity			IPR010707 (PIRSF); G3DSA:3.10.540.10 (GENE3D); IPR023361 (G3DSA:2.30.270.GENE3D); IPR010707 (PFAM)
	DNA metabolism	DNA replication	MMSR116_RS14985	nucleoid-associated protein, YbaB/EbfC family	Y275_METSB ame: Full=Nucleoid-associated Msil_0275	1.159032008	107	5	1.27E-32	77%	3	F:DNA binding; C:bacterial nucleoid; C:cytoplasm			IPR027275 (PFAM); G3DSA:2.30.30.240 (GENE3D); PTHR36505 (PANTHER); IPR011033 (SUPERFAMILY)
	DNA metabolism	nucleic acid metabolism	MMSR116_RS06070	HD domain-containing protein	SPOT_HAEIN ame: Full=Guanosine-3 ,5 -bis(diphosphate) 3 -pyrophosphohydrolase ame: Full=Penta-phosphate guanosine-3 -pyrophosphohydrolase Short=(ppGpp)ase	1.453764245	164	5	5.57E-4	44.4%	16	P:guanosine tetraphosphate biosynthetic process; P:metabolic process; P:response to starvation; F:guanosine-3',5'-bis(diphosphate) 3'-diphosphatase activity; C:plasma membrane; F:GTP diphosphokinase activity; F:nucleotide binding; F:GTP binding; F:ATP binding; C:cell wall; P:guanosine tetraphosphate metabolic process; P:phosphorylation; F:amino acid binding; F:transferase activity; F:kinase activity; F:hydrolase activity			IPR018648 (PFAM)
	DNA metabolism	DNA repair	MMSR116_RS22200	DNA repair protein MmcB-related protein	---NA---	1.144702594	170							EC:4.2.1.1	IPR001765 (SMART); IPR036874 (G3DSA:3.40.1050.GENE3D); IPR001765 (PFAM); PTHR11002 (PANTHER); PTHR11002:SF13 (PANTHER); IPR036874 (SUPERFAMILY)
	DNA metabolism	Plasmid partition protein A	MMSR116_RS02080	ParA family protein	VIRC1_AGRRH ame: Full= virC1	-1.609143843	215	5	2.22E-6	47.6%	13	F:ATPase activity; P:nucleic acid phosphodiester bond hydrolysis; F:nucleotide binding; F:DNA binding; F:ATP binding; F:protein binding; P:anion transmembrane transport; P:response to arsenic-containing substance; F:endonuclease activity; P:cation transmembrane transport; P:detoxification of arsenic-containing substance; F:hydrolase activity; F:ATPase-coupled arsenite transmembrane transporter activity			IPR008807 (PFAM); G3DSA:1.10.10.1550 (GENE3D)
	RNA metabolism	ribossomal silencing factors	MMSR116_RS22505	Uma2 family endonuclease	Y925_SYNY3RecName: Full=Uncharacterized protein sll0925	1.31493907	187	3	6.95E-4	51.67%					IPR031811 (PFAM)
	RNA metabolism		MMSR116_RS30410	ribonuclease P protein component	RNPA_RUEPO ame: Full=Ribonuclease P component Short=RNase P Short=R ame: Full= C5	-1.324393197	204	4	1.55E-4	61%	11	P:RNA phosphodiester bond hydrolysis, endonucleolytic; P:nucleic acid phosphodiester bond hydrolysis; F:ribonuclease P activity; P:RNA phosphodiester bond hydrolysis; F:RNA binding; P:tRNA processing;			IPR000572 (PFAM); IPR036374 (G3DSA:3.90.42

												F:endonuclease activity; F:nuclease activity; F:tRNA binding; P:tRNA 5'-leader removal; F:hydrolase activity		0.GENE3D); PTHR43032:SF3 (PANTHER); PTHR43032 (PANTHER); IPR022867 (HAMAP); IPR006311 (PROSITE_PROFILES); IPR036374 (SUPERFAMILY)
	RNA metabolism	Regulation of gene expression (stress)	MMSR116_RS00525	ECF subfamily RNA polymerase sigma-24 factor	SIGK_MYCSS ame: Full=RNA polymerase sigma factor ame: Full=Sigma-K factor	-1.454061703	185	5	4.17E-29	54.6%	5	P:regulation of gene expression; F:binding; P:regulation of primary metabolic process; P:regulation of cellular metabolic process; C:cell periphery		PR00173 (PRINTS); IPR001991 (PFAM); IPR036458 (G3DSA:1.10.3860.GENE3D); PTHR42865:SF1 (PANTHER); PTHR42865 (PANTHER); IPR023954 (HAMAP); IPR036458 (SUPERFAMILY)
	RNA metabolism		MMSR116_RS05315	transcription termination factor Rho	RHO_RHOS4 ame: Full=Transcription termination factor Rho ame: Full=ATP-dependent helicase Rho	-1.37334379	428	5	0.00E+00	87%	6	F:ATP binding; F:RNA binding; F:RNA-dependent ATPase activity; P:regulation of transcription, DNA-templated; F:helicase activity; P:DNA-templated transcription, termination		IPR011129 (SMART); IPR003593 (SMART); IPR011112 (SMART); G3DSA:3.40.50.300 (GENE3D); IPR011112 (PFAM); IPR004665 (TIGRFAM); IPR000194 (PFAM); IPR011113 (PFAM); G3DSA:1.10.720.10 (GENE3D); G3DSA:2.40.50.140 (GENE3D); PTHR15184 (PANTHER); IPR004665 (PTHR15184:PANTHER); IPR004665 (HAMAP); IPR027417 (SUPERFAMILY); IPR036269 (SUPERFAMILY); IPR012340 (SUPERFAMILY)
	RNA metabolism	Transcription	MMSR116_RS11170	RNA polymerase sigma factor RpoH	RPOH_RHIRD ame: Full=RNA polymerase sigma factor ame: Full=RNA polymerase sigma-32 factor	1.44455512	297	5	6.21E-152	74.8%	9	P:transcription initiation from bacterial-type RNA polymerase promoter; F:DNA binding; F:sigma factor activity; F:transcription factor activity, sequence-specific DNA binding; F:zinc ion binding; P:response to heat; C:cytoplasm; P:regulation of transcription, DNA-templated; P:sporulation resulting in formation of a cellular spore		IPR002433 (PRINTS); IPR000183 (PRINTS); IPR022643 (PFAM); IPR029066 (G3DSA:3.20.20.GENE3D); IPR009006 (G3DSA:2.40.37.GENE3D); IPR022644 (PFAM); PTHR11482 (PANTHER); PTHR11482:SF6

															(PANTHER); IPR029066 (SUPERFAMILY); IPR009006 (SUPERFAMILY)
	RNA metabolism	Transcription	MMSR116_RS04830	DNA-binding response regulator	ACTR_SINMW ame: Full=Acid tolerance regulatory	2.094536867	199	5	5.61E-84	82.4%	6	P:transcription, DNA-templated; P:phosphorelay signal transduction system; P:regulation of transcription, DNA-templated; F:sequence-specific DNA binding; C:intracellular; F:phosphorelay response regulator activity			no IPS match
	RNA metabolism	transcriptional regulator	MMSR116_RS05115	MucR family transcriptional regulator	MUCR_RHIME ame: Full=Transcriptional regulatory	1.216525504	180	5	3.24E-33	65.6%	1	F:binding			G3DSA:3.40.1740.10 (GENE3D); IPR003774 (PFAM); IPR003774 (PANTHER); IPR003774 (HAMAP); SSF143456 (SUPERFAMILY)
	RNA metabolism	transcriptional regulator	MMSR116_RS26055	MucR family transcriptional regulator	MUCR_RHIME ame: Full=Transcriptional regulatory	1.191641256	153	5	4.79E-38	64.2%	1	F:binding		EC:3.4.18	IPR038063 (G3DSA:2.40.440.GENE3D); IPR005490 (PFAM); PTHR30582:SF24 (PANTHER); PTHR30582 (PANTHER); PS51257 (PROSITE_PROFILES); IPR038063 (SUPERFAMILY)
	RNA metabolism	transcriptional regulator	MMSR116_RS18630	MucR family transcriptional regulator	MUCR_RHIME ame: Full=Transcriptional regulatory	1.267343676	153	5	6.5E-30	64.4%	1	F:binding			IPR001789 (SMART); IPR001789 (PFAM); IPR002197 (PFAM); G3DSA:1.10.10.60 (GENE3D); G3DSA:3.40.50.2300 (GENE3D); PTHR32071:SF51 (PANTHER); PTHR32071 (PANTHER); IPR001789 (PROSITE_PROFILES); IPR011006 (SUPERFAMILY)
	RNA metabolism	transcriptional regulator	MMSR116_RS07440	MucR family transcriptional regulator	ROS_RHIRD ame: Full=Transcriptional regulatory ros	1.99224614	164	5	9.62E-27	64%	1	F:binding		EC:1.1.99.14	IPR004113 (PFAM); IPR016171 (G3DSA:1.10.45.GENE3D); G3DSA:3.30.465.40 (GENE3D); G3DSA:3.30.70.2740 (GENE3D); IPR006094 (PFAM); PTHR42934:SF1 (PANTHER); PTHR42934 (PANTHER); IPR016166 (PROSITE_PROFILES); IPR036318 (SUPERFAMILY); IPR016164 (SUPERFAMILY)
	RNA metabolism	transcriptional regulator - metal sensing regulator	MMSR116_RS14485	MerR family DNA-binding transcriptional regulator	ZNTR_ECO57 ame: Full=HTH-type transcriptional regulator ame: Full=Zn(II)-responsive regulator of zntA	1.183609797	140	5	1.45E-24	58.2%	3	F:transcription factor activity, sequence-specific DNA binding; P:positive regulation of transcription, DNA-templated; F:binding			IPR021273 (PFAM)

	RNA metabolism	transcriptional regulator - stress response	MMSR116_RS00375	Crp/Fnr family transcriptional regulator	FNRL_RHOS4 ame: Full=Transcriptional activator	1.364127111	246	5	1.96E-13	44.8%	6	F:DNA binding; F:transcription factor activity, sequence-specific DNA binding; P:transcription, DNA-templated; P:regulation of transcription, DNA-templated; P:nitrogen fixation; C:intracellular		EC:1.8.4.11	IPR036509 (G3DSA:3.30.1060.GENE3D); IPR002569 (PFAM); IPR002569 (TIGRFAM); PTHR42799 (PANTHER); PTHR42799:SF6 (PANTHER); IPR002569 (HAMAP); IPR036509 (SUPERFAMILY)
	RNA metabolism	transcriptional regulator - stress response	MMSR116_RS30165	bifunctional DNA-binding transcriptional regulator/O6-methylguanine-DNA methyltransferase Ada	ADA_ECOLI ame: Full=Bifunctional transcriptional activator DNA repair enzyme Ada ame: Full=Regulatory of adaptive response Includes: ame: Full=Methylphosphotriester-DNA-- -cysteine S-methyltransferase ame: Full=Methylphosphotriester-DNA methyltransferase Includes: ame: Full=Methylated-DNA-- -cysteine methyltransferase ame: Full=O6-methylguanine-DNA alkyltransferase	1.631496098	369	5	7.49E-115	64.8%	8	P:DNA dealkylation involved in DNA repair; F:zinc ion binding; F:protein binding; P:DNA demethylation; P:response to nitrosative stress; F:methylated-DNA-[protein]-cysteine S-methyltransferase activity; F:damaged DNA binding; C:plasma membrane		EC:3.6.1.3	IPR002586 (PFAM); IPR010223 (TIGRFAM); G3DSA:3.40.50.300 (GENE3D); IPR025501 (PIRSF); PTHR43384 (PANTHER); PTHR43384:SF1 (PANTHER); IPR027417 (SUPERFAMILY)
	RNA metabolism	transcription	MMSR116_RS23260	methyltransferase domain-containing protein	ARSR_ECOLI ame: Full=Arsenical resistance operon repressor	1.569178409	336	5	2.47E-12	62.2%	4	F:DNA binding; P:transcription, DNA-templated; P:response to arsenic-containing substance; P:regulation of transcription, DNA-templated			no IPS match
	RNA metabolism	transcriptional regulator	MMSR116_RS08620	transcriptional regulator	YDEP_BACSU ame: Full=Uncharacterized HTH-type transcriptional regulator	1.71309569	121	5	3.58E-22	61%	3	F:DNA binding; P:transcription, DNA-templated; P:regulation of transcription, DNA-templated			no IPS match
	RNA metabolism	transcriptional regulator	MMSR116_RS06015	DNA-binding response regulator	OMPR_ECOLI ame: Full=Transcriptional regulatory	1.417934447	239	5	3.89E-55	59.8%	7	F:transcription factor activity, sequence-specific DNA binding; F:identical protein binding; P:negative regulation of transcription, DNA-templated; P:positive regulation of transcription, DNA-templated; P:phosphorelay signal transduction system; C:cytosol; F:phosphorelay response regulator activity			IPR008258 (PFAM); G3DSA:1.10.530.10 (GENE3D); IPR023346 (SUPERFAMILY)
	RNA metabolism	Transcription	MMSR116_RS28615	nitrogen regulatory protein P-II	GLNB_RHILO ame: Full=Nitrogen regulatory P-II	1.048806401	112	5	9.09E-66	91.8%	7	F:nucleotide binding; P:transcription, DNA-templated; P:regulation of catalytic activity; P:regulation of transcription, DNA-templated; P:regulation of nitrogen utilization; P:nitrogen fixation; F:enzyme regulator activity			IPR002563 (PFAM); IPR012349 (G3DSA:2.30.110.GENE3D); PTHR43567:SF1 (PANTHER); PTHR43567 (PANTHER); SSF50475 (SUPERFAMILY)
	Protein metabolism	Translation	MMSR116_03410	peptide chain release factor 2	RF2_MAGSA ame: Full=Peptide chain release factor 2 Short=RF-2	-1.263522363	375	5	4.33E-176	76%	3	P:translational termination; C:cytoplasm; F:translation release factor activity, codon specific			no IPS match
	Protein metabolism	Translation	MMSR116_RS20855	50S ribosomal protein L28	RL28_METRJ ame: Full=50S ribosomal L28	-1.750003006	100	5	8.81E-66	92.6%	3	F:structural constituent of ribosome; C:ribosome; P:translation			IPR003684 (PFAM)
	Protein metabolism	Translation	MMSR116_RS10555	30S ribosomal protein S20	RS20_METRJ ame: Full=30S ribosomal S20	-1.264945184	88	5	2.18E-48	94.2%	4	F:structural constituent of ribosome; C:ribosome; F:rRNA binding; P:translation		EC:1.3.99.7	G3DSA:2.40.110.10 (GENE3D); IPR009075 (PFAM); IPR013786 (PFAM); IPR006091 (PFAM); G3DSA:1.20.140.10 (GENE3D); IPR037069 (G3DSA:1.10.54

															0.GENE3D); PTHR42807 (PANTHER); IPR009100 (SUPERFAMILY); IPR036250 (SUPERFAMILY)
	Protein metabolism	translation	MMSR116_RS03090	30S ribosomal protein S21	RS21_METNO ame: Full=30S ribosomal S21	-1.164760906	111	5	2.07E-28	90.8%	3	C:ribosomal subunit; F:structural constituent of ribosome; P:translation		EC:1.2.4.2	IPR005475 (SMART); IPR032106 (PFAM); IPR001017 (PFAM); G3DSA:3.40.50.11610 (GENE3D); IPR011603 (TIGRFAM); G3DSA:1.10.287.1150 (GENE3D); G3DSA:3.40.50.12470 (GENE3D); IPR031717 (PFAM); IPR005475 (PFAM); IPR011603 (PIRSF); G3DSA:3.40.50.970 (GENE3D); IPR011603 (PANTHER); IPR029061 (SUPERFAMILY); IPR029061 (SUPERFAMILY)
	Protein metabolism	translation	MMSR116_RS25435	RNA helicase	RHLE_ECOLI ame: Full=ATP-dependent RNA helicase	-1.643055132	511	5	1.61E-119	63.6%	9	F:nucleotide binding; P:response to heat; F:RNA strand-exchange activity; F:RNA strand annealing activity; F:ATP-dependent 5'-3' RNA helicase activity; P:RNA secondary structure unwinding; C:cytosol; P:ribosome assembly; F:ATP-dependent 3'-5' RNA helicase activity			IPR010105 (TIGRFAM); IPR036942 (G3DSA:2.40.170.GENE3D); IPR037066 (G3DSA:2.170.130.GENE3D); IPR000531 (PFAM); IPR012910 (PFAM); PTHR32552:SF65 (PANTHER); PTHR32552 (PANTHER); SSF56935 (SUPERFAMILY)
	Protein metabolism	translation	MMSR116_RS25130	quinoprotein dehydrogenase-associated SoxYZ-like	SYP_HALOH ame: Full=Proline--tRNA ligase ame: Full=Prolyl-tRNA synthetase Short=	1.795242569	279	1	7.26E-2	38%	11	F:nucleotide binding; P:tRNA aminoacylation for protein translation; F:ATP binding; F:aminoacyl-tRNA ligase activity; C:cytoplasm; P:regulation of translational fidelity; F:proline-tRNA ligase activity; F:aminoacyl-tRNA editing activity; P:prolyl-tRNA aminoacylation; F:ligase activity; P:translation			IPR029032 (G3DSA:1.20.1290.GENE3D); PTHR34846 (PANTHER); PTHR34846:SF2 (PANTHER); IPR029032 (SUPERFAMILY)
	Protein metabolism	nucleotide biding	MMSR116_RS12120	ATP-dependent helicase	DEAD_BUCBP ame: Full=ATP-dependent RNA helicase ame: Full=Cold-shock DEAD box A	-1.280011433	584	5	5.67E-69	48.2%	3	F:nucleic acid binding; F:helicase activity; P:cellular process			IPR030395 (PFAM); IPR017946 (G3DSA:3.20.20.GENE3D); PTHR43620:SF7 (PANTHER); PTHR43620 (PANTHER); IPR030395 (PROSITE_PROFI

															LES); IPR017946 (SUPERFAMILY)
	Protein metabolism	Protein folding	MMSR116_RS20870	molecular chaperone DnaJ	YCB2_PSEDE ame: Full=Uncharacterized kDa in cobS 5 region ame: Full=ORF2	1.080068559	206	5	3.42E-43	65.8%	1	F:ion binding			IPR000100 (PFAM); IPR000100 (TIGRFAM); IPR014721 (G3DSA:3.30.230.GENE3D); IPR000100 (PANTHER); IPR000100 (HAMAP); IPR020568 (SUPERFAMILY)
	Protein metabolism	Protein biding	MMSR116_RS22230	tetratricopeptide repeat-containing protein	Y940_METJA ame: Full=TPR repeat-containing MJ0940	-1.21586595	290	5	1.21E-17	47.6%	19	P:positive regulation of cellular protein metabolic process; F:UDP-glycosyltransferase activity; P:response to organic substance; P:carbohydrate derivative metabolic process; P:regulation of biosynthetic process; P:response to oxygen-containing compound; P:regulation of carbohydrate metabolic process; C:nucleus; F:protein binding; P:cellular glucose homeostasis; P:rhythmic process; C:cytoplasm; P:regulation of protein phosphorylation; P:histone modification; P:regulation of gene expression; P:cellular response to stimulus; F:N-acetyltransferase activity; P:negative regulation of protein modification process; P:peptidyl-lysine modification			IPR032807 (PFAM); PTHR32309 (PANTHER); PTHR32309:SF13 (PANTHER)
	Protein metabolism	Post translation modification	MMSR116_RS02310	isoprenylcysteine carboxylmethyltransferase	NRM_XENLA ame: Full=Nurim ame: Full=Nuclear envelope membrane ame: Full=Nuclear rim	1.828852014	244	5	1.21E-10	48.6%	2	C:nucleus; C:membrane			IPR018661 (PFAM)
	Protein metabolism	novel chaperonins	MMSR116_RS13530	ABC-1 domain protein	COQ8_YEAST ame: Full=Atypical kinase mitochondrial ame: Full=Activity of bc1 complex 1 ame: Full=Coenzyme Q 8 ame: Full=Ubiquinone biosynthesis COQ8 Flags: Precursor	1.153805314	453	5	1.13E-31	47.8%	6	C:mitochondrial part; C:membrane; P:phosphorylation; F:binding; F:kinase activity; P:organic substance metabolic process			IPR027405 (G3DSA:1.10.10.GENE3D); IPR009282 (PFAM); IPR027405 (SUPERFAMILY)
	Protein metabolism	chaperone	MMSR116_RS29890	cytochrome c maturation protein CcmE	CCME_METRJ ame: Full=Cytochrome c-type biogenesis ame: Full=Cytochrome c maturation E ame: Full=Heme chaperone	1.120750101	170	5	1.78E-102	90.4%	5	F:metal ion binding; C:integral component of membrane; P:protein-heme linkage; P:cytochrome complex assembly; C:plasma membrane			IPR013785 (G3DSA:3.20.20.GENE3D); IPR008567 (PFAM); IPR008567 (PANTHER); PTHR37418:SF2 (PANTHER)
Intermediary metabolism	Energy Production	electron transfer activity	MMSR116_RS19245	ATP synthase F0 subunit C	ATPL_JANSC ame: Full=ATP synthase subunit c ame: Full=ATP synthase F(0) sector subunit c ame: Full=F-type ATPase subunit c Short=F-ATPase subunit c ame: Full=Lipid-binding	-1.808372095	75	5	3.18E-6	73.4%	13	P:proton transport; P:ATP hydrolysis coupled proton transport; C:proton-transporting two-sector ATPase complex, proton-transporting domain; F:hydrogen ion transmembrane transporter activity; F:lipid binding; P:ATP biosynthetic process; P:transport; C:plasma membrane; P:ion transport; C:proton-transporting ATP synthase complex, coupling factor F(o); C:membrane; C:integral component of membrane; P:ATP synthesis coupled proton transport			IPR009531 (PFAM)
	Energy Production	electron transfer activity	MMSR116_RS22760	(2Fe-2S)-binding protein	IORA_BREDI ame: Full=Isoquinoline 1-oxidoreductase subunit alpha	1.677697423	151	5	3.00E-51	57.8%	6	P:aromatic compound catabolic process; F:oxidoreductase activity, acting on CH or CH2 groups; C:cytoplasm; F:oxidoreductase activity, acting on the aldehyde or oxo group of donors; P:oxidation-reduction process; F:2 iron, 2 sulfur cluster binding			IPR009593 (PIRSF); IPR009593 (PFAM)
	Energy Production	unknown fuction iron sulfer cluster	MMSR116_RS28290	YdiU family protein	Y5402_METPB ame: Full=UPF0061 Mpop_5402	1.178340739	489	5	0.00E+00	80%					IPR012910 (PFAM); IPR010105 (TIGRFAM); IPR037066 (G3DSA:2.170.130.GENE3D); IPR000531 (PFAM); IPR036942 (G3DSA:2.40.170.GENE3D); PTHR32552

															(PANTHER); PTHR32552:SF71 (PANTHER); SSF56935 (SUPERFAMILY)
	Energy Production		MMSR116_RS25545	iron-containing alcohol dehydrogenase	ADH2_ECOLI ame: Full=Probable alcohol dehydrogenase	1.211173299	387	5	1.36E-62	54%	10	F:magnesium ion binding; F:zinc ion binding; P:small molecule catabolic process; F:methanol dehydrogenase activity; P:organic substance catabolic process; P:alcohol metabolic process; P:oxidation-reduction process; C:cytoplasmic part; P:single-organism cellular process; P:cellular metabolic process		EC:2.1.1.63	IPR018060 (SMART); IPR036388 (G3DSA:1.10.10.GENE3D); G3DSA:3.30.160.70 (GENE3D); IPR014048 (PFAM); G3DSA:1.10.10.60 (GENE3D); IPR016221 (PIRSF); IPR018060 (PFAM); IPR004026 (PFAM); IPR014048 (TIGRFAM); IPR035451 (G3DSA:3.40.10.GENE3D); PTHR10815:SF9 (PANTHER); PTHR10815 (PANTHER); IPR018060 (PROSITE_PROFILES); IPR036217 (SUPERFAMILY); IPR009057 (SUPERFAMILY); IPR035451 (SUPERFAMILY); IPR036631 (SUPERFAMILY)
	Energy Production		MMSR116_RS26665	PQQ-dependent dehydrogenase, methanol/ethanol family	EXAA_PSEAE ame: Full=Quino alcohol dehydrogenase (cytochrome c) ame: Full=Quino alcohol dehydrogenase (cytochrome c550) ame: Full=Quino ethanol dehydrogenase Short=QEDH Flags: Precursor	-1.803819973	589	5	0.00E+00	63.4%	7	F:organic cyclic compound binding; F:heterocyclic compound binding; F:metal ion binding; P:ethanol oxidation; C:periplasmic space; F:alcohol dehydrogenase (acceptor) activity; F:alcohol dehydrogenase (cytochrome c) activity			IPR000639 (PRINTS); IPR000073 (PRINTS); IPR029058 (G3DSA:3.40.50.GENE3D); IPR000073 (PFAM); PTHR43329 (PANTHER); PTHR43329:SF17 (PANTHER); IPR029058 (SUPERFAMILY)
	Energy Production	electron-transfer proteins	MMSR116_RS26675	cytochrome c-550 PedF	QHED_PSEPU ame: Full=Quinohemo alcohol dehydrogenase ADH IIB Short=ADH IIB ame: Full=Alcohol dehydrogenase (azurin) Flags: Precursor	-2.000650625	139	5	1.05E-5	52%	20	F:pyrroloquinoline quinone binding; F:oxidoreductase activity, acting on CH-OH group of donors; F:electron carrier activity; C:periplasmic space; C:plasma membrane; F:heme binding; C:thylakoid; C:thylakoid lumen; F:iron ion binding; F:metal ion binding; F:oxidoreductase activity; F:calcium ion binding; C:membrane; C:integral component of membrane; P:oxidation-reduction process; P:photosynthesis; C:outer membrane-bounded periplasmic space; F:ethanol:cytochrome c oxidoreductase activity; F:2-chloroethanol:cytochrome c oxidoreductase activity; F:alcohol dehydrogenase (cytochrome c) activity			IPR038380 (G3DSA:1.20.5.GENE3D); IPR001911 (TIGRFAM); IPR001911 (PFAM); PTHR21109:SF0 (PANTHER); IPR001911 (PANTHER); IPR001911 (PRODOM); IPR001911 (HAMAP)
	Energy Production	electron-transfer proteins	MMSR116_RS23965	cytochrome c family protein	CYC2_BLAVI ame: Full=Cytochrome c2 Flags: Precursor	-1.062926614	123	5	4.58E-27	67.2%	6	F:metal ion binding; F:electron carrier activity; P:oxidation-reduction process; P:photosynthesis; C:periplasmic space; F:heme binding			no IPS match

	Energy Production	electron-transfer proteins	MMSR116_RS23965	cytochrome c family protein	CYC2_BLAVI ame: Full=Cytochrome c2 Flags: Precursor	-1.062926614	123	5	4.58E-27	67.2%	6	F:metal ion binding; F:electron carrier activity; P:oxidation-reduction process; P:photosynthesis; C:periplasmic space; F:heme binding		no IPS match
	Energy Production	NAD(+) biosynthesis	MMSR116_RS22325	alkene reductase	NEMA_ECOLI ame: Full=N-ethylmaleimide reductase ame: Full=N-ethylmaleimide reducing enzyme	1.10349884	364	5	3.63E-90	57.6%	9	P:monocarboxylic acid biosynthetic process; F:N-ethylmaleimide reductase activity; F:FMN binding; P:nitroglycerin metabolic process; P:2,4,6-trinitrotoluene catabolic process; F:chromate reductase activity; C:cytosol; F:oxidoreductase activity, acting on other nitrogenous compounds as donors, with NAD or NADP as acceptor; P:fatty acid metabolic process		IPR004147 (PFAM); PTHR43851:SF3 (PANTHER); PR034646 (PANTHER); IPR011009 (SUPERFAMILY)
	Energy Production	NAD(+) biosynthesis	MMSR116_RS13000	aldo/keto reductase	Y2408_MYCS2 ame: Full=Uncharacterized oxidoreductase MSMEG_2408 MSMEI_2347	1.256531541	276	5	9.93E-99	67.6%	2	F:oxidoreductase activity; P:oxidation-reduction process		IPR036909 (G3DSA:1.10.760.GENE3D); IPR009056 (PFAM); IPR030991 (TIGRFAM); PTHR37823 (PANTHER); PTHR37823:SF1 (PANTHER); IPR009056 (PROSITE_PROFILES); IPR036909 (SUPERFAMILY)
	Energy Production	NAD(+) biosynthesis	MMSR116_RS20305	KR domain-containing protein	YXNA_BACSU ame: Full=Uncharacterized oxidoreductase	1.241503923	337	5	5.69E-26	45.4%	7	P:cellular ketone metabolic process; F:oxidoreductase activity, acting on the CH-OH group of donors, NAD or NADP as acceptor; C:intracellular membrane-bounded organelle; C:membrane; C:cytoplasmic part; P:ammonium ion metabolic process; P:sphingolipid metabolic process		no IPS match
	Energy Production	NAD(+) biosynthesis	MMSR116_RS30315	KR domain-containing protein	YVAG_BACSU ame: Full=Uncharacterized oxidoreductase	1.015049458	265	5	7.04E-81	54.6%	2	F:oxidoreductase activity, acting on the CH-OH group of donors, NAD or NADP as acceptor; P:single-organism metabolic process		G3DSA:3.40.630.10 (GENE3D); IPR017439 (TIGRFAM); IPR011650 (PFAM); IPR017439 (PIRSF); G3DSA:3.30.70.360 (GENE3D); IPR002933 (PFAM); PTHR11014:SF141 (PANTHER); PTHR11014 (PANTHER); IPR036264 (SUPERFAMILY); SSF53187 (SUPERFAMILY)
	Energy Production	NAD(+) biosynthesis	MMSR116_RS27740	flavin reductase family protein	YDFE_BACSURecName: Full=Uncharacterized protein YdfE	1.58749893	249	2	4.76E-45	53.5%	4	F:oxidoreductase activity, acting on the CH-NH group of donors, NAD or NADP as acceptor; F:FMN binding; F:oxidoreductase activity; P:oxidation-reduction process		PR01036 (PRINTS); IPR011701 (PFAM); G3DSA:1.20.1720.10 (GENE3D); G3DSA:1.20.1250.20 (GENE3D); PTHR23501 (PANTHER); PTHR23501:SF106 (PANTHER); IPR020846 (PROSITE_PROFILES); IPR036259 (SUPERFAMILY)
	Energy Production	NAD(+) biosynthesis	MMSR116_RS08300	flavin reductase family protein	P152_METTH ame: Full= MTH_152	1.119533127	230	5	1.26E-46	46.6%	4	F:oxidoreductase activity, acting on the CH-NH group of donors, NAD or NADP as acceptor; F:FMN binding; F:oxidoreductase activity; P:oxidation-reduction process	EC:3.3.2.10	IPR000639 (PRINTS); IPR000073 (PRINTS); IPR000073 (PFAM); IPR029058 (G3DSA:3.40.50.GENE3D);

													PTHR42977 (PANTHER); IPR029058 (SUPERFAMILY)
	Energy Production	FAD / lipid	MMSR116_RS29190	hypothetical protein	ACDB_BACSU ame: Full=Acyl-dehydrogenase	0.991932845	386	5	1.95E-8	41.6%	21	F:oxidoreductase activity, acting on the CH-CH group of donors; P:lipid homeostasis; F:electron carrier activity; F:butyryl-CoA dehydrogenase activity; P:metabolic process; P:butyrate catabolic process; P:fatty acid metabolic process; C:mitochondrial inner membrane; F:acyl-CoA dehydrogenase activity; P:fatty acid beta-oxidation using acyl-CoA dehydrogenase; C:mitochondrial matrix; F:flavin adenine dinucleotide binding; F:oxidoreductase activity; F:fatty-acyl-CoA binding; F:oxidoreductase activity, acting on the CH-CH group of donors, with a flavin as acceptor; C:mitochondrion; P:lipid metabolic process; C:membrane; P:oxidation-reduction process; P:dibenzothiophene catabolic process; P:sporulation resulting in formation of a cellular spore	IPR021327 (PFAM)
	Energy Production	NAD(+) biosynthesis	MMSR116_RS22275	NADH:flavin oxidoreductase/NADH oxidase	AFVA_ASPFN ame: Full=NADPH dehydrogenase afvA ame: Full=Aflavarin synthesis A	1.010081861	376	5	5.07E-93	60%	1	F:oxidoreductase activity	IPR014824 (SMART); IPR035433 (PIRSF); IPR036498 (G3DSA:3.30.1370.GENE3D); IPR034904 (G3DSA:3.30.300.GENE3D); IPR014824 (PFAM); IPR001075 (PFAM); PTHR11178:SF1 (PANTHER); PTHR11178 (PANTHER); IPR001075 (PRODOM); IPR036498 (SUPERFAMILY); IPR034904 (SUPERFAMILY)
	Energy Production	NAD(+) biosynthesis	MMSR116_RS05670	NAD(P)H-dependent oxidoreductase	CHRR_PSEPK ame: Full=Quinone reductase ame: Full=Chromate reductase Short=CHRR ame: Full=NAD(P)H dehydrogenase (quinone)	1.676857731	192	5	2.77E-8	45.4%	14	F:identical protein binding; F:FMN binding; F:transcription factor binding; P:cytoplasmic sequestering of transcription factor; F:FMN reductase (NADH) activity; C:nucleus; F:FMN reductase (NADPH) activity; F:NAD(P)H dehydrogenase (quinone) activity; C:cytoplasm; F:oxidoreductase activity; P:apoptotic process; C:cytosol; P:cellular response to oxidative stress; P:oxidation-reduction process	IPR032789 (PFAM)
	Energy Production	NADP/FAD	MMSR116_RS23265	methylenetetrahydrofolate reductase [NAD(P)H]	METF_SALTY ame: Full=5,10-methylenetetrahydrofolate reductase	1.544864623	305	5	2.59E-101	70.6%	8	F:FAD binding; P:protein homotetramerization; C:cytosol; P:cellular amino acid biosynthetic process; P:tetrahydrofolate biosynthetic process; F:methylenetetrahydrofolate reductase (NAD(P)H) activity; P:one-carbon metabolic process; P:methionine metabolic process	IPR025833 (PFAM)
	Energy Production	NAD(+) biosynthesis	MMSR116_RS09625	hypothetical protein	NADHK_ARATH ame: Full=NADH kinase Short= -3	1.702770295	297	5	1.86E-4	45.4%	14	F:NADH kinase activity; P:metabolic process; P:NADP biosynthetic process; F:nucleotide binding; F:NAD+ kinase activity; F:ATP binding; C:cytoplasm; F:metal ion binding; P:pyridine nucleotide biosynthetic process; P:NAD metabolic process; C:cytosol; P:phosphorylation; F:transferase activity; F:kinase activity	G3DSA:2.40.110.10 (GENE3D); IPR037069 (G3DSA:1.10.540.GENE3D); PIRSF016578 (PIRSF); IPR013786 (PFAM); G3DSA:1.20.140.10 (GENE3D); PTHR43835 (PANTHER); PTHR43835:SF9 (PANTHER); IPR036250 (SUPERFAMILY); IPR009100 (SUPERFAMILY)

	Energy Production	FAD	MMSR116_RS05110	FAD-binding oxidoreductase	GLCD_ECOL6 ame: Full=Glycolate oxidase subunit	1.399373638	477	5	1.24E-124	53.6%	5	P:glycolate catabolic process; C:membrane; F:glycolate dehydrogenase activity; C:cytoplasmic part; P:cellular response to DNA damage stimulus			no IPS match
	Energy Production	NADP	MMSR116_RS08270	aldo/keto reductase	YRPG_BACSU ame: Full=Uncharacterized oxidoreductase	-2.345369915	329	5	1.12E-128	54.4%	4	C:cytoplasm; C:membrane; P:single-organism metabolic process; P:organic substance metabolic process			IPR003593 (SMART); G3DSA:3.40.50.300 (GENE3D); IPR003439 (PFAM); PTHR42794 (PANTHER); IPR003439 (PROSITE_PROFILES); IPR027417 (SUPERFAMILY)
	Energy Production	NADP/FAD	MMSR116_RS02450	dehydrogenase	FDHA_DESGI ame: Full=Formate dehydrogenase subunit alpha Short=FDH subunit alpha ame: Full=Formate dehydrogenase large subunit Flags: Precursor	1.302504318	197	5	2.99E-33	57.8%	15	F:formate dehydrogenase (quinone) activity; P:anaerobic respiration; F:selenium binding; F:electron carrier activity; F:molybdenum ion binding; P:cellular response to DNA damage stimulus; C:formate dehydrogenase complex; P:molybdenum incorporation into iron-sulfur cluster; F:protein binding; C:membrane; F:formate dehydrogenase (cytochrome-c-553) activity; C:cytosol; C:outer membrane-bounded periplasmic space; F:4 iron, 4 sulfur cluster binding; P:formate oxidation			IPR018677 (PFAM)
	Energy Production	NAD(+) biosynthesis	MMSR116_RS12995	quinolinate synthase NadA	NADA2_RHILO ame: Full=Quinolinate synthase A 2	2.077021219	338	5	6.78E-172	84.6%	7	P:quinolinate biosynthetic process; C:cytoplasm; F:metal ion binding; P:NAD biosynthetic process; F:4 iron, 4 sulfur cluster binding; F:quinolinate synthetase A activity; F:transferase activity, transferring alkyl or aryl (other than methyl) groups			IPR001296 (PFAM); G3DSA:3.40.50.2000 (GENE3D); G3DSA:3.40.50.2000 (GENE3D); IPR028098 (PFAM); PTHR12526 (PANTHER); PTHR12526:SF554 (PANTHER); SSF53756 (SUPERFAMILY)
	Energy Production	NAD(+) biosynthesis	MMSR116_RS19090	NADH dehydrogenase	NUON_DESAP ame: Full=NADH-quinone oxidoreductase subunit N ame: Full=NADH dehydrogenase I subunit N ame: Full=NDH-1 subunit N	-1.187346084	584	5	1.35E-22	49.4%	1	P:transport			IPR036526 (G3DSA:3.60.110.GENE3D); IPR003010 (PFAM); PTHR23088 (PANTHER); PTHR23088:SF27 (PANTHER); IPR003010 (PROSITE_PROFILES); IPR036526 (SUPERFAMILY)
	Energy Production	Luciferase	MMSR116_RS22605	LLM class flavin-dependent oxidoreductase	YHBW_ECOL6 ame: Full=Luciferase-like monooxygenase	1.827202051	342	5	4.16E-79	55.8%	1	C:cytosol			G3DSA:3.40.50.1980 (GENE3D); G3DSA:3.40.50.1980 (GENE3D); IPR002491 (PFAM); PTHR30535 (PANTHER); IPR002491 (PROSITE_PROFILES); SSF53807 (SUPERFAMILY)
	Energy Production	Regulation of macromolecule metabolic process - Luciferase	MMSR116_RS04130	SMP-30/gluconolactonase/LRE family protein	GALAB_RHIME ame: Full= sugar lactone lactonase	2.244206328	296	5	5.18E-88	48.8%	8	P:positive regulation of hydrolase activity; P:regulation of macromolecule metabolic process; F:metal ion binding; C:intracellular part; P:single-organism process; P:positive regulation of biological process; P:regulation of primary metabolic process; P:negative regulation of cellular metabolic process			IPR025668 (PFAM); IPR008490 (PFAM); PTHR33408:SF2 (PANTHER); PTHR33408 (PANTHER)
	Energy Production		MMSR116_RS06605	GNAT family N-acetyltransferase	---NA---	1.515096631	197								IPR001155 (PFAM); IPR013785

															(G3DSA:3.20.20. GENE3D); PTHR43303 (PANTHER); SSF51395 (SUPERFAMILY)
	Energy Production		MMSR116_RS13970	GNAT family N-acetyltransferase	---NA---	-1.210119424	394							EC:1.5 .1.20	G3DSA:3.20.20. 220 (GENE3D); IPR003171 (PFAM); IPR004620 (TIGRFAM); PTHR21091 (PANTHER); PTHR21091:SF1 63 (PANTHER); IPR029041 (SUPERFAMILY)
	Energy Production		MMSR116_RS20115	N-acetyltransferase	---NA---	1.552500684	182								IPR029303 (PFAM); PF13440 (PFAM); PTHR30250 (PANTHER); PTHR30250:SF1 0 (PANTHER)
	Energy Production	PHA	MMSR116_RS04820	polyhydroxyalkanoate depolymerase (PHA)	PHAC_ALLVD ame: Full=Poly(3-hydroxyalkanoate) polymerase subunit Short=PHA polymerase ame: Full=PHB synthase subunit ame: Full=Poly(hydroxyalkanoic acid) synthase subunit Short=PHA synthase subunit Short=Polyhydroxyalkanoic acid synthase ame: Full=Poly-beta-hydroxybutyrate polymerase Short=PHB polymerase Short=Poly(3-hydroxybutyrate) polymerase	1.151720285	425	1	1.06E-3	38%	3	F:transferase activity, transferring acyl groups; P:poly-hydroxybutyrate biosynthetic process; F:transferase activity		EC:1.4 .1.2	IPR028971 (PFAM); IPR007780 (PIRSF); IPR007780 (PANTHER); SSF53223 (SUPERFAMILY); IPR036291 (SUPERFAMILY)
	Energy Production	tricarboxylic acid cycle	MMSR116_RS20380	succinate--CoA ligase subunit alpha	SUCD_RICBR ame: Full=Succinate-- ligase	0.925372998	294	5	5.75E-123	82%	14	P:nucleoside triphosphate biosynthetic process; P:tricarboxylic acid cycle; P:succinate metabolic process; C:mitochondrial inner membrane; C:plasma membrane; F:succinate-CoA ligase (ADP-forming) activity; P:succinyl-CoA metabolic process; F:succinate-CoA ligase (GDP-forming) activity; F:ATP binding; C:extracellular exosome; C:succinate-CoA ligase complex (GDP-forming); F:protein heterodimerization activity; F:cofactor binding; F:GDP binding			IPR035903 (G3DSA:2.60.30 0.GENE3D); IPR016092 (TIGRFAM); IPR000361 (PFAM); PTHR43011 (PANTHER); IPR035903 (SUPERFAMILY)
	Energy Production	tricarboxylic acid cycle	MMSR116_RS20385	2-oxoglutarate dehydrogenase E1 component	ODO1_OCHA4 ame: Full=2-oxoglutarate dehydrogenase E1 component ame: Full=Alpha-ketoglutarate dehydrogenase	1.28436805	995	5	0.00E+00	82%	4	F:thiamine pyrophosphate binding; F:oxoglutarate dehydrogenase (succinyl-transferring) activity; P:glycolytic process; P:tricarboxylic acid cycle			no IPS match
	Energy Production	tricarboxylic acid cycle	MMSR116_RS21750	aspartate aminotransferase family protein	GATP1_SOLLC ame: Full=Gamma aminobutyrate transaminase mitochondrial ame: Full=Gamma-aminobutyrate transaminase isozyme 1 Short= -TP1 Short= -T1 Flags: Precursor	0.948929921	462	5	2.69E-141	66.4%	4	F:catalytic activity; C:intracellular membrane-bounded organelle; C:cytoplasmic part; F:binding			G3DSA:3.90.101 0.10 (GENE3D); IPR002871 (PFAM); PTHR10093:SF1 (PANTHER); IPR002871 (PANTHER); SSF82649 (SUPERFAMILY)
	Energy Production	biosynthesis of polysaccharides	MMSR116_RS25665	UDP-glucose/GDP-mannose dehydrogenase family	UDG_RHIME ame: Full=UDP-glucose 6-dehydrogenase Short=UDP-Glc dehydrogenase Short=UDP- Short=UDPGDH	1.108628438	447	5	0.00E+00	68%	5	F:NAD binding; P:UDP-glucuronate biosynthetic process; F:UDP-glucose 6-dehydrogenase activity; P:oxidation-reduction process; P:capsule polysaccharide biosynthetic process			IPR001638 (SMART); G3DSA:3.40.190 .10 (GENE3D); G3DSA:3.40.190 .10 (GENE3D); IPR001638 (PFAM); PTHR44475:SF2 (PANTHER); PTHR44475

															(PANTHER); IPR006311 (PROSITE_PROFILES); SSF53850 (SUPERFAMILY)
	Energy Production	Glycerophospholipid metabolism	MMSR116_RS28550	glycerophosphoryl diester phosphodiesterase	A4177_ARTBC ame: Full=Extracellular ARB_04177	1.251883106	408	5	5.02E-87	46.4%	14	P:glycerol metabolic process; C:cell outer membrane; C:periplasmic space; C:extracellular region; F:glycerophosphodiester phosphodiesterase activity; C:vacuole; C:plasma membrane; F:metal ion binding; F:calcium ion binding; C:cell wall; P:lipid metabolic process; C:membrane; F:phosphoric diester hydrolase activity; F:hydrolase activity			IPR010827 (PFAM); G3DSA:3.10.20.310 (GENE3D); IPR023707 (PIRSF); G3DSA:2.40.160.50 (GENE3D); G3DSA:2.40.160.50 (GENE3D); G3DSA:3.10.20.310 (GENE3D); IPR023707 (TIGRFAM); G3DSA:3.10.20.310 (GENE3D); IPR000184 (PFAM); G3DSA:3.10.20.310 (GENE3D); G3DSA:3.10.20.310 (GENE3D); IPR023707 (PTHR12815:PANTHER); PTHR12815 (PANTHER); IPR023707 (HAMAP); IPR034746 (PROSITE_PROFILES)
	Energy Production	fatty acid byossintesis	MMSR116_RS06645	beta-ketoacyl-[acyl-carrier-protein] synthase I	FABB_PSEAB ame: Full=3-oxoacyl-[acyl-carrier-] synthase 1 ame: Full=3-oxoacyl-	0.9220399	408	5	2.78E-165	71.6%	3	F:3-oxoacyl-[acyl-carrier-protein] synthase activity; C:cytosol; P:fatty acid biosynthetic process		EC:6.2.1.5; EC:6.2.1.4	PR01798 (PRINTS); IPR003781 (SMART); IPR005811 (PFAM); G3DSA:3.40.50.720 (GENE3D); IPR016102 (G3DSA:3.40.50.GENE3D); IPR005810 (PIRSF); IPR003781 (PFAM); IPR005810 (TIGRFAM); PTHR11117:SF2 (PANTHER); PTHR11117 (PANTHER); IPR005810 (HAMAP); IPR016102 (SUPERFAMILY); IPR036291 (SUPERFAMILY)
	Energy Production	fatty acid	MMSR116_RS03285	acyl-CoA dehydrogenase	GCDH_CAEEL ame: Full=Probable glutaryl-mitochondrial Short=GCD Flags: Precursor	0.958418912	409	5	2.53E-179	78%	12	P:fatty-acyl-CoA biosynthetic process; P:fatty acid beta-oxidation using acyl-CoA dehydrogenase; C:mitochondrial matrix; F:fatty-acyl-CoA binding; F:flavin adenine dinucleotide binding; F:electron carrier activity; F:oxidoreductase activity, acting on the CH-CH group of donors, with a flavin as acceptor; P:lipid homeostasis; F:glutaryl-CoA dehydrogenase activity; P:tryptophan metabolic process; C:mitochondrial inner membrane; P:lysine catabolic process			IPR001650 (SMART); IPR014001 (SMART); G3DSA:3.40.50.300 (GENE3D); G3DSA:3.40.50.300 (GENE3D); IPR001650 (PFAM); IPR005580 (PFAM);

															IPR011545 (PFAM); PTHR24031:SF349 (PANTHER); PTHR24031 (PANTHER); IPR014001 (PROSITE_PROFILES); IPR001650 (PROSITE_PROFILES); IPR014014 (PROSITE_PROFILES); IPR027417 (SUPERFAMILY)
	Energy Production	amino acid	MMSR116_RS25785	glycine amidinotransferase	GATM_DANRE ame: Full=Glycine mitochondrial ame: Full=L-arginine:glycine amidinotransferase ame: Full=Transamidinase Flags: Precursor	-1.05009332	391	5	2.21E-105	59.6%	3	C:mitochondrion; P:response to chemical; F:transferase activity			G3DSA:1.10.287.500 (GENE3D); IPR007439 (PFAM); SSF75708 (SUPERFAMILY)
	Energy Production	aminoacid	MMSR116_RS24330	type III PLP-dependent enzyme	DCLO_SELRU ame: Full=Lysine ornithine decarboxylase Short=LDC	-1.379853674	388	5	1.99E-57	49%	2	F:lyase activity; P:polyamine biosynthetic process		EC:2.5.1.7	IPR001986 (PFAM); IPR005750 (TIGRFAM); IPR036968 (G3DSA:3.65.10.GENE3D); IPR036968 (G3DSA:3.65.10.GENE3D); PTHR43783 (PANTHER); IPR005750 (HAMAP); IPR013792 (SUPERFAMILY)
	Energy Production	L-homocysteine biosynthesis	MMSR116_RS19390	adenosylhomocysteinase	SAHH_METEP ame: Full=Adenosylhomocysteinase ame: Full=S-adenosyl-L-homocysteine hydrolase Short=cyase	1.407637682	466	5	0.00E+00	95.8%	3	C:cytoplasm; F:adenosylhomocysteinase activity; P:one-carbon metabolic process			IPR000522 (PFAM); IPR037294 (G3DSA:1.10.3470.GENE3D); IPR000522 (PANTHER); PTHR30472:SF3 (PANTHER); IPR037294 (SUPERFAMILY)
	Energy Production	L- Lysine degradation via actate pathway	MMSR116_RS29585	3-keto-5-aminohexanoate cleavage protein	KCE_FUSNN ame: Full=3-keto-5-aminohexanoate cleavage enzyme	1.124546966	280	3	3.67E-50	54%	4	F:catalytic activity; F:metal ion binding; P:L-lysine catabolic process to acetate; F:transferase activity			IPR000644 (SMART); G3DSA:3.10.580.10 (GENE3D); IPR000644 (PFAM); PTHR43080:SF2 (PANTHER); PTHR43080 (PANTHER); IPR000644 (PROSITE_PROFILES); SSF54631 (SUPERFAMILY)
	Energy Production	amino acid	MMSR116_RS09695	aspartate ammonia-lyase	ASPA_PSEFL ame: Full=Aspartate ammonia-lyase Short=Aspartase	1.232968393	514	5	0.00E+00	70.4%	8	P:malate metabolic process; P:protein tetramerization; F:aspartate ammonia-lyase activity; C:cytosol; P:aspartate metabolic process; P:tricarboxylic acid cycle; P:cellular amino acid biosynthetic process; P:fumarate metabolic process		EC:2.5.1.18	IPR004045 (PFAM); G3DSA:1.20.1050.10 (GENE3D); G3DSA:3.40.30.10 (GENE3D); IPR004046 (PFAM); PTHR44051:SF1 (PANTHER); PTHR44051 (PANTHER); IPR004045 (PROSITE_PROFILES); IPR010987

															(PROSITE_PROFILES); IPR036282 (SUPERFAMILY); IPR036249 (SUPERFAMILY)
	Energy Production	amino acid	MMSR116_RS07215	aspartate-semialdehyde dehydrogenase	DHAS_AQUAE ame: Full=Aspartate-semialdehyde dehydrogenase Short=ASA dehydrogenase Short=ASADH ame: Full=Aspartate-beta-semialdehyde dehydrogenase	0.950720408	345	5	2.68E-133	68.2%	6	F:nucleotide binding; P:methionine biosynthetic process; P:lysine biosynthetic process; F:coenzyme binding; P:diaminopimelate metabolic process; F:oxidoreductase activity, acting on the aldehyde or oxo group of donors, NAD or NADP as acceptor			no IPS match
	Energy Production	amino acid	MMSR116_RS01745	D-amino acid dehydrogenase	DADA3_RHILO ame: Full=D-amino acid dehydrogenase 3	1.202565188	420	5	0.00E+00	58%	2	F:oxidoreductase activity; P:D-amino acid catabolic process			IPR014440 (PIRSF); G3DSA:3.40.30.10 (GENE3D); IPR001853 (PFAM); PTHR42943:SF2 (PANTHER); PTHR42943 (PANTHER); IPR036249 (SUPERFAMILY)
	Energy Production	amino acid degradation	MMSR116_RS25555	methylmalonate-semialdehyde dehydrogenase (CoA acylating)	BAUC_PSEAE ame: Full= 3-oxopropanoate dehydrogenase	0.910031492	498	5	0.00E+00	67%	12	F:aldehyde dehydrogenase (NAD) activity; P:response to oxidative stress; P:brown fat cell differentiation; C:extracellular exosome; F:copper ion binding; P:beta-alanine biosynthetic process; C:mitochondrial matrix; F:methylmalonate-semialdehyde dehydrogenase (acylating) activity; P:valine catabolic process; P:thymine catabolic process; F:malonate-semialdehyde dehydrogenase (acetylating) activity; C:nucleoplasm			PF13515 (PFAM); IPR032692 (PFAM)
	Energy Production	amino acid - serine	MMSR116_RS11755	D-serine ammonia-lyase	SDHD_PSEP1 ame: Full=Probable D-serine dehydratase ame: Full=D-serine deaminase Short=DSD	1.132621646	429	5	1.37E-91	60%	3	F:lyase activity; P:serine family amino acid metabolic process; P:D-amino acid metabolic process			IPR002347 (PRINTS); IPR002347 (PRINTS); PF13561 (PFAM); G3DSA:3.40.50.720 (GENE3D); PTHR42898:SF8 (PANTHER); PTHR42898 (PANTHER); IPR036291 (SUPERFAMILY)
	Energy Production	protease	MMSR116_RS01620	serine protease	DEGPL_CHLTR ame: Full=Probable periplasmic serine endoprotease -like ame: Full=59 kDa immunogenic ame: Full=Protease Do ame: Full=SK59 Flags: Precursor	1.156233945	324	5	1.71E-10	47.6%	10	P:proteolysis; C:membrane; C:integral component of membrane; F:serine-type endopeptidase activity; F:serine-type peptidase activity; C:outer membrane-bounded periplasmic space; C:cell outer membrane; F:peptidase activity; C:periplasmic space; F:hydrolase activity			IPR004046 (PFAM); G3DSA:1.20.1050.10 (GENE3D); G3DSA:3.40.30.10 (GENE3D); IPR004045 (PFAM); PTHR44051 (PANTHER); PTHR44051:SF1 (PANTHER); IPR010987 (PROSITE_PROFILES); IPR004045 (PROSITE_PROFILES); IPR036249 (SUPERFAMILY); IPR036282 (SUPERFAMILY)
	Energy Production	amino acid - serine	MMSR116_RS25615	serine O-acetyltransferase	CYSE_SALTY ame: Full=Serine acetyltransferase Short=SAT ame: Full=Serine transacetylase	1.032283522	283	5	6.05E-97	69.2%	5	F:serine O-acetyltransferase activity; C:cytosol; C:cysteine synthase complex; P:sulfate assimilation; P:cysteine biosynthetic process from serine			IPR018077 (SMART); IPR002053 (PFAM); G3DSA:3.20.20.80 (GENE3D); PTHR34135:SF2 (PANTHER); PTHR34135 (PANTHER);

															IPR017853 (SUPERFAMILY)
	Energy Production	amino acid - serine	MMSR116_RS24210	PrkA family serine protein kinase	PRKA_BACSU ame: Full=	1.685977014	650	3	0.00E+00	62%	2	F:protein kinase activity; P:protein phosphorylation			IPR020013 (TIGRFAM); IPR010930 (PFAM); IPR001444 (PFAM); PTHR30435:SF1 (PANTHER); PTHR30435 (PANTHER); IPR037925 (SUPERFAMILY)
	Energy Production	amino acid	MMSR116_RS21385	hypothetical protein	PKN1_CHLPN ame: Full=Serine threonine- kinase pkn1	2.033602624	595	5	7.45E-15	42.4%	14	F:protein homodimerization activity; C:endoplasmic reticulum; F:protein kinase activity; F:protein serine/threonine kinase activity; P:protein phosphorylation; F:nucleotide binding; C:endoplasmic reticulum lumen; F:ATP binding; F:oxidoreductase activity; F:metal ion binding; P:oxidation-reduction process; P:phosphorylation; F:transferase activity; F:kinase activity			G3DSA:3.40.162 0.10 (GENE3D); TIGR01552 (TIGRFAM); IPR006442 (PFAM); IPR036165 (SUPERFAMILY)
		purine metabolism.	MMSR116_RS22755	xanthine dehydrogenase family protein	IORB_BREDI ame: Full=Isoquinoline 1-oxidoreductase subunit beta	1.348806998	731	5	6.59E-88	42.8%	4	P:regulation of cellular process; F:oxidoreductase activity; F:binding; P:negative regulation of biological process		EC:2.7 .1.69	IPR036662 (G3DSA:3.40.50. GENE3D); IPR004701 (PFAM); PTHR33799 (PANTHER); PTHR33799:SF1 (PANTHER); IPR004701 (PROSITE_PROFI LES); IPR036662 (SUPERFAMILY)
		pyrimidine metabolism.	MMSR116_RS29495	hypothetical protein	CODA_ECOLI ame: Full=Cytosine deaminase Short=CD Short=CDA Short=CDase ame: Full=Cytosine aminohydrolase ame: Full=Isoguanine deaminase	1.199115381	410	5	2.08E-53	45.2%	9	P:cellular nitrogen compound catabolic process; P:aromatic compound catabolic process; F:transition metal ion binding; P:organic cyclic compound catabolic process; F:hydrolase activity, acting on carbon-nitrogen (but not peptide) bonds, in cyclic amidines; C:cytoplasm; P:organonitrogen compound catabolic process; P:heterocycle catabolic process; F:deaminase activity			no IPS match
		pyrimidine metabolism.	MMSR116_RS09635	NUDIX domain-containing protein	ADPP_METJA ame: Full=ADP-ribose pyrophosphatase ame: Full=ADP-ribose diphosphatase ame: Full=ADP-ribose phosphohydrolase ame: Full=Adenosine diphosphoribose pyrophosphatase Short=ADPR-PPase	1.888358904	243	1	8.32E-6	51%	3	F:ADP-ribose diphosphatase activity; F:metal ion binding; F:hydrolase activity			no IPS match
		biosynthesis of the pyrimidine	MMSR116_RS15475	NMT1/THI5-like domain-containing protein	SSUA_ECOLI ame: Full= aliphatic sulfonates-binding Flags: Precursor	2.096549284	316	5	8.65E-7	47.8%	10	C:ATP-binding cassette (ABC) transporter complex, substrate-binding subunit-containing; P:sulfur compound metabolic process; F:alkanesulfonate transporter activity; C:membrane; P:alkanesulfonate transport; C:periplasmic space; P:transport; P:thiamine diphosphate biosynthetic process; C:plasma membrane; P:thiamine biosynthetic process			G3DSA:3.40.630 .30 (GENE3D); IPR007434 (PFAM); PTHR31384:SF4 2 (PANTHER); PTHR31384 (PANTHER); IPR016181 (SUPERFAMILY)
		stress reponse	MMSR116_RS14700	polyphosphate kinase 2	PK21C_RHIME ame: Full=Polyphosphate:ADP phosphotransferase 3 ame: Full=Polyphosphate kinase PPK2 3	1.887535652	280	5	4.34E-108	72.2%	2	P:phosphorylation; F:polyphosphate kinase activity			no IPS match
Cell division			MMSR116_RS28465	hypothetical protein	SLMA_SHEB5 ame: Full=Nucleoid occlusion factor	1.651132292	242	5	3.3E-2	48.4%	9	C:nucleoid; P:cell division; P:regulation of cell division; F:DNA binding; C:bacterial nucleoid; C:cytoplasm; P:negative regulation of barrier septum assembly; F:sequence-specific DNA binding; P:cell cycle			IPR001789 (SMART); IPR001789 (PFAM); G3DSA:3.40.50. 2300 (GENE3D); PTHR43228 (PANTHER); IPR001789

															(PROSITE_PROFILES); IPR011006 (SUPERFAMILY)
			MMSR116_RS07170	tetratricopeptide repeat protein	Y3534_RHIEC ame: Full=TPR repeat-containing Flags: Precursor	1.09925917	203	5	9.03E-20	51%	29	P:cell proliferation; C:nucleoplasm; F:protein complex scaffold; P:negative regulation of transcription, DNA-templated; C:intracellular membrane-bounded organelle; C:cytosol; P:metaphase/anaphase transition of mitotic cell cycle; P:protein K11-linked ubiquitination; F:protein phosphatase binding; C:anaphase-promoting complex; C:endoplasmic reticulum; P:defense response to bacterium; P:protein ubiquitination involved in ubiquitin-dependent protein catabolic process; P:defense response to bacterium, incompatible interaction; C:spindle microtubule; C:nucleus; P:proteasome-mediated ubiquitin-dependent protein catabolic process; C:centrosome; C:perinuclear region of cytoplasm; F:protein binding; C:cytoplasm; C:integral component of membrane; C:spindle; P:anaphase-promoting complex-dependent catabolic process; P:regulation of ubiquitin-protein ligase activity involved in mitotic cell cycle; P:positive regulation of ubiquitin-protein ligase activity involved in regulation of mitotic cell cycle transition; P:negative regulation of defense response; P:protein ubiquitination; P:negative regulation of ubiquitin-protein ligase activity involved in mitotic cell cycle			no IPS match
			MMSR116_RS12015	cell division topological specificity factor	MINE_METRJ ame: Full=Cell division topological specificity factor	-1.478018814	112	5	2.19E-48	84.2%	3	P:cell division; P:regulation of barrier septum assembly; P:cell cycle			IPR001867 (SMART); IPR001789 (SMART); IPR001867 (PFAM); IPR036388 (G3DSA:1.10.10. GENE3D); G3DSA:3.40.50. 2300 (GENE3D); IPR001789 (PFAM); PTHR26402:SF7 19 (PANTHER); PTHR26402 (PANTHER); IPR001867 (PROSITE_PROFILES); IPR001789 (PROSITE_PROFILES); IPR011006 (SUPERFAMILY); IPR016032 (SUPERFAMILY)
			MMSR116_RS19700	cell division protein FtsA	FTSA_AGRFC ame: Full=Cell division	-1.208080745	440	5	1.23E-146	59.6%	4	P:cell division; F:protein binding; C:plasma membrane; P:cell cycle			G3DSA:1.10.321 0.10 (GENE3D); IPR003607 (PFAM); PTHR21262:SF2 7 (PANTHER); PTHR21262 (PANTHER); SSF109604 (SUPERFAMILY)
			MMSR116_RS30440	hypothetical protein	NUF2_RAT ame: Full=Kinetochore Nuf2 ame: Full=Cell division cycle-associated 1	-1.473068699	163	2	3.53E-5	54%	8	P:cell division; C:nucleus; C:Ndc80 complex; C:membrane; P:mitotic nuclear division; C:condensed chromosome kinetochore; P:transport; P:protein secretion			IPR000182 (PFAM); G3DSA:3.40.630 .30 (GENE3D); IPR000182 (PROSITE_PROFILES); IPR016181 (SUPERFAMILY)
			MMSR116_RS30380	UDP-N-acetylglucosamine 1-carboxyvinyltransferase	MURA_METRJ ame: Full=UDP-N-acetylglucosamine 1-carboxyvinyltransferase ame: Full=Enoylpyruvate transferase ame: Full=UDP-N-acetylglucosamine enolpyruvyl transferase Short=EPT	-1.678445869	429	5	0.00E+00	94.6%	8	P:regulation of cell shape; P:cell division; F:UDP-N-acetylglucosamine 1-carboxyvinyltransferase activity; C:cytoplasm; P:peptidoglycan biosynthetic process; P:UDP-N-acetylgalactosamine biosynthetic process; P:cell wall organization; P:cell cycle			no IPS match

			MMSR116_RS12020	septum site-determining protein MinD	MIND_NEIMB ame: Full=Septum site-determining ame: Full=Cell division inhibitor	-1.514414859	271	5	1.07E-90	74%	10	C:cell pole; F:ATPase activity; P:cell division; F:ATP binding; F:identical protein binding; P:negative regulation of cell division; C:cytosol; P:chromosome segregation; C:intrinsic component of plasma membrane; C:cytoplasmic side of plasma membrane		IPR007627 (PFAM); G3DSA:1.10.1740.10 (GENE3D); IPR036388 (G3DSA:1.10.10. GENE3D); IPR014284 (TIGRFAM); IPR013249 (PFAM); PTHR43133 (PANTHER); IPR013325 (SUPERFAMILY); IPR013324 (SUPERFAMILY)
		cell division protease	MMSR116_RS05195	ATP-dependent metallopeptidase FtsH/Yme1/Tma	FTSH_BARBK ame: Full=ATP-dependent zinc metalloprotease	1.138006667	640	5	0.00E+00	78%	10	F:ATPase activity; P:cell division; F:ATP binding; F:zinc ion binding; P:protein catabolic process; P:proteolysis; C:integral component of membrane; F:metalloendopeptidase activity; C:plasma membrane; P:cell cycle		IPR025187 (PFAM); IPR025187 (PANTHER)
		bacterial cell wall biosynthesis	MMSR116_RS21235	serine-type D-Ala-D-Ala carboxypeptidase	DACA_HAEIN ame: Full=D-alanyl-D-alanine carboxypeptidase Short=DD-carboxypeptidase Short=DD-peptidase ame: Full=Penicillin-binding 5 Short=PBP-5 Flags: Precursor	-1.019354872	505	5	3.71E-43	55.4%	6	P:regulation of cell shape; C:integral component of plasma membrane; F:carboxypeptidase activity; P:response to drug; P:peptidoglycan biosynthetic process; F:penicillin binding		IPR004401 (TIGRFAM); IPR004401 (PIRSF); IPR036894 (G3DSA:3.30.1310.GENE3D); IPR004401 (PFAM); PTHR33449:SF1 (PANTHER); IPR004401 (PANTHER); IPR004401 (HAMAP); IPR036894 (SUPERFAMILY)
		bacterial cell wall biosynthesis	MMSR116_RS10030	L,D-transpeptidase	ERFK_ECOLI ame: Full=Probable L,D-transpeptidase Flags: Precursor	-1.227967683	221	5	7.5E-19	50.8%	6	F:transferase activity, transferring amino-acyl groups; P:proteolysis; P:Gram-negative-bacterium-type cell wall biogenesis; F:cysteine-type carboxypeptidase activity; F:peptidoglycan L,D-transpeptidase activity; P:peptidoglycan-protein cross-linking		IPR000534 (SMART); IPR005986 (TIGRFAM); PIRSF000148 (PIRSF); IPR000534 (PFAM); IPR012280 (PFAM); G3DSA:3.40.50.720 (GENE3D); G3DSA:3.30.360.10 (GENE3D); PTHR10174:SF2 (PANTHER); PTHR10174 (PANTHER); IPR012080 (HAMAP); SSF55347 (SUPERFAMILY); IPR036291 (SUPERFAMILY)
		rod shape	MMSR116_RS02070	septal ring lytic transglycosylase RlpA family	RLPA_PSEAB ame: Full=Endolytic peptidoglycan transglycosylase ame: Full=Rare lipo A Flags: Precursor	-1.915341742	450	5	1.97E-37	68.6%	1	C:membrane		no IPS match
			MMSR116_RS19830	N-acetyltransferase	PUR2_STRP1 ame: Full=Phosphoribosylamine--glycine ligase ame: Full=GARS ame: Full=Glycinamide ribonucleotide synthetase ame: Full=Phosphoribosylglycinamide synthetase	1.215352741	427	2	9.21E-2	43%	11	F:nucleotide binding; F:magnesium ion binding; F:ATP binding; F:phosphoribosylamine-glycine ligase activity; F:catalytic activity; F:metal ion binding; P:purine nucleotide biosynthetic process; P:'de novo' IMP biosynthetic process; F:manganese ion binding; P:purine nucleobase biosynthetic process; F:ligase activity		G3DSA:1.10.357.10 (GENE3D); IPR009057 (SUPERFAMILY)

Transport		K+ channels	MMSR116_RS24965	mechanosensitive ion channel family protein	---NA---	1.174333667	374								no IPS match
		Anion transporter	MMSR116_RS02620	oxalate/formate MFS antiporter	OXLT_OXAFO ame: Full=Oxalate:formate antiporter Short=OFA ame: Full=Oxalate:formate antiport ame: Full=Oxalate:formate exchange	1.004986847	425	5	4.22E-61	45%	3	C:membrane; C:cell part; P:transport			no IPS match
		anion transporters	MMSR116_RS21710	STAS/SEC14 domain-containing protein	---NA---	1.11312872	120								no IPS match
		ion transport	MMSR116_RS16110	citrate transporter	MEMP_CUPNH ame: Full=Membrane	2.649028173	432	1	3.51E-10	46%	6	P:sodium ion transport; P:transmembrane transport; C:membrane; C:integral component of membrane; F:transporter activity; C:plasma membrane			no IPS match
		inorganic anion uptake transporters	MMSR116_RS01695	SulP family inorganic anion transporter	YVDB_BACSU ame: Full= sulfate transporter	1.285344313	516	2	1.12E-3	59.5%	17	P:sulfate transport; P:L-aspartate transmembrane transport; P:fumarate transport; F:secondary active sulfate transmembrane transporter activity; P:transmembrane transport; F:succinate transmembrane transporter activity; P:sulfate transmembrane transport; F:L-aspartate transmembrane transporter activity; P:transport; C:plasma membrane; C:integral component of plasma membrane; C:membrane; C:integral component of membrane; P:succinate transmembrane transport; P:aspartate transport; F:fumarate transmembrane transporter activity; F:sulfate transmembrane transporter activity			IPR006698 (PFAM); IPR006698 (PANTHER); IPR006698 (HAMAP)
		sulfate exporter	MMSR116_RS08495	putative sulfate exporter family transporter	Y3189_BRADU ame: Full=UPF0324 membrane blr3189	1.44770817	362	5	8.49E-96	73.4%	1	C:membrane			IPR019284 (PFAM)
		sugars, drugs, metabolites, oligosaccharides, amino acids	MMSR116_RS22290	MFS transporter	MMLH_CUPPJ ame: Full=Probable 4-methylmuconolactone transporter	1.0875658	434	5	2.72E-82	51.2%	2	P:transport; F:transporter activity			IPR014025 (PRINTS); IPR011900 (TIGRFAM); IPR002109 (PFAM); G3DSA:3.40.30.10 (GENE3D); PTHR10168:SF180 (PANTHER); PTHR10168 (PANTHER); IPR002109 (PROSITE_PROFILES); IPR036249 (SUPERFAMILY)
			MMSR116_RS27205	MFS transporter	YUSP_BACSU ame: Full=Uncharacterized MFS-type transporter	1.368803508	490	5	1.78E-60	49.8%	7	P:transmembrane transport; C:membrane; C:integral component of membrane; P:response to antibiotic; P:transport; C:plasma membrane; F:transporter activity			IPR008258 (PFAM); G3DSA:1.10.530.10 (GENE3D); IPR023346 (SUPERFAMILY)
			MMSR116_RS21120	MFS transporter	YQJV_BACSU ame: Full=Uncharacterized MFS-type transporter	1.63659724	398	3	1.36E-3	47.33%	8	P:transmembrane transport; C:membrane; C:integral component of membrane; P:aldonate transport; P:transport; F:organic anion transmembrane transporter activity; C:plasma membrane; F:transporter activity			no IPS match
			MMSR116_RS07435	MFS transporter	YDHP_ECOLI ame: Full=Inner membrane transport	1.284195567	391	5	2.51E-52	58.4%	1	C:plasma membrane			IPR007833 (PFAM)
		carbohydrate transport uptake	MMSR116_RS15270	PTS fructose transporter subunit IIA	PTNAB_ECOLI ame: Full=PTS system mannose-specific EIIAB component ame: Full=EIIAB-Man ame: Full=EIII-Man Includes: ame: Full=Mannose-specific phosphotransferase enzyme IIA component ame: Full=PTS system mannose-specific EIIA component Includes: ame: Full=Mannose-specific phosphotransferase enzyme IIB component ame: Full=PTS system mannose-specific EIIB component	1.275389799	133	5	6.94E-11	51.4%	4	C:cytoplasm; C:integral component of membrane; F:protein-N(PI)-phosphohistidine-sugar phosphotransferase activity; P:hexose transport		EC:1.8.4.11	IPR002569 (PFAM); IPR002569 (TIGRFAM); IPR036509 (G3DSA:3.30.1060.GENE3D); PTHR42799:SF2 (PANTHER); PTHR42799 (PANTHER); IPR002569 (HAMAP); IPR036509 (SUPERFAMILY)

		facilitates protein transport	MMSR116_RS16730	nuclear transport factor 2 family protein	---NA---	1.449285575	143								no IPS match
		amino acid	MMSR116_RS20220	dicarboxylate/amino acid:cation symporter	DCTA_GEOBB ame: Full=C4-dicarboxylate transport	1.272086242	425	5	1.54E-76	61%	2	P:dicarboxylic acid transport; C:membrane			IPR011780 (TIGRFAM); G3DSA:3.40.50.1100 (GENE3D); G3DSA:3.40.50.1100 (GENE3D); IPR001926 (PFAM); PTHR43419 (PANTHER); IPR011780 (PTHR43419:PANTHER); IPR011780 (HAMAP); IPR036052 (SUPERFAMILY)
		amino acid	MMSR116_RS15440	dicarboxylate/amino acid:cation symporter	DCTA_OCHA4 ame: Full=C4-dicarboxylate transport	-1.609513405	447	5	0.00E+00	85.2%	5	P:dicarboxylic acid transport; P:transmembrane transport; C:integral component of membrane; F:symporter activity; C:plasma membrane			IPR036707 (G3DSA:3.30.1070.GENE3D); IPR005527 (TIGRFAM); IPR005527 (PFAM); IPR005527 (PANTHER); PTHR33404:SF2 (PANTHER); IPR005527 (HAMAP); IPR036707 (SUPERFAMILY)
		amino acid - leucine	MMSR116_RS29515	ABC transporter substrate-binding protein	LIVK_ECOLI ame: Full=Leucine-specific-binding Short=L-BP Short=LS-BP Flags: Precursor	1.549732529	393	5	1.38E-14	43.2%	1	P:amino acid transport			IPR017102 (PIRSF); IPR014710 (G3DSA:2.60.120.GENE3D); IPR013096 (PFAM); IPR011051 (SUPERFAMILY)
		amino acid - glutamine	MMSR116_RS29475	ABC transporter substrate-binding protein	GLNH_BACSU ame: Full=ABC transporter glutamine-binding Flags: Precursor	1.119526118	279	5	1.02E-19	47.6%	9	C:membrane; C:periplasmic space; P:ionotropic glutamate receptor signaling pathway; F:ionotropic glutamate receptor activity; P:transport; P:amino acid transport; C:plasma membrane; F:transporter activity; C:cell surface			IPR021252 (PFAM)
		cobalamin - vit B (Co+N)	MMSR116_RS08125	ABC transporter substrate-binding protein	BTUFA_HALSA ame: Full=Cobalamin-binding ame: Full=Vitamin B12-binding Flags: Precursor	-0.98222713	277	5	2.97E-10	42.6%	8	F:cobalamin binding; C:membrane; F:small molecule binding; C:outer membrane-bounded periplasmic space; C:periplasmic space; P:cobalamin transport; P:transport; C:plasma membrane			IPR036388 (G3DSA:1.10.10.GENE3D); G3DSA:3.90.79.10 (GENE3D); IPR000086 (PFAM); PTHR22769 (PANTHER); PTHR22769:SF59 (PANTHER); IPR036390 (SUPERFAMILY); IPR015797 (SUPERFAMILY)
		sulfate importer	MMSR116_RS22370	sulfate ABC transporter substrate-binding protein	SUBI_ECOLI ame: Full=Sulfate-binding ame: Full=Sulfate starvation-induced 2 Short=SSI2 Flags: Precursor	-1.344937858	345	5	1.4E-115	65%	4	P:sulfur compound metabolic process; F:sulfate binding; C:outer membrane-bounded periplasmic space; P:transport			IPR007390 (PFAM); IPR007390 (PANTHER)
		hemine (Fe)	MMSR116_RS08115	ABC transporter ATP-binding protein	HMUV_PSEPK ame: Full=Hemin import ATP-binding	-1.165754767	271	5	1.21E-45	58%	3	F:ATPase activity, coupled to transmembrane movement of substances; P:transport; C:plasma membrane		EC:3.4.24; EC:3.6.1.3	IPR003593 (SMART); IPR000642 (PFAM); G3DSA:2.40.50.920 (GENE3D);

															G3DSA:1.10.8.60 (GENE3D); G3DSA:3.40.50.300 (GENE3D); IPR005936 (TIGRFAM); G3DSA:1.20.58.760 (GENE3D); IPR011546 (PFAM); IPR003959 (PFAM); PTHR23076:SF93 (PANTHER); PTHR23076 (PANTHER); IPR005936 (HAMAP); IPR027417 (SUPERFAMILY); IPR037219 (SUPERFAMILY)
		iron importer	MMSR116_RS08120	iron ABC transporter permease	YVRB_BACSU ame: Full=Uncharacterized ABC transporter permease	-1.125429077	348	5	9.46E-9	60.2%	9	P:iron ion homeostasis; F:cobalamin transporter activity; P:ion transport; C:membrane; C:integral component of membrane; P:cobalamin transport; P:transport; C:plasma membrane; F:transporter activity			IPR020471 (PRINTS); IPR023210 (PFAM); IPR036812 (G3DSA:3.20.20.GENE3D); IPR020471 (PIRSF); PTHR43827:SF3 (PANTHER); PTHR43827 (PANTHER); IPR036812 (SUPERFAMILY)
			MMSR116_RS28065	TonB-dependent siderophore receptor	FCT_DICD3 ame: Full=Ferrichrysobactin receptor Flags: Precursor	1.83532312	780	5	1.28E-100	50.6%	2	C:membrane; F:binding			G3DSA:1.10.10.1550 (GENE3D); IPR008807 (PFAM)
			MMSR116_RS13925	TonB-dependent receptor	YNCD_ECOLI ame: Full=Probable -dependent receptor Flags: Precursor	-1.748519161	856	5	2.04E-9	48.2%	12	F:cobalamin transporter activity; P:ion transport; P:transmembrane transport; F:porin activity; F:metal ion binding; C:membrane; C:integral component of membrane; C:pore complex; C:cell outer membrane; P:cobalamin transport; F:receptor activity; P:transport			IPR001107 (PFAM)
			MMSR116_RS23315	TonB-dependent siderophore receptor	FCT_DICD3 ame: Full=Ferrichrysobactin receptor Flags: Precursor	-1.047502856	639	5	2.65E-52	43.6%	1	F:binding			IPR002295 (PRINTS); IPR003115 (SMART); IPR015840 (PIRSF); G3DSA:3.90.1530.30 (GENE3D); G3DSA:3.40.50.150 (GENE3D); IPR002941 (PFAM); IPR003115 (PFAM); PTHR13370 (PANTHER); PTHR13370:SF8 (PANTHER); IPR029063 (SUPERFAMILY); IPR036086 (SUPERFAMILY)
			MMSR116_RS21805	TonB-dependent siderophore receptor	FOXA_SALTS ame: Full=Ferrioxamine B receptor Flags: Precursor	-1.511467015	727	5	1.81E-21	39%	1	F:binding			IPR009656 (PFAM); IPR010915 (PIRSF); IPR010915 (TIGRFAM);

															IPR029058 (SUPERFAMILY)
			MMSR116_RS12465	porin family protein	OM31_BRUME ame: Full=31 kDa outer-membrane immunogenic Flags: Precursor	-1.534917073	279	5	1.23E-22	39.8%	8	P:ion transport; P:transmembrane transport; F:porin activity; C:membrane; C:integral component of membrane; C:pore complex; C:cell outer membrane; P:transport			G3DSA:3.40.50.10800 (GENE3D); G3DSA:3.40.50.10800 (GENE3D); G3DSA:3.40.50.10800 (GENE3D); IPR003473 (TIGRFAM); IPR003473 (PFAM); IPR003473 (PANTHER); IPR023066 (HAMAP); IPR036094 (SUPERFAMILY)
			MMSR116_RS12045	porin family protein	OM31_BRUME ame: Full=31 kDa outer-membrane immunogenic Flags: Precursor	-2.057390472	300	5	1.09E-16	43.4%	8	P:ion transport; P:transmembrane transport; F:porin activity; C:membrane; C:integral component of membrane; C:pore complex; C:cell outer membrane; P:transport			G3DSA:3.40.1620.10 (GENE3D); IPR006442 (PFAM); IPR036165 (SUPERFAMILY)
			MMSR116_RS03325	porin	OMP2B_BRUC2 ame: Full=Porin Omp2b Flags: Precursor	-2.354162175	566	5	1.26E-16	46.2%	8	P:ion transport; P:transmembrane transport; F:porin activity; C:membrane; C:integral component of membrane; C:pore complex; C:cell outer membrane; P:transport			IPR038301 (G3DSA:1.10.8. GENE3D); IPR010848 (PFAM)
			MMSR116_RS25895	porin family protein	OM31_BRUME ame: Full=31 kDa outer-membrane immunogenic Flags: Precursor	-1.55166049	289	5	4.82E-20	38%	8	P:ion transport; P:transmembrane transport; F:porin activity; C:membrane; C:integral component of membrane; C:pore complex; C:cell outer membrane; P:transport			no IPS match
			MMSR116_RS01625	transporter	---NA---	-1.447044258	323								no IPS match
			MMSR116_RS16305	AI-2E family transporter	Y630_RICPR ame: Full= transport RP630	1.029448384	374	5	1.3E-33	53.2%	1	C:plasma membrane			IPR022016 (PFAM); SSF158634 (SUPERFAMILY)
LPS and Capsule Biossintesis			MMSR116_RS13975	LPS biosynthesis protein	EXOP_RHIME ame: Full=Succinoglycan biosynthesis transport	-1.269008246	744	1	1.12E-16	46%	13	F:sugar efflux transmembrane transporter activity; P:proton transport; P:extracellular polysaccharide biosynthetic process; P:lipopolysaccharide biosynthetic process; C:extracellular region; P:transport; C:plasma membrane; F:nucleotide binding; F:ATP binding; P:polysaccharide biosynthetic process; P:extracellular carbohydrate transport; C:membrane; C:integral component of membrane			no IPS match
			MMSR116_RS12560	LPS biosynthesis protein	EXOP_RHIME ame: Full=Succinoglycan biosynthesis transport	1.117667585	491	1	4.79E-31	49%	13	F:sugar efflux transmembrane transporter activity; P:proton transport; P:extracellular polysaccharide biosynthetic process; P:lipopolysaccharide biosynthetic process; C:extracellular region; P:transport; C:plasma membrane; F:nucleotide binding; F:ATP binding; P:polysaccharide biosynthetic process; P:extracellular carbohydrate transport; C:membrane; C:integral component of membrane			IPR005139 (SMART); IPR004374 (TIGRFAM); G3DSA:3.30.160.20 (GENE3D); G3DSA:3.30.70.1660 (GENE3D); IPR005139 (PFAM); IPR000352 (PFAM); G3DSA:1.20.58.410 (GENE3D); PTHR43116:SF3 (PANTHER); PTHR43116 (PANTHER); IPR004374 (HAMAP); SSF75620 (SUPERFAMILY)
			MMSR116_RS03480	LPS biosynthesis protein	BEXC_HAEIF ame: Full=Capsule polysaccharide export inner-membrane	-1.016439631	435	5	6.29E-36	48.4%	2	P:transport; P:single-organism process			IPR002583 (TIGRFAM); IPR036510

															(G3DSA:1.20.58.GENE3D); IPR002583 (PFAM); PTHR33398:SF1 (PANTHER); IPR002583 (PANTHER); IPR002583 (PRODOM); IPR002583 (HAMAP); IPR036510 (SUPERFAMILY)
			MMSR116_RS24035	lipopolysaccharide biosynthesis protein	WZXC_ECOLI ame: Full=Lipopolysaccharide biosynthesis	-1.279064241	477	4	1.67E-29	44.5%	1	C:membrane			IPR006685 (PFAM); PTHR30566 (PANTHER); PTHR30566:SF5 (PANTHER); IPR010920 (SUPERFAMILY)
			MMSR116_RS20520	capsule polysaccharide biosynthesis protein	KPSS5_ECOLX ame: Full=Capsule polysaccharide export	-1.254505444	435	4	2.95E-60	48%	7	P:polysaccharide transport; P:polysaccharide biosynthetic process; C:membrane; P:carbohydrate transport; P:nitrogen fixation; P:transport; C:plasma membrane			IPR038740 (PFAM); IPR016181 (SUPERFAMILY)
			MMSR116_RS14895	group 1 glycosyl transferase	MSHA_JONDD ame: Full=D-inositol 3-phosphate glycosyltransferase ame: Full=N-acetylglucosamine-inositol-phosphate N-acetylglucosaminyltransferase Short= c-Ins-P N-acetylglucosaminyltransferase	-1.339501171	408	5	3.49E-11	42.8%	6	F:magnesium ion binding; F:transferase activity, transferring glycosyl groups; F:acetylglucosaminyltransferase activity; F:metal ion binding; F:transferase activity; P:mycothiol biosynthetic process			no IPS match
Biofilm and cell adhesion		Biofilm and cell adhesion	MMSR116_RS19060	hypothetical protein	CSGA_SALTY ame: Full=Major curlin subunit ame: Full=Fimbrin SEF17 Flags: Precursor	-1.247862243	695	3	1.22E-5	57%	4	P:single-species biofilm formation; P:cell adhesion; C:pilus; P:amyloid fibril formation			no IPS match
		BIOFILM	MMSR116_RS21670	hypothetical protein	ALGJ_PSEFL ame: Full=Probable alginate O-acetylase ame: Full=Alginate biosynthesis Flags: Precursor	1.264019029	320	4	7.39E-9	45.5%	7	F:transferase activity, transferring acyl groups; C:membrane; P:alginic acid biosynthetic process; C:periplasmic space; P:alginic acid acetylation; F:transferase activity; C:plasma membrane			G3DSA:1.20.1250.20 (GENE3D); IPR011701 (PFAM); PTHR43124:SF3 (PANTHER); PTHR43124 (PANTHER); IPR020846 (PROSITE_PROFILES); IPR036259 (SUPERFAMILY)
		cell adhesion	MMSR116_RS30445	Flp family type IVb pilin	cell adhesion	-1.125668136	54								IPR009915 (PFAM); G3DSA:1.20.120.1630 (GENE3D); IPR033580 (PANTHER)
		cell adhesion	MMSR116_RS12315	outer membrane protein assembly factor BamA	BAMA_NEIMB ame: Full=Outer membrane assembly factor ame: Full=Outer membrane Omp85 Flags: Precursor	-1.194987442	856	5	2.67E-73	43.2%	7	P:membrane assembly; P:Gram-negative-bacterium-type cell outer membrane assembly; C:membrane; C:integral component of membrane; C:cell outer membrane; C:outer membrane; P:protein insertion into membrane			IPR010275 (PFAM); IPR009045 (G3DSA:3.30.1380.GENE3D); IPR009045 (SUPERFAMILY)
		cell adhesion	MMSR116_RS22310	outer membrane protein assembly factor BamE	BAME_VIBCH ame: Full=Outer membrane assembly factor Flags: Precursor	-1.147066215	165	5	6.07E-5	52.6%	5	P:Gram-negative-bacterium-type cell outer membrane assembly; C:membrane; C:cell outer membrane; C:outer membrane; P:protein insertion into membrane			G3DSA:2.40.160.20 (GENE3D); PTHR34001 (PANTHER); IPR011250 (SUPERFAMILY)
Pigments		**Photosynthesis - light-independent protochlorophyllide reductase**	MMSR116_RS21975	ferredoxin:protochlorophyllide reductase	BCHL_METRJ ame: Full=Light-independent protochlorophyllide reductase iron-sulfur ATP-binding	1.830028914	297	5	0.00E+00	96.2%	8	F:ATP binding; F:oxidoreductase activity, acting on the CH-CH group of donors, iron-sulfur protein as acceptor; F:metal ion binding; P:photosynthesis, dark reaction; P:light-independent bacteriochlorophyll biosynthetic process; P:oxidation-reduction process; F:4 iron, 4 sulfur cluster			no IPS match

					Short=DPOR subunit L Short=LI-POR subunit L							binding; F:oxidoreductase activity, acting on iron-sulfur proteins as donors			
		Photosynthesis	MMSR116_RS09450	photosystem reaction center subunit H	---NA---	1.440146213	113								IPR038696 (G3DSA:2.60.40. GENE3D); IPR010642 (PFAM)
		bacteriochlorophyll	MMSR116_RS21940	bacteriochlorophyll/chlorophyll synthetase	BCHG_RHOS4 ame: Full=Bacteriochlorophyll synthase 33 kDa chain ame: Full=Geranylgeranyl bacteriochlorophyll synthase	1.317214714	294	5	1.08E-108	61.6%	4	F:prenyltransferase activity; C:membrane; C:chloroplast thylakoid; P:bacteriochlorophyll biosynthetic process		EC:1.1 .99.8; EC:1.1 .2.8	IPR018391 (SMART); G3DSA:2.140.10 .10 (GENE3D); IPR017512 (TIGRFAM); IPR002372 (PFAM); IPR002372 (PFAM); PTHR32303 (PANTHER); PTHR32303:SF1 6 (PANTHER); IPR011047 (SUPERFAMILY)
		carotenoid biosynthetic process	MMSR116_RS09475	cytochrome P450	LUT5_ARATH ame: Full= LUTEIN DEFICIENT chloroplastic ame: Full=Cytochrome P450 97A3 Flags: Precursor	1.110792955	462	5	1.92E-52	49.4%	6	P:carotenoid biosynthetic process; F:oxidoreductase activity, acting on paired donors, with incorporation or reduction of molecular oxygen; C:membrane; C:chloroplast envelope; F:binding; F:monooxygenase activity		EC:2.7 .7.65; EC:3.1 .4.52	IPR000160 (SMART); IPR003660 (SMART); IPR000014 (SMART); IPR001633 (SMART); G3DSA:3.30.70. 270 (GENE3D); IPR035919 (G3DSA:3.20.20. GENE3D); PF12860 (PFAM); IPR001633 (PFAM); IPR000014 (PFAM); IPR003660 (PFAM); IPR024478 (PFAM); G3DSA:1.20.148 0.50 (GENE3D); G3DSA:3.30.450 .20 (GENE3D); IPR000160 (TIGRFAM); IPR000160 (PFAM); PTHR44757 (PANTHER); PTHR44757:SF2 (PANTHER); IPR003660 (PROSITE_PROFI LES); IPR000160 (PROSITE_PROFI LES); IPR001633 (PROSITE_PROFI LES); IPR035965 (SUPERFAMILY); IPR029787 (SUPERFAMILY); SSF158472 (SUPERFAMILY); IPR035965 (SUPERFAMILY); IPR035919 (SUPERFAMILY)

		pigment precursor	MMSR116_RS21930	geranylgeranyl diphosphate reductase	BCHP_RHOCB ame: Full=Geranylgeranyl diphosphate reductase ame: Full=Geranylgeranyl reductase	1.325018272	408	5	1.04E-121	54%	8	P:chlorophyll biosynthetic process; F:oxidoreductase activity; C:membrane; C:chloroplast thylakoid; C:chloroplast envelope; F:binding; P:phospholipid metabolic process; P:terpenoid metabolic process		no IPS match
		chemotaxis	MMSR116_RS22055	HAMP domain-containing protein	MCP4_THEMA ame: Full=Methyl-accepting chemotaxis 4	-1.290457648	579	5	5.11E-54	50%	6	P:chemotaxis; P:signal transduction; C:membrane; C:integral component of membrane; F:signal transducer activity; C:plasma membrane		IPR010432 (PFAM)
		chemotaxis	MMSR116_RS26365	chemotaxis phosphatase CheZ	CHEZ_SHELP ame: Full= phosphatase ame: Full=Chemotaxis	-1.02637435	177	2	3.61E-5	38%	9	P:chemotaxis; F:phosphoprotein phosphatase activity; C:bacterial-type flagellum; P:archaeal or bacterial-type flagellum-dependent cell motility; C:cytoplasm; F:catalytic activity; P:protein dephosphorylation; P:regulation of chemotaxis; F:hydrolase activity		IPR000943 (PRINTS); G3DSA:1.20.140.160 (GENE3D); IPR009042 (PFAM); IPR007630 (PFAM); IPR012759 (TIGRFAM); IPR014284 (TIGRFAM); IPR007627 (PFAM); IPR000943 (PIRSF); G3DSA:1.20.120.1810 (GENE3D); PTHR30376:SF4 (PANTHER); PTHR30376 (PANTHER); IPR012759 (HAMAP); IPR013325 (SUPERFAMILY); IPR013324 (SUPERFAMILY)
		chemotaxis	MMSR116_RS26370	response regulator	CHEY_ENTCL ame: Full=Chemotaxis	-1.14629955	127	5	6.09E-43	71.2%	8	F:magnesium ion binding; P:chemotaxis; F:protein binding; F:acetyltransferase activity; P:internal peptidyl-lysine acetylation; P:phosphorelay signal transduction system; C:cytosol; P:bacterial-type flagellum-dependent cell motility		IPR004329 (PFAM); G3DSA:2.40.50.140 (GENE3D); IPR004329 (PANTHER); IPR004329 (HAMAP); IPR036127 (SUPERFAMILY)
		signal transduction	MMSR116_RS00370	histidine kinase	LOVHK_BRUA1 ame: Full=Blue-light-activated histidine kinase	1.13915372	239	5	7.3E-19	51.2%	1	P:cellular process		no IPS match
		Sinalling	MMSR116_RS25880	GGDEF domain-containing protein	VDCA_VIBCH ame: Full=Diguanylate cyclase Short=DGC	-1.171911306	352	5	1.5E-46	55.6%	6	F:diguanylate cyclase activity; F:protein binding; P:negative regulation of cellular component movement; P:single-species submerged biofilm formation; P:single-organism process; C:plasma membrane		IPR001128 (PRINTS); IPR002401 (PRINTS); IPR001128 (PFAM); IPR036396 (G3DSA:1.10.630.GENE3D); PTHR24291 (PANTHER); PTHR24291:SF109 (PANTHER); IPR036396 (SUPERFAMILY)
		signal transduction,	MMSR116_RS21395	calcium-binding protein	FRPC_NEIMB ame: Full=Iron-regulated	1.072778802	174	5	6.81E-22	64.6%	2	C:membrane; P:multi-organism process		no IPS match
		signal transduction AMPc	MMSR116_RS05500	cyclic nucleotide-binding protein	YCCS_ECOLI ame: Full=Inner membrane	1.323197794	721	5	1.57E-5	44.4%	3	C:membrane; C:integral component of membrane; C:plasma membrane		PTHR34001 (PANTHER); IPR011250 (SUPERFAMILY)
		regulatory role bind AMP or ATP	MMSR116_RS09735	CBS domain-containing protein	CBSX3_ARATH ame: Full=CBS domain-containing mitochondrial Flags: Precursor	0.983207739	143	5	2.35E-17	56%	2	F:metal ion binding; P:single-organism cellular process		IPR013108 (PFAM); IPR011059

															(G3DSA:2.30.40. GENE3D); G3DSA:3.20.20. 140 (GENE3D); PTHR32027:SF9 (PANTHER); PTHR32027 (PANTHER); IPR011059 (SUPERFAMILY); IPR032466 (SUPERFAMILY)
Response to stress			MMSR116_RS05230	HNH endonuclease	CAS9_CORDI ame: Full=CRISPR-associated endonuclease Cas9 ame: Full= as9	1.149912156	182	2	4.09E-5	52.5%	9	F:nucleic acid binding; F:DNA binding; P:nucleic acid phosphodiester bond hydrolysis; F:RNA binding; F:metal ion binding; F:endonuclease activity; F:nuclease activity; P:defense response to virus; F:hydrolase activity			IPR021232 (PFAM)
		protease /stress	MMSR116_RS19620	ATP-dependent Clp protease ATP-binding subunit	CLPA_RHOBL ame: Full= homolog	1.006752117	827	5	0.00E+00	69%	7	F:ATP binding; P:response to oxidative stress; F:protein binding; P:proteolysis; C:cytosol; F:ATP-dependent peptidase activity; P:protein unfolding			IPR003615 (SMART); G3DSA:3.30.40. 60 (GENE3D); IPR029471 (PFAM); PTHR33877 (PANTHER); PTHR33877:SF2 (PANTHER)
			MMSR116_RS03695	stress-induced protein	CON10_NEUCR ame: Full=Conidiation-specific 10	1.678977571	62	2	5.62E-8	75.5%	1	P:cell differentiation		EC:2.3 .1.41	IPR020841 (SMART); IPR014030 (PFAM); IPR016039 (G3DSA:3.40.47. GENE3D); IPR016039 (G3DSA:3.40.47. GENE3D); IPR014031 (PFAM); PTHR11712 (PANTHER); PTHR11712:SF3 06 (PANTHER); IPR016039 (SUPERFAMILY); IPR016039 (SUPERFAMILY)
		Glutathione	MMSR116_RS14755	glutathione S-transferase	YFCG_ECOLI ame: Full=Disulfide-bond oxidoreductase ame: Full=GSH-dependent disulfide-bond oxidoreductase ame: Full=GST N1-1 ame: Full=GST ame: Full=Organic hydroperoxidase	0.948027651	206	5	2.71E-62	54%	3	P:response to oxidative stress; F:disulfide oxidoreductase activity; F:transferase activity			IPR002549 (PFAM); IPR002549 (PANTHER); PTHR21716:SF3 5 (PANTHER)
		Glutathione	MMSR116_RS06710	glutathione-disulfide reductase	GSHR_BURCE ame: Full=Glutathione reductase Short=GR Short=GRase	1.098341894	460	5	1.37E-148	66.4%	8	C:chloroplast stroma; F:ATP binding; F:copper ion binding; P:glutathione metabolic process; C:mitochondrion; F:glutathione-disulfide reductase activity; P:response to chemical; P:single-organism cellular process			no IPS match
		Glutathione	MMSR116_RS27005	2-hydroxychromene-2-carboxylate isomerase	NSAD_SPHXE ame: Full=2-hydroxychromene-2-carboxylate isomerase Short=HCCA isomerase	1.276923307	202	4	2.3E-18	42.75%	2	F:catalytic activity; P:metabolic process		EC:4.3 .1.1	IPR000362 (PRINTS); PR00145 (PRINTS); G3DSA:1.20.200 .10 (GENE3D); G3DSA:1.10.40. 30 (GENE3D); IPR022761 (PFAM); IPR018951 (PFAM); IPR024083 (G3DSA:1.10.27 5.GENE3D); PTHR42696 (PANTHER);

															PTHR42696:SF2 (PANTHER); IPR008948 (SUPERFAMILY)
		Glutathione	MMSR116_RS13105	thiol:disulfide oxidoreductase	YFCG_ECOLI ame: Full=Disulfide-bond oxidoreductase ame: Full=GSH-dependent disulfide-bond oxidoreductase ame: Full=GST N1-1 ame: Full=GST ame: Full=Organic hydroperoxidase	0.909935697	235	5	3.58E-87	60.2%	5	P:response to oxidative stress; F:disulfide oxidoreductase activity; C:cytoplasm; P:glutathione metabolic process; F:glutathione transferase activity			IPR001898 (PFAM); IPR030676 (PANTHER)
		Thioredoxin	MMSR116_RS12040	thioredoxin	THIO1_CORNE ame: Full=Thioredoxin C-1	1.345521974	107	5	6.5E-50	79.4%	10	F:protein binding; F:protein disulfide oxidoreductase activity; C:cytoplasm; P:glycerol ether metabolic process; F:oxidoreductase activity, acting on a sulfur group of donors, disulfide as acceptor; P:cellular response to oxidative stress; P:cell redox homeostasis; P:oxidation-reduction process; P:protein folding; P:sulfate assimilation			IPR026355 (TIGRFAM); G3DSA:1.20.1250.20 (GENE3D); IPR011701 (PFAM); PTHR11360:SF3 (PANTHER); PTHR11360 (PANTHER); IPR020846 (PROSITE_PROFILES); IPR036259 (SUPERFAMILY)
		Thioredoxin	MMSR116_RS09440	peptide-methionine (S)-S-oxide reductase MsrA	MSRA_METS4 ame: Full=Peptide methionine sulfoxide reductase Short= -methionine-S-oxide reductase ame: Full=Peptide-methionine (S)-S-oxide reductase Short=Peptide Met(O) reductase	1.376076562	218	5	3.24E-118	78.8%	5	P:protein repair; P:response to oxidative stress; P:cellular protein modification process; F:peptide-methionine (S)-S-oxide reductase activity; P:oxidation-reduction process		EC:2.7.4.1	G3DSA:3.40.50.300 (GENE3D); IPR022488 (PFAM); IPR016898 (PIRSF); IPR022486 (TIGRFAM); PTHR34383 (PANTHER); PTHR34383:SF1 (PANTHER); IPR027417 (SUPERFAMILY)
		Thioredoxin	MMSR116_RS08100	peptide-methionine (S)-S-oxide reductase	MSRA_MYCMM ame: Full=Peptide methionine sulfoxide reductase Short= -methionine-S-oxide reductase ame: Full=Peptide-methionine (S)-S-oxide reductase Short=Peptide Met(O) reductase	1.42117269	172	5	2.49E-100	89.4%	5	P:protein repair; P:response to oxidative stress; P:cellular protein modification process; F:peptide-methionine (S)-S-oxide reductase activity; P:oxidation-reduction process			G3DSA:1.10.8.350 (GENE3D); G3DSA:1.10.530.10 (GENE3D); IPR031304 (PFAM); PTHR30163:SF8 (PANTHER); PTHR30163 (PANTHER); IPR023346 (SUPERFAMILY)
		Thioredoxin	MMSR116_RS00570	peptide-methionine (S)-S-oxide reductase	MSRA_STRAW ame: Full=Peptide methionine sulfoxide reductase Short= -methionine-S-oxide reductase ame: Full=Peptide-methionine (S)-S-oxide reductase Short=Peptide Met(O) reductase	1.299223403	172	5	8.31E-101	89.4%	5	P:protein repair; P:response to oxidative stress; P:cellular protein modification process; F:peptide-methionine (S)-S-oxide reductase activity; P:oxidation-reduction process			IPR000551 (PRINTS); IPR000551 (SMART); G3DSA:1.10.1660.10 (GENE3D); IPR015358 (PFAM); IPR000551 (PFAM); PTHR30204 (PANTHER); PTHR30204:SF33 (PANTHER); IPR000551 (PROSITE_PROFILES); IPR009061 (SUPERFAMILY)
		Thioredoxin	MMSR116_RS23230	peptide-methionine (R)-S-oxide reductase	MSRB1_RHIME ame: Full=Peptide methionine sulfoxide reductase 1 ame: Full=Peptide-methionine (R)-S-oxide reductase 1	1.718029588	136	5	6.2E-53	71.2%	3	F:metal ion binding; P:metabolic process; F:oxidoreductase activity, acting on a sulfur group of donors, disulfide as acceptor			IPR000709 (PRINTS); G3DSA:3.40.50.2300 (GENE3D); G3DSA:3.40.50.2300 (GENE3D); IPR028081

															(PFAM); PTHR30483 (PANTHER); IPR028082 (SUPERFAMILY)
		Anti oxidant system	MMSR116_RS16640	protein-methionine-sulfoxide reductase catalytic subunit MsrP	MSRP_PECCP ame: Full= -methionine-sulfoxide reductase catalytic subunit Flags: Precursor	1.276085261	316	5	2.53E-87	65.4%	2	F:oxidoreductase activity; P:metabolic process			no IPS match
			MMSR116_RS25875	iron-sulfur cluster assembly accessory protein	ISAM1_ARATH ame: Full=Iron-sulfur assembly -like mitochondrial Flags: Precursor	1.574298856	125	5	4.02E-36	70.4%	3	P:response to magnetism; C:mitochondrion; F:binding			G3DSA:3.40.190.10 (GENE3D); IPR015168 (PFAM); G3DSA:3.40.190.10 (GENE3D); PTHR30024 (PANTHER); PTHR30024:SF30 (PANTHER); SSF53850 (SUPERFAMILY)
			MMSR116_RS13660	iron-sulfur cluster assembly accessory protein	ERPA_RUTMC ame: Full=Iron-sulfur cluster insertion	1.544263394	108	5	3.13E-30	64.2%	1	F:binding			no IPS match
			MMSR116_RS09760	iron-sulfur cluster assembly scaffold protein	ISCU_THEMA ame: Full=Iron-sulfur cluster assembly scaffold ame: Full=Sulfur acceptor	1.234439956	147	5	1.91E-8	47%	1	F:binding			G3DSA:3.40.1090.10 (GENE3D); IPR002641 (PFAM); PTHR14226 (PANTHER); PTHR14226:SF29 (PANTHER); IPR002641 (PROSITE_PROFILES); IPR016035 (SUPERFAMILY)
		iron-sulfur (Fe-S) cluster assembly factor that facilitates [2Fe-2S] cluster	MMSR116_RS10375	glutaredoxin 3	GLRX_PSEAE ame: Full=Glutaredoxin	1.122817368	89	5	2.81E-31	68%	5	F:protein disulfide oxidoreductase activity; F:glutathione binding; C:cytosol; P:single-organism cellular process; P:single-organism metabolic process			IPR010292 (PIRSF); IPR010292 (PFAM); IPR010292 (PANTHER); PTHR37952:SF2 (PANTHER)
			MMSR116_RS19155	Fe-S cluster assembly protein SufB	Y074_SYNY3 ame: Full=UPF0051 slr0074	1.173724852	489	5	0.00E+00	79.4%	3	C:chloroplast; C:cyanelle; P:iron-sulfur cluster assembly			IPR000595 (SMART); IPR012318 (SMART); IPR000595 (PFAM); IPR036388 (G3DSA:1.10.10.GENE3D); IPR012318 (PFAM); IPR014710 (G3DSA:2.60.120.GENE3D); PTHR24567 (PANTHER); IPR012318 (PROSITE_PROFILES); IPR018490 (SUPERFAMILY); IPR036390 (SUPERFAMILY)
		iron detoxification and storage	MMSR116_RS22705	ferritin-like domain-containing protein	YCIE_ECOLI ame: Full=	1.511943	169	2	6.53E-20	50%	1	P:cellular response to DNA damage stimulus			no IPS match
		iron detoxification and storage	MMSR116_RS18390	ferritin-like domain-containing protein	---NA---	1.414686519	164								no IPS match
Host defence		host defence	MMSR116_RS07060	cupin domain-containing protein	---NA---	1.165978966	146								G3DSA:1.20.58.660 (GENE3D); IPR007420 (PFAM)

		Antitoxin	MMSR116_RS02995	type II toxin-antitoxin system Phd/YefM family	When bound to their toxin partners, they can bind DNA via the N terminus and repress the expression of operons containing genes encoding the toxin and the antitoxin	2.029313361	84								IPR004090 (PRINTS); IPR003660 (SMART); IPR004089 (SMART); IPR033480 (SMART); IPR033480 (PFAM); IPR004089 (PFAM); G3DSA:3.30.450.20 (GENE3D); G3DSA:1.10.287.950 (GENE3D); IPR003660 (PFAM); G3DSA:1.10.8.500 (GENE3D); PTHR32089 (PANTHER); PTHR32089:SF92 (PANTHER); IPR004089 (PROSITE_PROFILES); IPR003660 (PROSITE_PROFILES); IPR000727 (PROSITE_PROFILES); SSF58104 (SUPERFAMILY)
		Antitoxin	MMSR116_RS12905	antitoxin	VPB22_MYCTU ame: Full=Antitoxin 22	1.118844273	80	1	7.49E-2	55%	2	P:positive regulation of translation; P:growth			G3DSA:2.160.20.100 (GENE3D); IPR001646 (PFAM); G3DSA:2.160.20.80 (GENE3D); SSF141571 (SUPERFAMILY)
		virulence - stress	MMSR116_RS01175	invasion-associated locus B family protein	---NA---	-1.346599581	259							EC:1.1.1.22	IPR014027 (SMART); IPR014026 (PFAM); G3DSA:3.40.50.720 (GENE3D); IPR001732 (PFAM); G3DSA:3.40.50.720 (GENE3D); IPR028357 (PIRSF); IPR017476 (PIRSF); G3DSA:1.20.5.100 (GENE3D); IPR014027 (PFAM); IPR017476 (TIGRFAM); PTHR43750:SF3 (PANTHER); PTHR43750 (PANTHER); PS51257 (PROSITE_PROFILES); IPR008927 (SUPERFAMILY); IPR036220 (SUPERFAMILY); IPR036291 (SUPERFAMILY)
	Cleavage of Xf cell wall		MMSR116_RS27065	amidohydrolase	HIPO_CAMJE ame: Full=Hippurate hydrolase ame: Full=Benzoylglycine	1.200078563	385	5	5.6E-84	56.4%	5	C:intracellular membrane-bounded organelle; P:metabolic process; C:endomembrane system; C:cytoplasmic part; F:hydrolase activity			IPR012349 (G3DSA:2.30.110.GENE3D); IPR002563

					amidohydrolase ame: Full=Hippuricase										(PFAM); PTHR43567 (PANTHER); PTHR43567:SF1 (PANTHER); SSF50475 (SUPERFAMILY)
	Cleavage of Xf cell wall		MMSR116_RS27230	alpha/beta hydrolase	YN93_YEAST ame: Full=Uncharacterized hydrolase YNR064C	1.056267904	322	5	4.98E-70	46.4%	1	F:epoxide hydrolase activity			IPR018762 (PFAM); G3DSA:1.10.287.130 (GENE3D); IPR036890 (G3DSA:3.30.565.GENE3D)
	Cleavage of Xf cell wall		MMSR116_RS27470	alpha/beta hydrolase	YFHM_BACSU ame: Full=AB hydrolase superfamily	1.506332266	285	5	6.99E-80	55.6%	8	P:biological_process; F:catalytic activity; C:membrane; C:integral component of membrane; C:extracellular region; C:cellular_component; F:molecular_function; F:hydrolase activity		EC:1.17.4.1	IPR000788 (PRINTS); IPR013344 (TIGRFAM); IPR013678 (PFAM); G3DSA:3.20.70.20 (GENE3D); IPR000788 (PFAM); PTHR43371 (PANTHER); IPR029072 (SUPERFAMILY); SSF51998 (SUPERFAMILY)
	Cleavage of Xf cell wall		MMSR116_RS01255	cell wall hydrolase SleB	SLEB_OCEIH ame: Full=Spore cortex-lytic enzyme Short=SCLE Flags: Precursor	-1.325453939	201	3	1.67E-4	50.33%	6	P:spore germination; F:catalytic activity; P:cell wall organization; C:intracellular immature spore; P:sporulation resulting in formation of a cellular spore; F:hydrolase activity			IPR013153 (SMART); IPR013153 (PFAM); IPR010650 (PFAM); IPR016230 (PIRSF); PTHR30267 (PANTHER); IPR027417 (SUPERFAMILY)
	Cleavage of Xf cell wall		MMSR116_RS03180	glycoside hydrolase	LYSM1_STRGL ame: Full=Lysozyme M1 ame: Full=1,4-beta-N-acetylmuramidase M1 Flags: Precursor	-1.104893825	294	5	2.64E-32	50.2%	12	P:cell wall macromolecule catabolic process; P:carbohydrate metabolic process; P:cytolysis; C:extracellular space; F:catalytic activity; P:peptidoglycan catabolic process; P:metabolic process; C:extracellular region; P:defense response to bacterium; F:lysozyme activity; F:hydrolase activity; F:hydrolase activity, acting on glycosyl bonds			IPR029039 (G3DSA:3.40.50.GENE3D); IPR005025 (PFAM); PTHR30543:SF1 (PANTHER); PTHR30543 (PANTHER); IPR029039 (SUPERFAMILY)
	Cleavage of Xf cell wall		MMSR116_RS14565	hypothetical protein	MLTB_ECOLI ame: Full=Membrane-bound lytic murein transglycosylase B ame: Full=35 kDa soluble lytic transglycosylase ame: Full=Murein hydrolase B ame: Full=Slt35 Flags: Precursor	-1.049543614	264	1	3.44E-15	49%	2	P:peptidoglycan biosynthetic process; F:lytic transglycosylase activity			IPR012910 (PFAM); IPR000531 (PFAM); IPR037066 (G3DSA:2.170.130.GENE3D); PTHR32552:SF41 (PANTHER); PTHR32552 (PANTHER); SSF56935 (SUPERFAMILY)
			MMSR116_RS03845	lytic transglycosylase domain-containing protein	EXLYS_BPDPK ame: Full=Peptidoglycan hydrolase gp181 ame: Full=Gene product 181 Short=Gp181	-1.172796613	427	1	1.7E-22	52%	1	P:entry into host			PR00421 (PRINTS); IPR005746 (PIRSF); IPR005746 (TIGRFAM); G3DSA:3.40.30.10 (GENE3D); IPR013766 (PFAM);

															PTHR10438:SF357 (PANTHER); IPR005746 (PANTHER); IPR013766 (PROSITE_PROFILES); IPR036249 (SUPERFAMILY)
			MMSR116_RS24445	lytic transglycosylase domain-containing protein	YJBJ_BACSU ame: Full= murein lytic transglycosylase	1.981141161	229	5	1.63E-13	52.4%	2	P:entry into host via enzymatic degradation of host anatomical structure; F:catalytic activity			PTHR34001 (PANTHER); IPR011250 (SUPERFAMILY)
		sporulation	MMSR116_RS24220	SpoVR family protein	SP5R_BACSU ame: Full=Stage V sporulation R	0.947587315	525	2	0.00E+00	59%	1	P:cellular response to DNA damage stimulus			no IPS match
			MMSR116_RS01700	carbonic anhydrase	CYNT_ECO57 ame: Full=Carbonic anhydrase 1 ame: Full=Carbonate dehydratase 1	2.040381304	225	5	5.55E-65	63.4%	5	F:zinc ion binding; F:identical protein binding; C:cytoplasm; P:cyanate catabolic process; F:carbonate dehydratase activity			IPR019734 (SMART); IPR013105 (PFAM); IPR011990 (G3DSA:1.25.40.GENE3D); IPR013026 (PROSITE_PROFILES); IPR019734 (PROSITE_PROFILES); IPR011990 (SUPERFAMILY)
Nitrogen metabolism			MMSR116_RS16930	8-oxoguanine deaminase	OXODE_PSEAE ame: Full=8-oxoguanine deaminase	1.305669894	452	5	9.31E-123	53.6%	2	F:hydrolase activity, acting on carbon-nitrogen (but not peptide) bonds, in cyclic amidines; F:deaminase activity			IPR018383 (PFAM); IPR018383 (PANTHER); PTHR30106:SF2 (PANTHER)
		Cytochrome b556 - unknown	MMSR116_RS22400	formate dehydrogenase accessory protein FdhE	FDHE_RHIME ame: Full= homolog	-1.11346107	331	5	1.68E-48	56.8%	1	C:cytoplasm			IPR011102 (SMART); IPR036890 (G3DSA:3.30.565.GENE3D); IPR011102 (PFAM); PTHR41523 (PANTHER)
		molybdopterin biosynthesis	MMSR116_RS20965	molybdenum cofactor biosynthesis protein B	MOAB_ECO57 ame: Full=Molybdenum cofactor biosynthesis B	1.052593406	185	5	7.01E-46	59.4%	10	F:nucleotide binding; F:identical protein binding; P:heterocycle biosynthetic process; P:cofactor biosynthetic process; P:organic cyclic compound biosynthetic process; C:cytosol; P:organonitrogen compound biosynthetic process; F:transferase activity; P:molybdopterin cofactor metabolic process; P:organophosphate biosynthetic process			no IPS match
		rizobial NodB /cell wall	MMSR116_RS06855	allantoinase PuuE	CDA1_SCHPO ame: Full=Chitin deacetylase 1	1.193988686	387	3	3.78E-72	43.67%	9	F:hydrolase activity, acting on carbon-nitrogen (but not peptide) bonds; P:xylan catabolic process; P:carbohydrate metabolic process; P:polysaccharide catabolic process; F:catalytic activity; F:metal ion binding; P:cell wall organization; F:acetylxylan esterase activity; F:hydrolase activity			IPR007047 (PFAM)
		nitrogen compound metabolic process	MMSR116_RS10370	carbon-nitrogen hydrolase family protein	NIT1_YEREN ame: Full=Deaminated glutathione amidase Short=dGSH amidase ame: Full=Nitrilase homolog 1 Short=yeNit1	1.012457858	285	5	1.97E-53	58.6%	1	C:mitochondrion			no IPS match
			MMSR116_RS27340	NifU family protein	NFU1_DROSE ame: Full=NFU1 iron-sulfur cluster scaffold mitochondrial Flags: Precursor	1.230124575	188	5	6.97E-59	67.8%	7	P:biological_process; F:iron ion binding; C:mitochondrion; F:iron-sulfur cluster binding; C:cellular_component; F:molecular_function; P:iron-sulfur cluster assembly		EC:1.8.1.7	PR00411 (PRINTS); PR00368 (PRINTS); IPR036188 (G3DSA:3.50.50.GENE3D); IPR001100 (PIRSF); IPR004099 (PFAM); IPR016156 (G3DSA:3.30.390.GENE3D); IPR006324 (TIGRFAM);

															IPR036188 (G3DSA:3.50.50. GENE3D); IPR023753 (PFAM); PTHR43256 (PANTHER); PTHR43256:SF1 (PANTHER); IPR016156 (SUPERFAMILY); IPR036188 (SUPERFAMILY)
Unknow fuction			MMSR116_RS27330	DUF2934 domain-containing protein	ATPF2_RHOFT ame: Full=ATP synthase subunit b 2 ame: Full=ATP synthase F(0) sector subunit b 2 ame: Full=ATPase subunit I 2 ame: Full=F-type ATPase subunit b 2 Short=F-ATPase subunit b 2	-1.387436964	132	1	7.49E-2	75%	12	P:ion transport; P:proton transport; F:hydrogen ion transmembrane transporter activity; C:proton-transporting ATP synthase complex, coupling factor F(o); C:membrane; C:integral component of membrane; P:ATP biosynthetic process; P:transport; C:plasma membrane; P:ATP synthesis coupled proton transport; P:plasma membrane ATP synthesis coupled proton transport; F:proton-transporting ATP synthase activity, rotational mechanism			IPR032807 (PFAM); IPR003856 (PFAM); PTHR32309 (PANTHER); PTHR32309:SF13 (PANTHER)
			MMSR116_RS14885	DUF1150 domain-containing protein	---NA---	1.126227657	94								IPR000531 (PFAM); IPR036942 (G3DSA:2.40.170.GENE3D); IPR012910 (PFAM); IPR010105 (TIGRFAM); IPR037066 (G3DSA:2.170.130.GENE3D); PTHR32552 (PANTHER); PTHR32552:SF70 (PANTHER); SSF56935 (SUPERFAMILY)
			MMSR116_RS12650	DUF1465 domain-containing protein	---NA---	1.645032759	176								G3DSA:2.170.150.20 (GENE3D); IPR002579 (TIGRFAM); IPR002579 (PFAM); PTHR43234 (PANTHER); IPR002579 (PROSITE_PROFILES); IPR011057 (SUPERFAMILY)
			MMSR116_RS12470	DUF2076 domain-containing protein	Y170_BUCBPRecName: Full=Uncharacterized protein bbp_170; AltName: Full=yba2	1.633019249	255	3	3.61E-8	60.67%					IPR000674 (SMART); G3DSA:3.30.365.10 (GENE3D); IPR012368 (PIRSF); G3DSA:3.30.365.10 (GENE3D); G3DSA:3.30.365.10 (GENE3D); G3DSA:3.30.365.10 (GENE3D); G3DSA:3.90.1170.50 (GENE3D); IPR008274 (PFAM); PTHR11908:SF2 (PANTHER); PTHR11908 (PANTHER); IPR006311 (PROSITE_PROFILES); IPR037165 (SUPERFAMILY);

															IPR037165 (SUPERFAMILY)
			MMSR116_RS07015	DUF1203 domain-containing protein	---NA---	2.395699669	159								IPR036444 (G3DSA:1.20.90. GENE3D); PS51257 (PROSITE_PROFILES); IPR036444 (SUPERFAMILY)
			MMSR116_RS07710	DUF2852 domain-containing protein	---NA---	1.602143222	154								IPR019734 (SMART); IPR011990 (G3DSA:1.25.40. GENE3D); PF13414 (PFAM); IPR011990 (G3DSA:1.25.40. GENE3D); IPR011990 (G3DSA:1.25.40. GENE3D); IPR001440 (PFAM); PTHR44858 (PANTHER); IPR013026 (PROSITE_PROFILES); IPR019734 (PROSITE_PROFILES); IPR011990 (SUPERFAMILY)
			MMSR116_RS28610	DUF2735 domain-containing protein	GSTI_RHILE ame: Full=Glutamine synthetase translation inhibitor	2.147338058	59	1	1.75E-3	45%					no IPS match
			MMSR116_RS08695	DUF2157 domain-containing protein	---NA---	1.359178063	445								no IPS match
			MMSR116_RS27575	DUF1285 domain-containing protein	---NA---	1.208819124	194								IPR000537 (PFAM); G3DSA:1.20.120 .1780 (GENE3D); IPR006372 (TIGRFAM); G3DSA:1.10.357 .140 (GENE3D); PTHR42723 (PANTHER)
			MMSR116_RS09950	DUF179 domain-containing protein	Y6268_METS4 ame: Full=UPF0301 M446_6268	1.09269248	195	5	3.43E-108	80%					IPR001623 (SMART); IPR036869 (G3DSA:1.10.28 7.GENE3D); IPR001623 (PFAM); IPR007791 (PFAM); IPR029024 (G3DSA:1.10.36 80.GENE3D); IPR001623 (PROSITE_PROFILES); IPR029024 (SUPERFAMILY); IPR036869 (SUPERFAMILY)
			MMSR116_RS20455	DUF3597 domain-containing protein	---NA---	1.116917454	137								IPR000825 (PFAM); IPR010231 (TIGRFAM); PTHR30508 (PANTHER); PTHR30508:SF1 (PANTHER);

															IPR037284 (SUPERFAMILY)
			MMSR116_RS26380	DUF2093 domain-containing protein	---NA---	1.921268462	72								IPR003918 (PRINTS); IPR001750 (PFAM); PTHR42703 (PANTHER)
			MMSR116_RS26135	DUF937 domain-containing protein	YIDB_ECOLIRecName: Full=Uncharacterized protein YidB	1.103027937	235	1	9.4E-9	60%					no IPS match
			MMSR116_RS25990	DUF2794 domain-containing protein	---NA---	1.082722343	124								IPR001623 (PRINTS); IPR001623 (SMART); IPR036869 (G3DSA:1.10.28 7.GENE3D); IPR001623 (PFAM); IPR001623 (PROSITE_PROFI LES); IPR036869 (SUPERFAMILY)
			MMSR116_RS20055	DUF4926 domain-containing protein	---NA---	1.47341565	83								IPR036444 (G3DSA:1.20.90. GENE3D); IPR036444 (SUPERFAMILY)
			MMSR116_RS22250	DUF465 domain-containing protein	---NA---	1.881405117	60								no IPS match
			MMSR116_RS25415	DUF1508 domain-containing protein	Y1088_NEIMB ame: Full=UPF0339 NMB1088	1.091554947	61	5	4.34E-15	69%					IPR000182 (PFAM); G3DSA:3.40.630 .30 (GENE3D); PTHR43441:SF1 (PANTHER); PTHR43441 (PANTHER); IPR000182 (PROSITE_PROFI LES); IPR016181 (SUPERFAMILY)
			MMSR116_RS13505	DUF882 domain-containing protein	YCBK_ECO57RecName: Full=Uncharacterized protein YcbK; Flags: Precursor	-1.44148514	481	3	4.63E-28	58.33%	2	C:membrane; C:integral component of membrane		EC:2.7 .7.65	IPR000160 (SMART); IPR000160 (TIGRFAM); G3DSA:3.30.70. 270 (GENE3D); IPR000160 (PFAM); PTHR45138 (PANTHER); IPR000160 (PROSITE_PROFI LES); IPR029787 (SUPERFAMILY)
			MMSR116_RS14240	pentapeptide repeat-containing protein	PIPB2_SALTY ame: Full=Secreted effector 2 ame: Full=Type III effector 2	1.419673342	266	5	2.06E-19	50.8%	4	C:membrane; C:extracellular region; P:pathogenesis; C:host cell membrane			G3DSA:3.75.10. 10 (GENE3D); IPR033195 (PANTHER); SSF55909 (SUPERFAMILY)
			MMSR116_RS09630	band 7 protein	---NA---	2.353796332	325								no IPS match
Hypothetical protein			MMSR116_RS03055	hypothetical protein	FLGE_BRUAB ame: Full=Flagellar hook	-1.352325453	424	5	1.1E-30	48%	1	C:cell part		EC:1.8 .4.11	IPR002569 (PFAM); IPR036509 (G3DSA:3.30.10 60.GENE3D); IPR002569 (TIGRFAM); PTHR42799

															(PANTHER); PTHR42799:SF2 (PANTHER); IPR002569 (HAMAP); IPR036509 (SUPERFAMILY)
			MMSR116_RS24385	hypothetical protein	---NA---	1.248287421	118								IPR003494 (SMART); IPR020823 (TIGRFAM); G3DSA:3.30.1490.110 (GENE3D); IPR020823 (PIRSF); G3DSA:3.30.420.40 (GENE3D); IPR003494 (PFAM); G3DSA:3.90.640.10 (GENE3D); G3DSA:3.30.420.40 (GENE3D); PF14450 (PFAM); PTHR32432 (PANTHER); PTHR32432:SF4 (PANTHER); IPR020823 (HAMAP); SSF53067 (SUPERFAMILY); SSF53067 (SUPERFAMILY)
			MMSR116_RS24380	hypothetical protein	---NA---	-1.329257911	376								IPR001270 (PRINTS); IPR003593 (SMART); IPR019489 (SMART); IPR019489 (PFAM); IPR013461 (TIGRFAM); G3DSA:1.10.8.60 (GENE3D); IPR004176 (PFAM); G3DSA:3.40.50.300 (GENE3D); IPR003959 (PFAM); IPR036628 (G3DSA:1.10.1780.GENE3D); G3DSA:3.40.50.300 (GENE3D); G3DSA:1.10.8.60 (GENE3D); IPR003959 (PFAM); PTHR11638:SF111 (PANTHER); PTHR11638 (PANTHER); IPR027417 (SUPERFAMILY); IPR036628 (SUPERFAMILY); IPR027417 (SUPERFAMILY)
			MMSR116_RS24360	hypothetical protein	---NA---	3.055545399	96							EC:3.3.1.1	IPR015878 (SMART); IPR000043

															(SMART); G3DSA:3.40.50.1480 (GENE3D); G3DSA:3.40.50.720 (GENE3D); IPR015878 (PFAM); IPR000043 (PFAM); IPR000043 (PIRSF); IPR000043 (TIGRFAM); PTHR23420:SF0 (PANTHER); IPR000043 (PANTHER); IPR034373 (HAMAP); SSF52283 (SUPERFAMILY); IPR036291 (SUPERFAMILY)
			MMSR116_RS24215	hypothetical protein	Y6755_BRADU ame: Full=UPF0229 bll6755	0.885553514	433	5	4.96E-167	66.8%					IPR000454 (PRINTS); IPR002379 (PFAM); IPR005953 (TIGRFAM); IPR038662 (G3DSA:1.20.20.GENE3D); IPR000454 (PANTHER); IPR000454 (HAMAP); IPR035921 (SUPERFAMILY)
			MMSR116_RS24205	hypothetical protein	---NA---	0.99370564	147								IPR002327 (PRINTS); IPR009056 (PFAM); IPR036909 (G3DSA:1.10.760.GENE3D); PTHR11961:SF22 (PANTHER); IPR002327 (PANTHER); IPR009056 (PROSITE_PROFILES); IPR036909 (SUPERFAMILY)
			MMSR116_RS24150	hypothetical protein	---NA---	1.879551619	84								no IPS match
			MMSR116_RS15245	hypothetical protein	---NA---	1.152258942	66								no IPS match
			MMSR116_RS13960	hypothetical protein	---NA---	-1.303916069	117								IPR001845 (PRINTS); IPR001845 (SMART); IPR036388 (G3DSA:1.10.10.GENE3D); IPR013216 (PFAM); G3DSA:3.40.50.150 (GENE3D); IPR001845 (PFAM); PTHR43464:SF17 (PANTHER); PTHR43464 (PANTHER);

															IPR001845 (PROSITE_PROFILES); IPR036390 (SUPERFAMILY); IPR029063 (SUPERFAMILY)
			MMSR116_RS12625	hypothetical protein	---NA---	1.233121186	129								IPR001041 (PFAM); G3DSA:1.10.150.120 (GENE3D); IPR012675 (G3DSA:3.10.20.GENE3D); IPR002888 (PFAM); PTHR44379 (PANTHER); IPR001041 (PROSITE_PROFILES); IPR036010 (SUPERFAMILY); IPR036884 (SUPERFAMILY)
			MMSR116_RS12455	hypothetical protein	---NA---	1.246085478	63								IPR010287 (PFAM); IPR012347 (G3DSA:1.20.1260.GENE3D); PTHR30565 (PANTHER); PTHR30565:SF1 (PANTHER); IPR009078 (SUPERFAMILY)
			MMSR116_RS11690	hypothetical protein	---NA---	1.4822949	224								IPR019949 (TIGRFAM); IPR036661 (G3DSA:3.20.20.GENE3D); IPR011251 (PFAM); PTHR30137:SF6 (PANTHER); PTHR30137 (PANTHER); IPR036661 (SUPERFAMILY)
			MMSR116_RS05605	hypothetical protein	---NA---	1.452271212	148								G3DSA:3.40.190.10 (GENE3D); G3DSA:3.40.190.10 (GENE3D); PF13531 (PFAM); IPR005669 (TIGRFAM); IPR005669 (PANTHER); SSF53850 (SUPERFAMILY)
			MMSR116_RS05655	hypothetical protein	---NA---	1.058976227	157							EC:1.97.1	IPR013785 (G3DSA:3.20.20.GENE3D); IPR001155 (PFAM); PTHR22893 (PANTHER); PTHR22893:SF14 (PANTHER); SSF51395 (SUPERFAMILY)
			MMSR116_RS31300	hypothetical protein	---NA---	1.883643499	91								IPR037873 (G3DSA:3.30.1450.GENE3D); IPR007450 (PFAM); IPR026592

															(PANTHER); PS51257 (PROSITE_PROFILES)
			MMSR116_RS05660	hypothetical protein	---NA---	1.286525316	71								G3DSA:1.20.1250.20 (GENE3D); IPR011701 (PFAM); PTHR23508 (PANTHER); PTHR23508:SF20 (PANTHER); IPR020846 (PROSITE_PROFILES); IPR036259 (SUPERFAMILY)
			MMSR116_RS05705	hypothetical protein	---NA---	1.562666018	105								no IPS match
			MMSR116_RS03700	hypothetical protein	---NA---	1.234141878	121								IPR009394 (PFAM); IPR011856 (G3DSA:3.40.1350.GENE3D); IPR009394 (PIRSF)
			MMSR116_RS27000	hypothetical protein	---NA---	2.647016966	69								PR00091 (PRINTS); IPR005971 (TIGRFAM); IPR000392 (PFAM); IPR000392 (PIRSF); G3DSA:3.40.50.300 (GENE3D); IPR000392 (PANTHER); IPR005971 (PTHR42864:PANTHER); IPR005971 (HAMAP); IPR000392 (PROSITE_PROFILES); IPR027417 (SUPERFAMILY)
			MMSR116_RS27595	hypothetical protein	---NA---	1.186874323	209								PR00420 (PRINTS); IPR011777 (TIGRFAM); IPR023753 (PFAM); IPR010253 (TIGRFAM); IPR002938 (PFAM); IPR036188 (G3DSA:3.50.50.GENE3D); PTHR42685 (PANTHER); IPR036188 (SUPERFAMILY)
			MMSR116_RS28235	hypothetical protein	---NA---	1.439558942	87								no IPS match
			MMSR116_RS28525	hypothetical protein	---NA---	1.162851911	112								G3DSA:3.30.9.10 (GENE3D); IPR036188 (G3DSA:3.50.50.GENE3D); IPR006076 (PFAM); PTHR13847 (PANTHER);

															PTHR13847:SF250 (PANTHER); IPR036188 (SUPERFAMILY); SSF54373 (SUPERFAMILY)
			MMSR116_RS28545	hypothetical protein	---NA---	2.196266531	79								no IPS match
			MMSR116_RS29570	hypothetical protein	---NA---	1.633520361	60								IPR009009 (PFAM); IPR012997 (TIGRFAM); IPR036908 (G3DSA:2.40.40. GENE3D); PTHR34183 (PANTHER); PTHR34183:SF1 (PANTHER); IPR034718 (HAMAP); IPR036908 (SUPERFAMILY)
			MMSR116_RS08700	hypothetical protein	---NA---	1.494512997	223								no IPS match
			MMSR116_RS08860	hypothetical protein	CHPT_BRUA2 ame: Full= phosphotransferase ame: Full=Histidine phosphotransferase	-1.269531231	241	1	1.54E-29	62%					IPR006680 (PFAM); G3DSA:3.20.20.140 (GENE3D); IPR011059 (G3DSA:2.30.40. GENE3D); PTHR11271:SF37 (PANTHER); PTHR11271 (PANTHER); IPR032466 (SUPERFAMILY); IPR011059 (SUPERFAMILY)
			MMSR116_RS08915	hypothetical protein	---NA---	2.019291405	87								IPR011105 (PFAM); G3DSA:1.10.10.2520 (GENE3D); PS51257 (PROSITE_PROFILES)
			MMSR116_RS09420	hypothetical protein	---NA---	2.256621725	89								no IPS match
			MMSR116_RS09830	hypothetical protein	---NA---	-1.19589816	74								IPR001650 (SMART); IPR014001 (SMART); IPR011545 (PFAM); G3DSA:3.40.50.300 (GENE3D); G3DSA:3.40.50.300 (GENE3D); IPR001650 (PFAM); PTHR24031:SF349 (PANTHER); PTHR24031 (PANTHER); IPR001650 (PROSITE_PROFILES); IPR014014 (PROSITE_PROFILES); IPR014001 (PROSITE_PROFILES); IPR027417 (SUPERFAMILY)

			MMSR116_RS05495	hypothetical protein	---NA---	2.272628587	75								IPR038162 (G3DSA:2.60.40.GENE3D); IPR013783 (G3DSA:2.60.40.GENE3D); IPR014880 (PFAM); IPR030831 (TIGRFAM); IPR032711 (PFAM); IPR014756 (SUPERFAMILY)
			MMSR116_RS05550	hypothetical protein	---NA---	1.252647944	77								G3DSA:3.40.50.10610 (GENE3D); IPR005586 (PFAM); PTHR36698 (PANTHER); PTHR36698:SF3 (PANTHER); PS51257 (PROSITE_PROFILES); SSF159594 (SUPERFAMILY)
			MMSR116_RS26110	hypothetical protein	---NA---	1.250887845	82								IPR026569 (PFAM); IPR037147 (G3DSA:2.30.170.GENE3D); IPR001383 (TIGRFAM); IPR026569 (PANTHER); IPR001383 (HAMAP); IPR034704 (SUPERFAMILY)
			MMSR116_RS24515	hypothetical protein	---NA---	1.926636403	92								IPR001453 (SMART); IPR001453 (TIGRFAM); IPR012245 (PIRSF); IPR001453 (PFAM); IPR036425 (G3DSA:3.40.980.GENE3D); IPR013484 (TIGRFAM); IPR012245 (PANTHER); PTHR43232:SF3 (PANTHER); IPR036425 (SUPERFAMILY)
			MMSR116_RS24580	hypothetical protein	---NA---	1.166874756	152								G3DSA:1.20.1250.20 (GENE3D); IPR011701 (PFAM); PTHR23501 (PANTHER); PTHR23501:SF98 (PANTHER); IPR020846 (PROSITE_PROFILES); IPR036259 (SUPERFAMILY)
			MMSR116_RS24620	hypothetical protein	---NA---	1.610033884	122								IPR005511 (PRINTS); IPR011042 (G3DSA:2.120.10.GENE3D);

															IPR013658 (PFAM); PTHR10907:SF76 (PANTHER); PTHR10907 (PANTHER); SSF63829 (SUPERFAMILY)
			MMSR116_RS00460	hypothetical protein	---NA---	1.090119398	183								IPR005814 (PFAM); IPR015422 (G3DSA:3.90.1150.GENE3D); IPR015421 (G3DSA:3.40.640.GENE3D); IPR005814 (PIRSF); PTHR42684 (PANTHER); PTHR42684:SF4 (PANTHER); IPR015424 (SUPERFAMILY)
			MMSR116_RS23765	hypothetical protein	---NA---	1.783529633	85								IPR021866 (PFAM); IPR038396 (G3DSA:3.40.50.GENE3D); IPR036513 (SUPERFAMILY)
			MMSR116_RS23515	hypothetical protein	---NA---	1.922385956	69								no IPS match
			MMSR116_RS22285	hypothetical protein	---NA---	1.463654622	164								PR00313 (PRINTS); IPR011049 (G3DSA:2.150.10.GENE3D); IPR011049 (G3DSA:2.150.10.GENE3D); IPR001343 (PFAM); IPR011049 (SUPERFAMILY)
			MMSR116_RS22270	hypothetical protein	---NA---	1.0386519	156								IPR018044 (PRINTS); G3DSA:3.40.710.10 (GENE3D); IPR001967 (PFAM); PTHR21581 (PANTHER); PTHR21581:SF20 (PANTHER); IPR012338 (SUPERFAMILY)
			MMSR116_RS21810	hypothetical protein	---NA---	-1.755563283	488								IPR011547 (PFAM); IPR001902 (PANTHER); PTHR11814:SF184 (PANTHER)
			MMSR116_RS01880	hypothetical protein	---NA---	1.084167517	107								IPR025737 (PFAM)
			MMSR116_RS17280	hypothetical protein	---NA---	-0.986908344	66							EC:1.1.1.244	G3DSA:3.40.50.1970 (GENE3D); IPR001670 (PFAM); G3DSA:1.20.1090.10 (GENE3D); PTHR11496:SF93 (PANTHER); PTHR11496

															(PANTHER); SSF56796 (SUPERFAMILY)
			MMSR116_RS16960	hypothetical protein	---NA---	1.63936624	81							EC:1.2.1.27; EC:1.2.1.3; EC:1.2.1.18	IPR016163 (G3DSA:3.40.309.GENE3D); IPR010061 (TIGRFAM); IPR016162 (G3DSA:3.40.605.GENE3D); IPR015590 (PFAM); PTHR43866:SF4 (PANTHER); IPR010061 (PANTHER); IPR016161 (SUPERFAMILY)
			MMSR116_RS01210	hypothetical protein	---NA---	1.55011465	107							EC:2.3.1.30	IPR010493 (SMART); G3DSA:2.160.10.10 (GENE3D); IPR001451 (PFAM); IPR010493 (PFAM); IPR005881 (TIGRFAM); G3DSA:1.10.3130.10 (GENE3D); PTHR42811 (PANTHER); PTHR42811:SF4 (PANTHER); IPR011004 (SUPERFAMILY)
			MMSR116_RS01035	hypothetical protein	---NA---	1.268237081	85								IPR032568 (PFAM)
			MMSR116_RS30720	hypothetical protein	---NA---	1.023119833	177								PR00173 (PRINTS); IPR001991 (PFAM); IPR036458 (G3DSA:1.10.3860.GENE3D); PTHR42865 (PANTHER); IPR033380 (PTHR42865:PANTHER); PS51257 (PROSITE_PROFILES); IPR036458 (SUPERFAMILY)
			MMSR116_RS04135	hypothetical protein	---NA---	2.258050102	83								IPR002347 (PRINTS); SM00822 (SMART); IPR002347 (PFAM); G3DSA:3.40.50.720 (GENE3D); PTHR43391:SF27 (PANTHER); PTHR43391 (PANTHER); IPR036291 (SUPERFAMILY)
			MMSR116_RS21675	hypothetical protein	---NA---	1.913339653	74								IPR010287 (PFAM); IPR012347 (G3DSA:1.20.1260.GENE3D); PTHR30565:SF9

															(PANTHER); PTHR30565 (PANTHER); IPR009078 (SUPERFAMILY)
			MMSR116_RS01705	hypothetical protein	---NA---	1.846789278	112								G3DSA:1.10.10.1550 (GENE3D); IPR008807 (PFAM)
			MMSR116_RS20310	hypothetical protein	---NA---	1.168804326	83								no IPS match
			MMSR116_RS16365	hypothetical protein	---NA---	1.81994079	191								IPR019626 (PFAM)
			MMSR116_RS17490	hypothetical protein	---NA---	1.495317871	69								no IPS match
			MMSR116_RS02920	hypothetical protein	CREA_ECO57 ame: Full= ame: Full=Catabolite regulation A Flags: Precursor	1.076792398	174	1	3.21E-44	73%	1	C:cytosol			IPR036812 (G3DSA:3.20.20.GENE3D); IPR023210 (PFAM); PTHR43364:SF5 (PANTHER); PTHR43364 (PANTHER); IPR036812 (SUPERFAMILY)
Total : 273			MMSR116_RS25090	hypothetical protein	Y6063_BRADURecName: Full=Uncharacterized protein bll6063	1.02680165	193	1	6.4E-12	52%	2	C:membrane; C:integral component of membrane			IPR035903 (G3DSA:2.60.300.GENE3D); IPR016092 (TIGRFAM); IPR000361 (PFAM); PTHR10072 (PANTHER); PTHR10072:SF41 (PANTHER); IPR035903 (SUPERFAMILY)

Table S6. Trends in genes detected in transcriptome and secretome of *Mm*.

locustag	Mm_co_PPT	Mm_PPT	Mm_co_TPM	Mm_TPM	Proteome ratio	Transcriptome ratio
MMSR116_RS00865	0.77	0.57	728.51	963.17	1.34	0.76
MMSR116_RS07030	15.95	7.50	1089.62	798.16	**2.13**	**1.37**
MMSR116_RS09540	4.37	4.28	498.57	365.07	**1.02**	**1.37**
MMSR116_RS09940	3.24	3.42	2579.86	2656.27	**0.95**	**0.97**
MMSR116_RS11970	29.55	7.01	1366.25	924.46	**4.22**	**1.48**
MMSR116_RS12040	36.86	44.59	2830.85	5132.78	**0.83**	**0.55**
MMSR116_RS12140	52.76	40.74	438.22	287.88	**1.30**	**1.52**
MMSR116_RS14980	2.14	0.86	1204.83	770.74	**2.50**	**1.56**
MMSR116_RS16505	61.05	63.26	3970.14	1868.01	0.97	2.13
MMSR116_RS16515	39.97	80.68	851.35	325.67	0.50	2.61
MMSR116_RS20185	18.62	9.75	1020.18	511.39	**1.91**	**1.99**
MMSR116_RS20190	500.33	4.73	750.15	364.23	**105.85**	**2.06**
MMSR116_RS20370	1.72	1.32	1195.88	1176.25	**1.30**	**1.02**
MMSR116_RS20480	0.68	0.40	986.09	556.53	**1.70**	**1.77**
MMSR116_RS23965	177.41	155.80	4759.12	1408.80	**1.14**	**3.38**
MMSR116_RS25265	0.91	8.87	7347.90	6676.29	0.10	1.10
MMSR116_RS27490	7.80	8.40	1630.13	651.58	0.93	2.50
MMSR116_RS29630	1.04	0.78	685.73	440.28	**1.34**	**1.56**

Table S7. Trends in genes detected in transcriptome and secretome of *Xf*.

Name	Xfp_co_PPT	Xfp_PPT	Xfp_co_TPM	Xfp_TPM	Proteome ratio	Transcriptome ratio
XF_0082	51.32	412.41	532.21	211.85	0.12	2.51
XF_0239	10.53	85.24	2093.05	820.72	0.12	2.55
XF_0343	9348.55	8742.67	9529.04	3071.42	**1.07**	**3.10**
XF_0357	12.81	418.63	122.54	37.11	0.03	3.30
XF_0363	1417.21	8918.05	2884.86	1283.20	0.16	2.25
XF_0550	6.44	48.98	243.94	98.69	0.13	2.47

XF_0565	71.36	984.90	418.32	171.10	0.07	2.44
XF_0615	61.55	363.02	4395.44	949.56	0.17	4.63
XF_0644	68.80	1381.47	1074.85	343.43	0.05	3.13
XF_0668	1677.88	1028.33	185.32	90.44	**1.63**	**2.05**
XF_0820	1.79	58.67	207.96	100.37	0.03	2.07
XF_0898	506.33	7945.16	244.59	80.36	0.06	3.04
XF_0964	322.86	3283.51	968.03	373.94	0.10	2.59
XF_0975	305.53	3196.04	288.67	93.58	0.10	3.08
XF_1011	626.44	424.26	57.71	25.68	**1.48**	**2.25**
XF_1026	3049.67	1983.71	411.67	185.12	**1.54**	**2.22**
XF_1219	31781.85	11249.77	4035.89	1526.69	**2.83**	**2.64**
XF_1547	28782.96	24207.11	1588.22	712.88	**1.19**	**2.23**
XF_1649	35.39	298.43	13.21	11.48	0.12	1.15
XF_1803	18342.24	18835.12	24109.20	6849.07	0.97	3.52
XF_1840	57.67	654.97	1408.53	389.58	0.09	3.62
XF_1851	46.44	453.28	582.00	286.41	0.10	2.03
XF_1887	23.62	248.56	425.54	204.34	0.10	2.08
XF_1897	20.51	388.34	266.98	100.61	0.05	2.65
XF_1981	1140.33	1261.49	644.08	298.78	0.90	2.16
XF_2237	846.58	832.74	328.55	116.71	**1.02**	**2.82**
XF_2340	15.16	139.73	943.47	251.21	0.11	3.76
XF_2349	1305.60	1257.60	349.46	238.69	**1.04**	**1.46**
XF_2407	20.56	123.74	94.64	45.78	0.17	2.07
XF_2586	12.86	499.25	262.23	91.39	0.03	2.87
XF_2713	28.79	271.38	153.78	75.45	0.11	2.04
XF_2775	0.38	3.42	8.57	5.76	0.11	1.49
